# The Inheritance of Histone Modifications Depends upon the Location in the Chromosome in *Saccharomyces cerevisiae*

**DOI:** 10.1371/journal.pone.0028980

**Published:** 2011-12-21

**Authors:** Hiroshi Masumoto, Ryuichiro Nakato, Masato Kanemaki, Katsuhiko Shirahige, Mayumi Hachinohe

**Affiliations:** 1 Faculty of Life and Environmental Sciences, Initiative for the Promotion of Young Scientists' Independent Research, University of Tsukuba, Tsukuba, Japan; 2 Laboratory of Genome Structure and Function, Research Center for Epigenetic Disease, The University of Tokyo, Tokyo, Japan; 3 Center for Frontier Research, National Institute of Genetics, Research Organization of Information and Systems, Mishima, Japan; National Cancer Institute, United States of America

## Abstract

Histone modifications are important epigenetic features of chromatin that must be replicated faithfully. However, the molecular mechanisms required to duplicate and maintain histone modification patterns in chromatin remain to be determined. Here, we show that the introduction of histone modifications into newly deposited nucleosomes depends upon their location in the chromosome. In *Saccharomyces cerevisiae*, newly deposited nucleosomes consisting of newly synthesized histone H3-H4 tetramers are distributed throughout the entire chromosome. Methylation of lysine 4 on histone H3 (H3-K4), a hallmark of euchromatin, is introduced into these newly deposited nucleosomes, regardless of whether the neighboring preexisting nucleosomes harbor the K4 mutation in histone H3. Furthermore, if the heterochromatin-binding protein Sir3 is unavailable during DNA replication, histone H3-K4 methylation is introduced onto newly deposited nucleosomes in telomeric heterochromatin. Thus, a conservative distribution model most accurately explains the inheritance of histone modifications because the location of histones within euchromatin or heterochromatin determines which histone modifications are introduced.

## Introduction

The heritability of cell-specific gene regulation maintains that chromatin structures must be propagated across cell generations [1], [2]. The basic unit of chromatin packaging, the nucleosome, consists of 147 bp of DNA wrapped around an octamer of the core histones H2A, H2B, H3 and H4. Each histone is subject to several covalent posttranslational modifications, including acetylation and methylation. Because histone modifications influence several DNA-associated processes, including replication and transcription, these modifications can impact not only the integrity of the chromatin structure but also epigenetic inheritance [2]. The H3-H4 tetramer of each nucleosome is the most stable component and contains consistent and functionally important histone methylation marks. Chromatin is categorized into two transcriptionally distinct regions: euchromatin and heterochromatin. Euchromatin is considered to be the transcriptionally active region. Methylation of lysines 4, 36 and 79 of histone H3 (H3-K4, K36 and K79, respectively) and acetylation of the N-terminal tails of all histones are abundant in the euchromatin in budding yeast [2]. Heterochromatin, which is thought to be regions that are transcriptionally silent, is found at telomeres, the silent mating type loci (*HML*a and *HMR* **a**) and ribosomal DNA repeats in yeast. In contrast to euchromatin, heterochromatin at telomeres and the *HM* loci exhibit hypomethylation and hypoacetylation. Furthermore, DNA elements called silencers recruit the Sir2/3/4 complex, which subsequently spreads along the chromosome for some distance to form higher-order chromatin structures that are characteristic of heterochromatin [3]. Thus, the high-fidelity inheritance of epigenetic chromatin structures across cell generations is required for the correct duplication of histone modification patterns from the mother chromosome to the two daughter chromosomes. However, the molecular mechanism of inheritance of epigenetic chromatin structures remains to be determined.

During DNA replication, preexisting nucleosomes from the parental chromosomes are recycled and deposited onto the newly synthesized daughter DNA strands. Newly synthesized H3-H4 and H2A-H2B dimers are simultaneously deposited onto the chromosome to form new nucleosomes [4], [5]. The daughter chromatin consists of a random mixture of new and old histones in equal amounts, but the newly synthesized histones contain few posttranslational modifications, except for acetylation. The histone methylation modifications involved in epigenetic marking need to be introduced at particular positions within the daughter chromosome to produce an exact duplicate of its parent. A replication-dependent nucleosome partition pattern may promote faithful reproduction of histone modifications within the newly deposited nucleosomes. Therefore, much attention has been focused on the formation of new H3-H4 tetramers on chromatin fibers following the passage of the replication fork. Two models have been proposed for DNA replication-dependent nucleosome partitioning: a conservative distribution model and a semi-conservative distribution model [4], [6], [7], [8]. The conservative distribution model proposes that newly synthesized histone molecules form nucleosomes that are randomly inserted among preexisting parental nucleosomes, which has been supported by early studies [9], [10], [11]. The semi-conservative distribution model proposes that a hybrid nucleosome that contains both newly synthesized and parental histone H3-H4 dimers is formed, which facilitates the transmission of epigenetic information within the basic nucleosome unit. In a human cell, the canonical H3.1 and most of the variant H3.3 are incorporated via the conservative distribution model [12]. In a transcriptionally active gene region, H3.3-H4 tetramers are composed of new and old histones in human cells [12]. In budding yeast, which encode a single isoform of H3, most of the H3-H4 tetramers incorporated into the chromatin fiber during replication are composed of new histone molecules; however, hybrid H3-H4 tetramers composed of new and old histone molecules are incorporated into transcriptionally active regions [13]. Thus, depending on the histone variant and the chromatin region, a newly deposited nucleosome can be formed either via conservative distribution or a mechanism consistent with the semi-conservative model.

It is widely thought that histone modification patterns of newly deposited nucleosomes may be introduced based on the template of histone modifications present on the neighboring preexisting nucleosomes [8]. However, if several newly deposited nucleosomes, formed exclusively of new histone molecules, are assembled sequentially on the chromatin, it is unclear how the histone modification patterns could be correctly copied onto new histone molecules that are potentially located far away from the preexisting nucleosomes. The molecular mechanism that duplicates histone modification patterns onto newly deposited nucleosomes that are composed exclusively of new histone molecules needs to be fully elucidated.

In this study, we show that the introduction of histone modifications into newly deposited nucleosomes depends upon the location of the nucleosome within the chromosome. The majority of newly deposited nucleosomes, which are distributed throughout the entire chromosome, are comprised of new histone H3-H4 tetramers. ChIP-on-chip analysis showed that replication-dependent deposition of new nucleosomes does not always occur in an alternating manner with old nucleosomes. Interestingly, the dimethylation of histone H3-K4 was introduced into these newly deposited nucleosomes, even though the neighboring preexisting nucleosomes harbored a mutation in histone H3 at the K4 site. Furthermore, if the Sir3 was depleted using the anchor-away technique during DNA replication, histone H3-K4 methylation occurred on newly deposited nucleosomes in heterochromatin near the telomere. Thus, a conservative distribution model better explains the inheritance of histone modifications because the location of histones within euchromatin or heterochromatin seems to determine the mechanism by which histone modifications arise.

## Results

### Newly deposited nucleosomes are composed of new histone H3-H4 heterodimers

**Figure 1:**
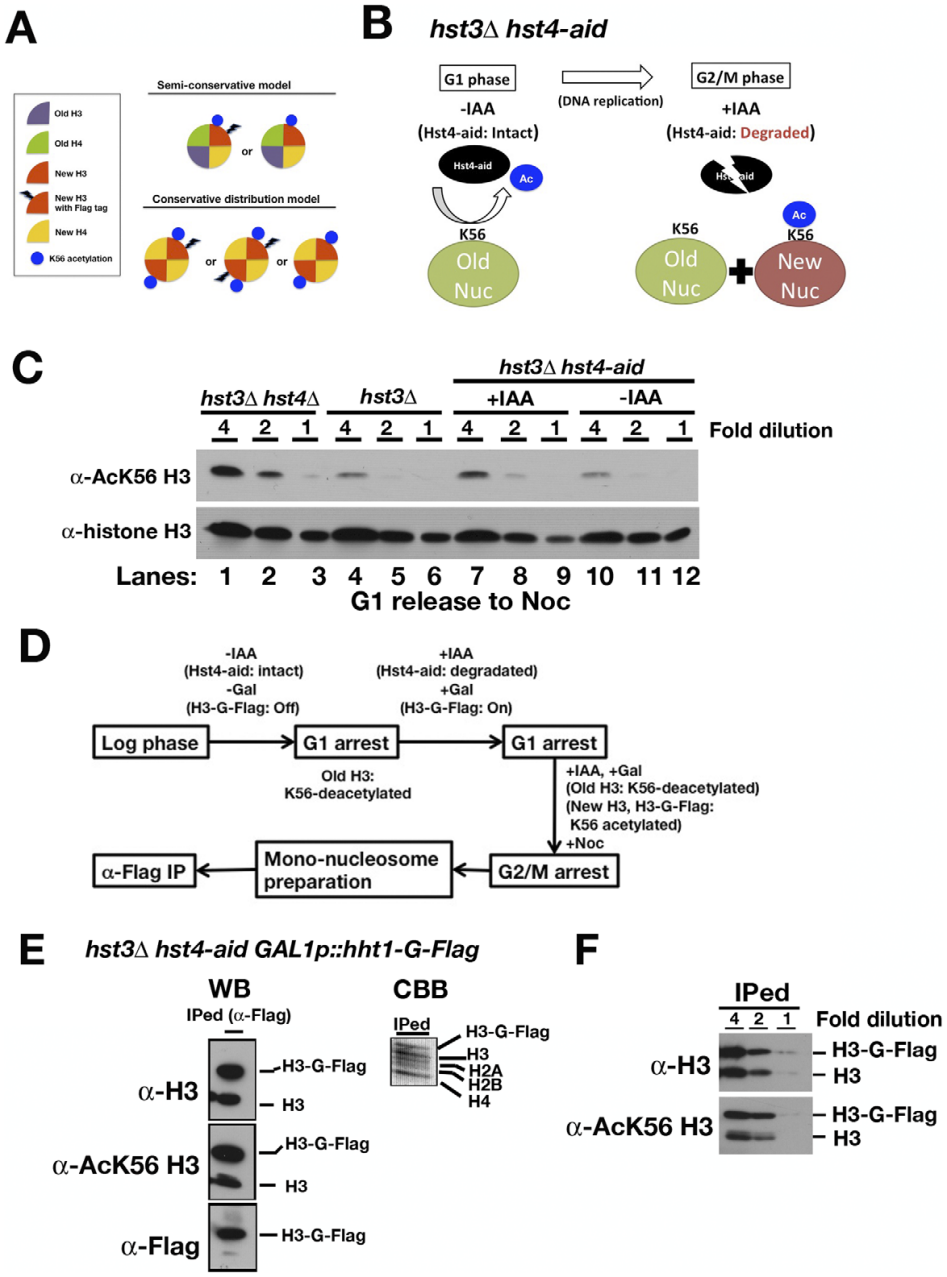
Newly deposited nucleosomes consist mainly of newly synthesized histone molecules. (A) Histone partitioning in the semi-conservative distribution and conservative distribution models. (B) The scheme depicts the experimental procedure used to distinguish between old and new nucleosomes via the AID system and H3-K56 acetylation. Nuc: nucleosome. (C) Immunoblot analysis of whole-cell protein extracts using antibodies against either acetylated K56 of histone H3 or total histone H3 as a loading control. G1-arrested strains [wild-type (W303-1a), *hst3Δ* (HMY210), and *hst3Δ hst4-aid* (HMY837)] were released into YPR medium containing nocodazole. For the *hst3Δ hst4-aid* strain, the cell cultures were treated with or without IAA. Two-fold serial dilutions of each cell extract were resolved by 15% SDS-PAGE and analyzed by immunoblotting using antibodies against K56-acetylated H3 and total histone H3. (D) The scheme shows the procedure employed from cell culture to nucleosome immunoprecipitation. (E) Immunoprecipitated newly deposited nucleosomes were analyzed by SDS-PAGE, and the gels were either stained using Coomassie brilliant blue (CBB) to visualize histone proteins or immunoblotted using antibodies to detect the Flag-epitope, K56-acetylated H3, and total histone H3. (F) Two-fold serial dilutions of the immunoprecipitated nucleosomes in (E) were resolved by 15% SDS-PAGE and analyzed by immunoblotting using antibodies to detect K56-acetylated H3 and total histone H3.

Initially, we examined the histone partitioning pattern during the replication-dependent deposition of new nucleosomes in budding yeast using an approach different from previously described method [13]. Our experimental setup utilized two markers to distinguish newly synthesized histone H3 molecules: a Flag-tagged version of histone H3 under a galactose-inducible promoter and acetylation of histone H3 on lysine 56 (H3-K56), which is specific for newly synthesized histone H3 molecules [14]. If a newly deposited nucleosome consists of a hybrid of new and old histone H3 molecules, the Flag-tagged (new) H3 would be acetylated, and the untagged (old) H3 should be largely unacetylated on K56 (Fig. 1A: Semi-conservative distribution model). If a newly deposited nucleosome consists of only new histone H3, the Flag-tagged H3 would dimerize with either Flag-tagged or untagged endogenous H3, both of which should be acetylated on K56 (Fig. 1A: Conservative distribution model).

Hst3 and Hst4 histone deacetylases sequentially deacetylate histone H3-K56 in chromatin from mid-S phase until G2/M phase and from G2/M phase to the next G1 phase [14], [15], [16]. In this study, old histones would already be deacetylated on H3-K56 at the beginning of the experiment (at G1 phase), whereas newly deposited nucleosomes would remain acetylated due to inactivation of Hst3 and Hst4. Thus, old and new H3 molecules can be distinguished from one another. In the *hst3Δ* deletion strain, Hst4 can deacetylate histone H3-K56 in chromatin until G1 and would also deacetylate K56 of newly synthesized histone H3 during the G2/M phase. Therefore, we constructed a *hst3Δ* strain harboring the Hst4-aid protein, which could be artificially degraded using the auxin-inducible degron (AID) system (*hst3Δ hst4-aid*). The AID system can be used to degrade a target protein upon the addition of indole acetic acid (IAA), a type of plant auxin pheromone [17]. We expected that K56 on histone H3 molecules in newly deposited nucleosomes would remain acetylated after degradation of Hst4-aid by treatment with IAA from the G1 until the G2/M phase (Fig. 1B). We confirmed that the AID system could be used to efficiently degrade the Hst4-aid protein upon treatment with IAA and to prevent the deacetylation of H3-K56 in *hst3Δ hst4-aid* cells (Figure S1). Next, we examined the level of K56 acetylation in total histone H3 molecules at G2/M phase with and without IAA treatment. The acetylation levels of H3-K56 in both *hst3Δ* and *hst3Δ hst4-aid* cells without IAA treatment were slightly lower than that of *hst3Δ hst4-aid* cells with IAA treatment (Fig. 1C; Lanes 4, 5, 7, 8, 10, and 11). In addition, the level of acetylation of H3-K56 in *hst3Δ hst4-aid* cells treated with IAA was maintained at approximately 50% of the level in *hst3Δ hst4Δ* cells (Fig. 1C; Lanes 1, 2, 3, 7, 8, and 9). Based on the fact that almost all of the histone H3 molecules are still acetylated on K56 in the *hst3Δ hst4Δ* strain [15] and that the amount of new nucleosomes in the whole chromosome is equal to the old nucleosomes after DNA replication, we conclude that H3-K56 acetylation is maintained on almost all newly synthesized histone H3 at G2/M phase in *hst3Δ hst4-aid* cells after treatment with IAA.

We examined the acetylation status of histone H3-K56 in new post-replicative mononucleosomes isolated from the *hst3Δ hst4-aid* strain following treatment with IAA. The *hst3Δ hst4-aid* strain encoded a histone H3 gene (*HHT1*) that was C-terminally tagged with the Flag epitope and an intervening glycine linker (H3-G-Flag) under the control of a galactose-inducible promoter. G1-arrested *hst3Δ hst4-aid* cells were incubated in the presence of galactose and IAA to induce both the expression of histone H3-G-Flag and the degradation of the Hst4-aid protein (Fig. 1D). The cells were then cultured until G2/M phase in medium containing IAA, galactose and nocodazole, a tubulin destabilizer to arrest the cells at the G2/M phase (Fig. 1D). Chromatin was prepared and digested with micrococcal nuclease to generate mononucleosomes. Mononucleosomes were fractionated by sucrose gradient ultracentrifugation and subjected to affinity purification using an antibody to the Flag epitope tag (Fig. 1D). Untagged histone H3 molecules were co-immunoprecipitated with histone H3-G-Flag molecules and other histone molecules (H2A, H2B and H4; Fig. 1E; WB of anti-histone H3 and CBB staining). Interestingly, these untagged histone H3 molecules were acetylated on K56 (Fig. 1E; WB of anti-AcK56 H3). Additionally, the ratio of the signal intensity of histone H3-G-Flag and untagged H3 using an anti-histone H3 antibody was similar to the ratio found with the anti-H3-K56Ac antibody (Fig. 1F). Furthermore, we performed the same experiments using the *hst3Δ hst4-aid* strain harboring an N-terminally tagged with the Flag epitope histone H3 (Flag-H3) under the control of a galactose-inducible promoter (Figure S2). In immunoprecipitated mononucleosomes containing Flag-H3, almost all histone H3 molecules were Flag-H3, and a small population of untagged histone H3 in the immunoprecipitate was acetylated on K56 (Figure S2). We also confirmed that histone H3-G-Flag was barely detectable in the chromatin fraction if free histone H3-G-Flag was expressed in G2/M-arrested cells (Figure S3). This indicates that histone H3-G-Flag molecules were not incorporated into nucleosomes by exchange between excess free histone H3-G-Flag molecules and untagged histone H3 in nucleosomes during the G2/M phase. These results support the conservative distribution model that newly deposited nucleosomes are composed of new histone molecules in *Saccharomyces cerevisiae*.

### Newly deposited and preexisting nucleosomes do not always alternate with each other during deposition on chromatin fiber

**Figure 2:**
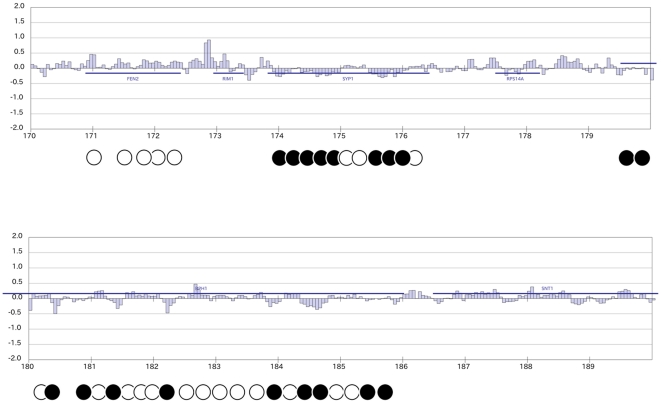
Newly deposited nucleosomes and preexisting nucleosomes are not always deposited on the chromatin fiber in an alternating manner. ChIP-on-chip analysis was conducted using mononucleosomes harboring the histone H3-G-Flag prepared previously (Figure 1E). Empty and filled circles represent the positions of newly deposited and preexisting nucleosomes, respectively. The map shows the 20 kbp region from 170 kbp to 190 kbp on the right arm of chromosome III. The horizontal lines indicate the log 1 of the signal strength, and the vertical scale bar indicates the chromosomal coordinates in kb. Blue horizontal lines indicate the open reading frames (ORFs); the ORFs above the horizontal line are oriented in the 5′ to 3′ direction from left to right, and the genes below are oriented in the reverse direction. A single bar of signal indicates 50 bp, and a set of three bars is approximately equivalent to one nucleosome (∼160 bp).

To correctly copy histone modification patterns from preexisting to newly deposited nucleosomes, it has been widely accepted that a new nucleosome should be deposited between or within a close distance from preexisting nucleosomes. To examine the pattern of distribution of newly deposited nucleosomes over the entire yeast genome during DNA replication, we employed the chromatin immunoprecipitation (ChIP)-on-chip assay. Nucleosomal DNA from new nucleosomes harboring histone H3-G-Flag, as depicted in Fig. 1E, was examined in different locations within the genome. At many gene loci, old and new nucleosomes are distinctly localized after replication [13]. However, we cannot exclude the possibility that nucleosomes containing histone H3-G-Flag might be composed of new and old histone molecules. A representative distribution map of new nucleosomes in a 20 kbp region from 170 kbp to 190 kbp on the right arm of chromosome III is shown in Fig. 2. Figure S4 shows the distribution map of new nucleosomes throughout the entire yeast genome. Interestingly, the positive signals were not evenly distributed throughout the whole chromosome (Figs. 2 and S4). Our interpretation of this result is that an accumulation of positive signals represents new nucleosomes deposited on the chromatin fiber, whereas an array of negative signals indicates the assembly of preexisting “old” nucleosomes (Figs. 2 and S4). Either old or new nucleosomes tended to accumulate at a particular chromatin position. At the *FEN2* locus, several positive signals were detected, which indicates that new nucleosomes were deposited contiguously (Fig. 2;upper column). In contrast, at the *SYP1* locus, negative signals were continuously detected, which indicates that old nucleosomes clustered at this locus (Fig. 2: upper column). Furthermore, in parts of the *BPH1* locus, positive and negative signal clusters were found to alternate with each other, which means that new nucleosomes were deposited at positions adjacent to old nucleosomes (Fig. 2: lower panel). It is worth noting that although the majority of randomly deposited nucleosomes harboring the H3-G-Flag is not likely to be due to histone exchange, a small amount of free histone H3-G-Flag may be incorporated into nucleosomes without DNA replication (Figure S3A, B and C). We also confirmed that free histone H3-G-Flag did not show preferential accumulation outside S phase on the *FEN2* gene and several other loci where new nucleosomes are deposited contiguously during S-phase (Figures S3D and S4). Thus, newly deposited and preexisting nucleosomes are not always deposited on chromatin fibers in an alternating manner. New nucleosomes were generally distributed throughout gene regions, including promoter and terminator regions (Figure S4), but at several genes, new nucleosomes tended to accumulate in promoter regions (Figure S4; e.g., *TUB2* locus in Chr. IV and the *PAB1* locus in Chr. V). No clear relationship was found between active gene transcription and the tendency for new nucleosomes to cluster at promoter regions. We also examined the deposition of new nucleosomes around the replication origins, but no obvious accumulation of either new or old nucleosomes was observed in close proximity to active origins (Figure S4; the ARS606 and ARS607 loci in Chr. VI [18]). Based on these results, we conclude that new nucleosomes can be deposited into chromatin continuously following the progression of either leading or lagging strand synthesis during DNA replication.

### Histone modification patterns of newly deposited nucleosomes are determined by their location within the chromosome

**Figure 3:**
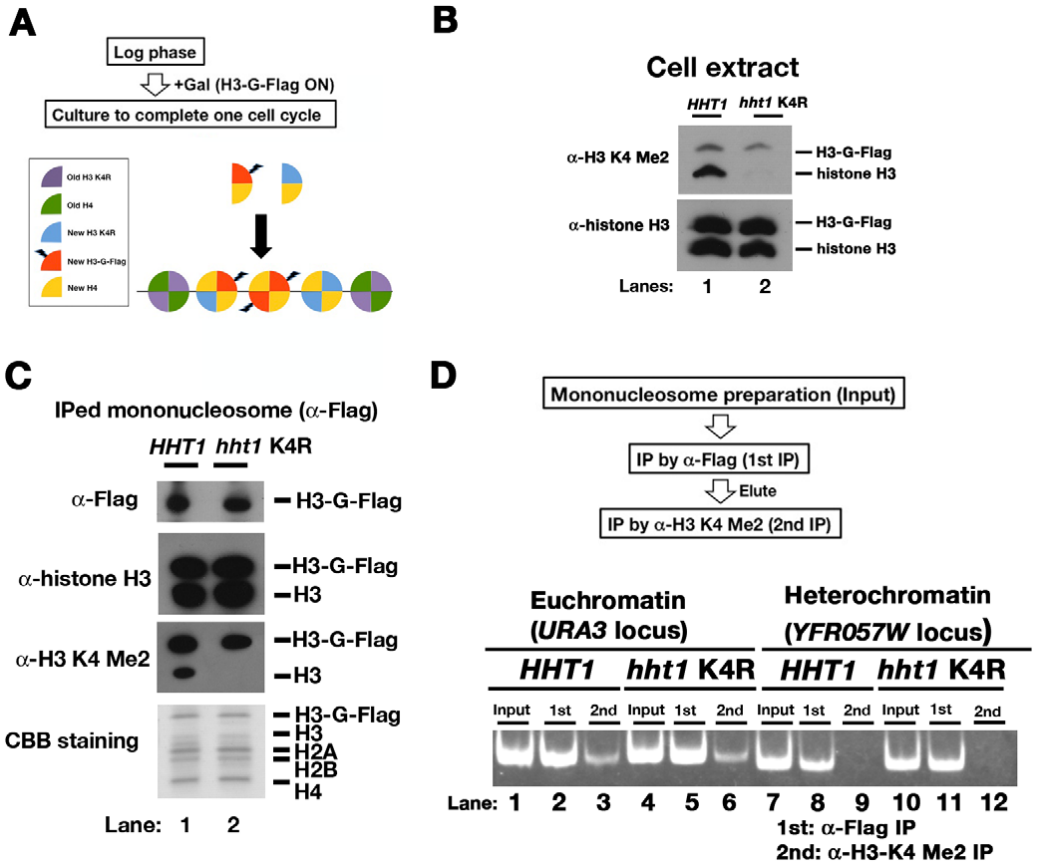
Methylation of histone H3-K4 in newly deposited nucleosomes does not depend on the neighboring preexisting nucleosomes. (A) The scheme presents the cell culture and histone H3-H4 partitioning pattern in new nucleosome using H3-G-Flag and H3-K4R according to the conservative distribution model. (B) Immunoblot analysis of whole-cell protein extracts using antibodies against either K4-dimethylated histone H3 or total histone H3 as a loading control. (C) Immunoblot analysis of mononucleosomes affinity purified using antibodies against the Flag epitope, K4-dimethylated histone H3 and total histone H3. CBB staining shows the histone proteins. (D) DNA isolated from the affinity-purified mononucleosomes described in (C) was analyzed by PCR using primers specific for either the *URA3* or *YFR057W* locus, which are located in the subtelomeric region of chromosome VI [38].

We examined whether the histone modifications of a new nucleosome would be replicated based on the histone modifications present on neighboring preexisting nucleosomes. As an epigenetic mark of euchromatin, we examined H3-K4 dimethylation. In budding yeast, histone H3-K4 dimethylation appears to spread throughout genes in euchromatin [19], [20], [21]. We tested whether histone H3-K4 dimethylation occurred on newly deposited nucleosomes in euchromatin, even if histone H3-K4 dimethylation in the neighboring preexisting nucleosomes was absent. Two strains containing only one gene for histone H3 were constructed: one harbored the wild-type *HHT1* gene encoding the sole histone H3 and the other carried a mutant *hht1* gene in which lysine 4 had been mutated to arginine (K4R). In addition, these strains contained histone H3-G-Flag under a galactose-inducible promoter. Histone H3-K4 dimethylation, together with H3-K79 methylation, was introduced into newly synthesized histone molecules after mid-S phase (Figure S5). Asynchronous cells were cultured in the presence of galactose to induce expression of histone H3-G-Flag until the cell numbers doubled, which indicated that the cells had completed one cell cycle (Fig. 3A). Whole cell extracts were prepared and resolved by sodium dodecyl sulfate (SDS)-15% polyacrylamide gel electrophoresis (PAGE), and two species of histone H3 molecules (H3-G-Flag and untagged H3) and histone H3-K4 methylation (dimethylated form; H3-K4 Me2) were detected by western blotting with an anti-histone H3 antibody and an anti-histone H3-K4 Me2-specific antibody, respectively. H3-K4 dimethylation was detected in wild-type histone H3 but not in the histone H3 K4R mutant (Fig. 3B; Lanes 1 and 2). Surprisingly, K4 dimethylation in histone H3-G-Flag was detected not only in the wild-type strain but also in the *hht1* K4R mutant strain (Fig. 3B; Lanes 1 and 2). This result suggests that histone H3-K4 methylation in a newly deposited H3 molecule was not influenced by the lack of H3-K4 methylation in preexisting nucleosomal histones.

We examined whether introducing K4 dimethylation into histone H3-G-Flag would depend on the location of the nucleosome on the chromosome or on the dimethylation status of neighboring preexisting nucleosomes. We confirmed that no difference in the composition of affinity-purified mononucleosomes was found between the wild-type *HHT1* and *hht1* K4R mutant strains upon introduction of K4 dimethylation on histone H3-G-Flag (Fig. 3C; Lanes 1 and 2). These mean that the histone H3 K4R mutation does not influence the assembly of new nucleosomes or the dimethylation of K4 in wild type histone H3 within the same nucleosome. Mononucleosomes containing K4-dimethylated histone H3-G-Flag were prepared by sequential affinity purification with antibodies to the Flag epitope and then with antibodies to H3-K4 Me2 (Fig. 3D: diagram of the procedure). The localization of the purified mononucleosomes in either the *URA3* locus (euchromatin) or the *YFR057W* locus (heterochromatin) was identified by PCR. At the *URA3* locus, histone H3-G-Flag was dimethylated on K4 in both the wild-type *HHT1* and *hht1* K4R strains (Fig. 3D; Lanes 3 and 6) but not at the *YFR057W* locus (Fig. 3D; Lanes 9 and 12). This result indicates that introduction of histone H3-K4 dimethylation onto newly deposited nucleosomes occurs specifically in euchromatin, even though the neighboring preexisting nucleosomes lack methylation of histone H3-K4 within euchromatin. Thus, the histone modification pattern found in newly deposited nucleosomes is not copied from the methylation patterns of preexisting nucleosomes during DNA replication but is determined by their location within the chromosome.

### Histone-modifying enzymes and the Sir2/3/4 complex determine the introduction of epigenetic histone modifications in newly deposited nucleosomes

**Figure 4:**
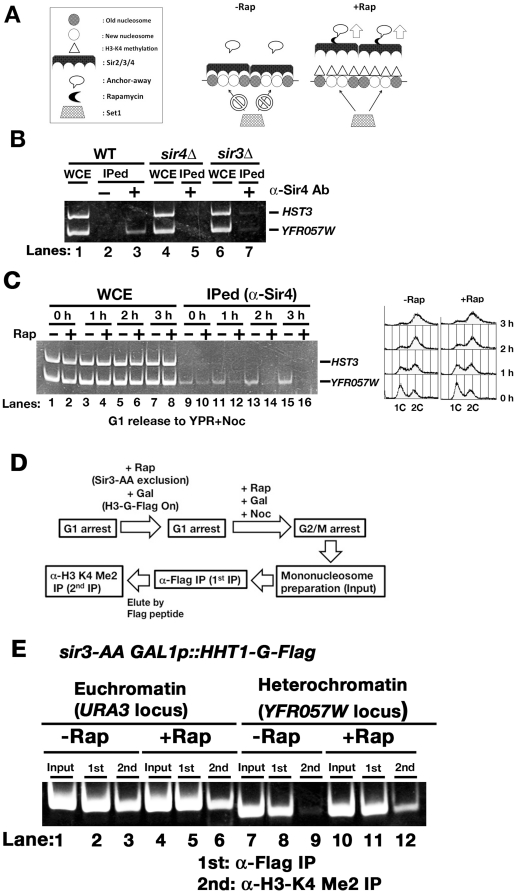
Histone H3-K4 is methylated in newly deposited nucleosomes within heterochromatin in the absence of Sir3. (A) The scheme summarizes the experimental approach employed to make “open” chromatin in heterochromatic regions by removing the Sir2/3/4 complex using the anchor-away technique. (B) Chromatin immunoprecipitation (ChIP) analysis was applied to examine the localization of the Sir4 protein in heterochromatin. DNA isolated from immunoprecipitated chromatin (IPed) or whole-cell extracts (WCE) was quantitatively analyzed using a competitive PCR strategy, in which one set of primers amplified 80- and 155-bp products from the *YFR057W* locus and the *HST3* locus, respectively. (C) ChIP analysis was used to monitor the localization of the Sir4 protein in heterochromatin in the *sir3*-AA strain. G1-arrested cells were treated with or without rapamycin and then released into YPR medium containing nocodazole with or without rapamycin. Cells were harvested at 1 hr intervals and fixed with formaldehyde. DNA was prepared and analyzed by PCR as described in (B). (D) A schematic of the procedure employed from cell culture to immunoprecipitation of mononucleosomes is shown. (E) DNA isolated from affinity-purified mononucleosomes, as described in (C), was analyzed by PCR using primers specific for either the *URA3* or *YFR057W* locus, as described in Fig. 3D.

Hypomethylation of new nucleosomes deposited in heterochromatin would be contingent on blocking the access of histone methylases to heterochromatin loci by heterochromatin-binding proteins. If so, histone methylases could methylate new nucleosomes in heterochromatin by removing heterochromatin-binding protein from chromatin following DNA replication. We tested whether histone H3-K4 dimethylation would be introduced into newly deposited nucleosomes in heterochromatin after the Sir3 was removed from the heterochromatin using the anchor-away (AA) technique (Fig. 4A). The AA technique depletes the nucleus of a protein of interest by conditional tethering to an abundant cytoplasmic protein by appropriate gene tagging and rapamycin-dependent heterodimerization [22]. The *sir3*-AA strain, in which the Sir3 protein can be excluded from the nucleus by the AA technique, has already been demonstrated to cause a defect in gene silencing at telomere loci in the presence of rapamycin [22]. The Sir2/3/4 complex does not stably bind to heterochromatin in the absence of Sir3 [23], [24]. ChIP analysis confirmed that Sir4 was specifically bound to heterochromatin (the *YFR057W* locus; Fig. 4B; Lanes 1, 2, and 3) and did not remain bound at the *YFR057W* locus in the *sir3Δ* strain (Fig. 4B; Lanes 3, 5, and 7). Next, we examined whether Sir4 would be depleted from heterochromatin from G1 to G2 phases of the cell cycle in the *sir3*-AA strain with or without treatment with rapamycin. Without rapamycin treatment, Sir4 remained bound to the *YFR057W* locus throughout the time course analyzed (Fig. 4C; Lanes 9, 11, 13, 15 and FACS plot). Following treatment with rapamycin, Sir4 was lost from the *YFR057W* locus after DNA replication (Fig. 4C; Lanes 10, 12, 14, 16 and FACS plot). Although FACS analysis showed that a small population of cells remained in G2 (Fig. 4C), this is likely to be simply due to a delay in cell cycle progression as a result of using raffinose as the carbon source for cell culture (Fig. 4C). We confirmed that Sir4 could not be lost from heterochromatin both at G1 and G2/M arrested *sir3*-AA cells with or without treatment of rapamycin (Figure S6). And also, we confirmed that histone H3-K4 dimethylation was not introduced into heterochromatin in the G1-arrested *sir3*-AA strain by treatment with rapamycin (Figure S7). Thus, the Sir2/3/4 complex can be efficiently removed from heterochromatin using the AA technique following DNA replication.

We examined whether the normally euchromatic dimethylation of histone H3-K4 can be introduced into new nucleosomes deposited in heterochromatin after the removal of the Sir2/3/4 complex. For this we maintained the expression of histone H3-G-Flag under a galactose-inducible promoter in the *sir3*-AA strain. Then, G1-arrested *sir3*-AA cells were treated with or without rapamycin in the presence of galactose (Fig. 4D). The cells were then released from G1-arrest and cultured until G2/M phase in medium containing galactose and nocodazole in the presence or absence of rapamycin (Fig. 4D). Mononucleosomes containing histone H3-G-Flag that were dimethylated at H3-K4 were prepared by sequential affinity purification with antibodies to the Flag epitope and then with antibodies to H3-K4 Me2. Nucleosomal DNA was prepared and analyzed by PCR to identify the location of the nucleosomes on the chromosome. Without rapamycin treatment, dimethylated histone H3-K4 was detected at the *URA3* locus but not at the *YFR057W* locus (Fig. 4E; Lanes 3 and 9). In contrast, after treatment with rapamycin, dimethylated histone H3-K4 was detected not only at the *URA3* locus but also at the *YFR057W* locus (Fig. 4E; Lanes 6 and 12). These data support our hypothesis that the Sir2/3/4 complex blocks Set1 from accessing newly deposited nucleosomes in heterochromatin, thereby preventing the dimethylation of histone H3 on K4 at these sites, whereas Set1 is able to access new nucleosomes in euchromatin due to the absence of the Sir2/3/4 complex. Apart from excluding the Set1 mediated *de novo* H3 K4 methylation in heterochromatin, it is possible that Sir proteins may also somehow exclude the pre-existing methylated H3 K4 from being deposited in heterochromatic regions during the random distribution of pre-existing histones during S-phase.

## Discussion

In this study, we examined the inheritance of histone modification patterns in newly deposited nucleosomes after DNA replication in budding yeast. In general, our data support the notion that the majority of newly deposited nucleosomes are composed of new histone H3 molecules via the conservative distribution model. We cannot exclude that a small fraction of newly deposited nucleosomes are composed of a hybrid of old and new histone H3 molecules in a manner consistent with the semi-conservative model. We employed acetylation of histone H3 on K56 to distinguish whether untagged histone H3 molecules in newly deposited nucleosomes containing a Flag epitope-tagged histone H3 were new or old (Fig. 1). Approximately 10% of old histone H3 molecules remain acetylated on K56 prior to S phase [15], and this level was barely detectable by immunoblotting with the anti-histone H3-K56 acetylation antibody used in this study [14]. If an untagged histone H3 in preexisting nucleosomes remains acetylated on K56 and is used in a newly deposited nucleosome, our approach cannot be used to distinguish whether newly deposited nucleosomes are synthesized according to the semi-conservative model or the conservative distribution model. However, even if K56-acetylated histone H3 molecules in preexisting nucleosomes were assembled into new nucleosomes, they would occupy less than 10% of the total nucleosomes. Thus, our data suggest that the majority of newly deposited nucleosomes are assembled by the conservative distribution model, in support of the results obtained in a previous study [13].

**Figure 5:**
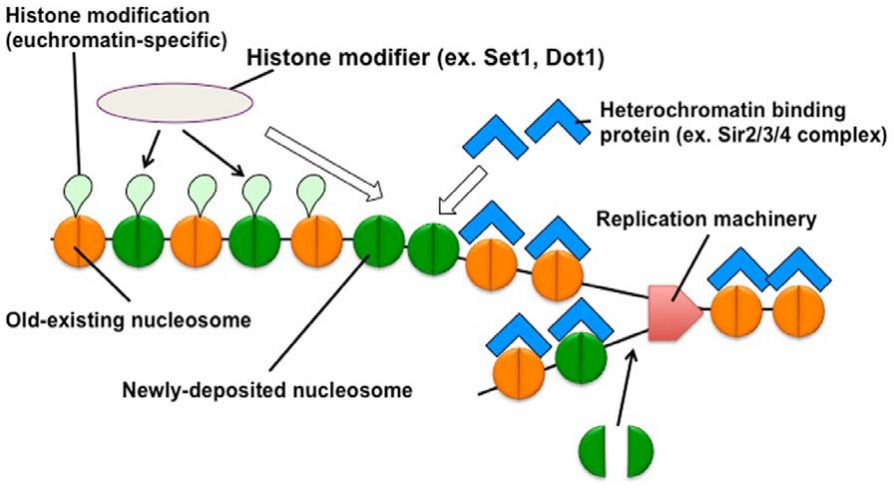
Model depicting the replication of epigenetic histone modification patterns in chromatin.

Recently, the Rando group has shown that maternal histones re-associate close to their original locations on daughter genomes after replication [25]. They suggest that the re-association of maternal histones can transmit the histone modification pattern that maternal histones possess onto the same locations on daughter genomes [25]. Our findings are consistent with this idea. We found that the methylation status of a newly deposited nucleosome does not depend on that of the neighboring preexisting nucleosomes, but rather upon the location of the nucleosome within the chromosome. We propose a model in which histone modification patterns for methylation in newly deposited nucleosomes are replicated according to their location in the chromosome, which is regulated by the activities of histone-modifying enzymes and the Sir2/3/4 complex (Fig. 5). This model is based on the different distributions of histone modifications in regions of euchromatin and heterochromatin along the chromosome arms; euchromatin regions are hypermethylated and hyperacetylated at certain sites, whereas heterochromatin regions are hypomethylated and hypoacetylated [26], [27], [28]. In euchromatin, histone-modifying enzymes access the chromatin and modify histones in newly deposited nucleosomes. In telomeric heterochromatin, the Sir2/3/4 complex quickly binds to newly assembled chromatin and blocks histone-modifying enzymes from accessing a particular region. It is likely that the Sir2/3/4 complex cannot access euchromatin because the preexisting nucleosomes have already been methylated on each of the three lysine residues in histone H3 (K4, K36 and K79) and hyperacetylated. Thus, our findings might explain why the histone modification patterns are faithfully duplicated at different chromosomal loci, even when several new nucleosomes may be clustered together in a contiguous manner, and thus may not be near preexisting nucleosomes.

In this study, we investigated the replication of histone modification patterns in euchromatin and heterochromatin in budding yeast. Histone methylation (H3-K4, –K36, and –K79) has been found in euchromatin in other eukaryotes [2], and these modifications are expected to be replicated in a manner similar to budding yeast. In contrast to budding yeast, the methylation of histones H3-K9, H3-K27, and H4-K20 is correlated with the imprinting of transcriptionally silent chromatin in other eukaryotes [2], [29], [30]. Furthermore, semi-conservative replication of histone modification patterns is also known to occur in some cases, as suggested by the duplication of histone H3-K9 methylation in heterochromatin, which is transmitted from the neighboring preexisting nucleosomes to newly deposited nucleosomes [31]. Further analyses will be necessary to elucidate the molecular mechanisms involved in the replication of epigenetic histone modification patterns in other eukaryotes.

## Materials and Methods

### Plasmids and strains

The plasmids and strains that were used are listed in the Method S1. The plasmid encoding histone H3 fused to a G linker-Flag epitope tag [a glycine linker (G linker) with a Flag epitope tag (F)] was constructed using the previously described G linker sequence [22]. The C-terminal FH-tagged *HHT1* gene fusion was prepared using the polymerase chain reaction (PCR) with a plasmid containing the *HHT1* gene as a template and then subcloned into the *Saccharomyces cerevisiae* integration vector YIplac204 to yield plasmid PHM371 [32]. Two additional plasmids, PHM493 and PHM502, were generated by subcloning histone H3 (*HHT1*) or histone H3 with a substitution of lysine 4 for an arginine (*hht1* K4R), respectively, along with the native *HHT1* promoter into the expression vector pRS413 [32]. All DNA sequences were verified.

The parental *Saccharomyces cerevisiae* strain used in this study was W303-1a [33]. The genotypes of strains used are listed in the Method S1. We adapted a PCR-based procedure for the disruption of target genes and IAA17- or FRB-domain tagging at the carboxyl terminus of endogenous genes in yeast chromosomes [34], [35]. All constructs were confirmed by PCR amplification. Because the *hst3Δ hst4Δ* double deletion strain harbors the PHM286 *URA3* plasmid (which encodes wild-type *HST3* and prevents spontaneous DNA damage and genomic instability), we counter-selected cells for loss of the PHM286 plasmid by the addition of 5-fluoroorotic acid (5-FOA) prior to usage.

### Yeast cell culture (auxin-inducible degron technique for Hst4-aid)

Asynchronous HMY837 cells (5×10^6^ cells/ml) in 500 ml aliquots of YPR liquid medium (1% Bacto yeast extract (Difco) and 2% Bacto polypeptone (Difco) with 2% raffinose) were cultured at 25°C for 2 hr in the presence of a final concentration of 10 µg/ml α-factor to arrest the cells at the late G1 phase. G1-arrested cells were further incubated for 1 hr at 25°C in the presence of 2% galactose and α-factor at a final concentration of 10 µg/ml with 1 mM indole acetic acid (IAA). Cells were released from the growth arrest using 500 ml of YPR plus 2% galactose liquid medium containing 10 µg/ml nocodazole with 1 mM IAA and cultured at 30°C for 3 hr. The cells were fixed in 0.01% sodium azide for 5 min at 4°C, collected by centrifugation, and stored at −80°C.

### Yeast cell culture (anchor-away technique for Sir3-AA)

Asynchronous HMY733 (5×10^6^ cells/ml) cells were cultured in two 500 ml aliquots of YPR liquid medium and incubated at 25°C for 2 hr in the presence of a final concentration of 10 µg/ml α-factor to arrest the cells at late G1 phase. The G1-arrested cells were further incubated for 1 hr at 25°C in the presence of 2% galactose and α-factor at a final concentration of 10 µg/ml with or without rapamycin at a final concentration of 1 µg/ml. The cells were released from the growth arrest using 500 ml of YPRG (YPR with 2% galactose) liquid medium containing a final concentration of 10 µg/ml nocodazole with or without rapamycin at a final concentration of 1 µg/ml and cultured at 30°C for 3 hr. The cells were fixed in 0.01% sodium azide for 5 min at 4°C, collected by centrifugation, and stored at −80°C.

### Mononucleosome immunoprecipitation (IP)-reIP

The methods used for the preparation of yeast chromatin and isolation of mononucleosomes are described in the Method S1. Mononucleosome fractions were incubated with 50 µl of Anti-FLAG M2 Affinity Gel (Sigma) for 2 hr with rotation at 4°C. The beads were washed three times with 500 µl of wash buffer (10 mM Tris-Cl, pH 7.5/1 mM EDTA/150 mM NaCl/0.01% Tween 20), and the mononucleosomes containing Flag-tagged histone H3 were eluted in 100 µl of wash buffer containing 1 mg/ml 3× Flag peptide (Sigma) and then incubated at 4°C for 30 min. The eluted fraction was diluted to 400 µl with wash buffer, and 50 µl of this dilution was further diluted with wash buffer to 400 µl (first IP). The remaining eluted fraction was mixed with 2 µl of Anti-di-methyl K4 histone H3 antibody (Abcam, United Kingdom) and incubated with rotation for 2 hr at 4°C. The beads were washed three times with 500 µl of wash buffer, and DNA was isolated using the Wizard DNA clean-up kit (Promega) and then suspended in 50 µl of DIW (second IP). Reaction mixtures were prepared and PCR amplification was performed according to the manufacturer's instructions (HybriPol DNA polymerase, Bioline and Taq HS, Takara; these systems equally worked). The primer sequences used are listed in the Method S1.

### Chromatin immunoprecipitation (ChIP) assay

Yeast cultures of 25 ml (at 0.5–1.0×10^7^ cells/ml) were crosslinked with 1% formaldehyde for 10 min at room temperature. After quenching the formaldehyde by the addition of glycine at a final concentration of 0.125 M, the cells were washed with TBS (20 mM Tris-HCl, pH 7.5/0.15 M NaCl) containing 0.125 M glycine. Chromatin preparation was performed as described previously [36]. Prepared chromatin was sheared by sonication using a Biorupter (CosmoBio, Japan) according to the instruction manual. One milligram of sheared chromatin was incubated with 0.2 mg of anti-Sir4 antibody (Santa Cruz: Y-300) with rotation for 3 hr at 4°C. The mixture was then incubated with a 5 ml bed volume of Dynabeads Protein G (Invitrogen) for 1 hr at 4°C. Dynabeads washing and DNA recovery from the beads were performed as described previously [36]. DNA fragments were cleaned up using the Qiaquick PCR Purification Kit (Qiagen). Reaction mixtures were prepared and PCR amplification was performed according to the manufacturer's instructions (HybriPol DNA polymerase, Bioline and Taq HS, Takara). The primer sequences used are listed in the Method S1.

### Quantitative PCR (qPCR) assay and ChIP-on-chip analysis

SYBR *premix EX Taq* II (Takara) and a TP850 RT PCR machine (Takara) were employed for qPCR. The reaction mixtures were prepared and qPCR amplification was performed in the PCR machine according to the manufacturer's instructions. ChIP-on-chip analysis was performed as described previously [37].

## Supporting Information

Figure S1 **AID system prevents the deacetylation of histone H3-K56 by promoting Hst4-aid degradation in** ***hst3Δ*** **strain.** Cell extracts were prepared from each strain (Wild type (W303-1a), *hst3Δ hst4-aid* (HMY837) and *hst3Δ hst4Δ* (HMY278)) arrested at each cell cycle stage (α-arrest (G1), Nocodazole-arrest (G2/M), and Log phase). *hst3Δ hst4-aid* cells were additionally treated with or without IAA, The histone H3-K56 acetylation and the total amount of histone H3 were analyzed by immunoblot using antibody to AcK56 H3 and histone H3, respectively. Cell cycle arrest was monitored by FACS analysis. We confirmed that H3-K56 remained acetylated at G1 phase in *hst3Δ hst4-aid* cell with treatment of IAA, but H3-K56 had been deacetylated without IAA (Lanes 5 and 8).
(TIF)

Figure S2 **Histone H3-H4 partition in newly deposited nucleosome is composed of newly synthesized nucleosome using N-terminal Flag-tagging histone H3.** (A) The experimental procedure of isolation of newly deposited nucleosome using N-terminal Flag-tagging histone H3 (Flag-H3). (B) Immunoprecipitated newly deposited nucleosome containing Flag-H3 was separated by SDS-PAGE, and stained by CBB staining to visualize histone proteins, or transferred to a nitrocellulose membrane. Western blotting analysis with Flag-H3, K56-acetylated H3 and whole histone H3, respectively, is shown.
(TIF)

Figure S3 **Histone exchange with free histone H3-G-Flag and histone H3 in chromatin at G2/M-arrested cell.** (A) A scheme of procedure of induction of histone H3-G-Flag in G2/M arrest cell. HMY616 cells were arrested at G2/M phase in YPR medium containing nocodazole at a final concentration of 10 µg/ml, and then further treated with nocodazole and benomyl at final concentrations of 5 µg/ml and 20 µg/ml, respectively, in the presence of 2% galactose at 25°C for 3 hr [2]. (B) Chromatin was isolated from cells and analyzed by immunoblot using antibody to Flag epitope and histone H3, respectively. (C) Amounts of histone H3-G-Flag and histone H3 in immunoblot using anti-histone H3 antibody (B) were quantified by Image J software (NIH, USA). (D) ChIP-quantitative PCR analysis using anti-Flag antibody for association of free histone H3-G-Flag at different gene loci. Chromatin was prepared from G2/M arrested HMY616 cells. The graphs represent the average and standard deviation of two independent experiments.
(TIF)

Figure S4 **The distribution map of affinity-purified new nucleosomes harboring histone H3-G-Flag analyzed by ChIP-on-chip analysis.** Blue horizontal lines indicate the open reading frames, and positive orange peaks indicate the significant binding of the proteins to the chromosome. CEN denotes the position of the centromere, and the red lines and numbers indicate the positions of autonomously replication origins (ARS). The horizontal lines indicate log 1 of the signal strength, and the vertical scale bar indicates the chromosomal coordinates in kb.
(PDF)

Figure S5 **Histone methylations specific for euchromatin are introduced into new nucleosome after mid-S phase.** G1-arrested cells expressing histone H3-G-Flag were released into YPR medium containing nocodazole and galactose. Cell-cycle progression was monitored by FACS analysis. Cell extracts prepared from cells at each time were analyzed by SDS-PAGE and gels were transferred to a nitrocellulose membrane. Western blotting analyses with antibodies to histone H3 dimethylated at -K4, -K79, and whole histone H3 are shown. H3-K4 and H3-K79 di-methylation were detected in histone H3-G-Flag after 80 min in time course (during mid-S phase).
(TIF)

Figure S6 **Sir4 remains bound on heterochromatin in both at G1- and G2/M-arrested** ***sir3*** **-AA cells with rapamycin.** (A) A scheme of procedure of treatment of rapamycin both with G1- and G2/M-arrested cells. 1. HMY733 cells were arrested at G1 phase in YPR medium containing α-factor at a final concentration of 10 µg/ml, and then further treated with 10 µg/ml α-factor in the absence or presence of 1 µg/ml rapamycin at 25°C for 1 hr. 2. HMY733 cells were arrested at G2/M phase in YPR medium containing nocodazole (Noc) at a final concentration of 10 µg/ml, and then further treated with 10 µg/ml nocodazole, 20 µg/ml benomyl, in the absence or presence of 1 µg/ml rapamycin at 30°C for 1 hr. (B) Chromatin immunoprecipitation (ChIP) analysis was applied to examine the localization of the Sir4 protein in heterochromatin. DNA isolated from immunoprecipitated chromatin (IPed) or whole-cell extracts (WCE) was quantitatively analyzed using a competitive PCR strategy, in which one set of primers amplified 80- and 155-bp products from the *YFR057W* locus and the *HST3* locus, respectively.
(TIF)

Figure S7 **Rapamycin treatment to G1-arrested** ***sir3*** **-AA cells does not induce histone H3-K4 di-methylation on heterochromatin.** (A) The experimental procedure for the isolation of mononucleosomes containing Flag-tagged dimethylated histone H3-K4. (B) The localization of DNA isolated from affinity-purified mononucleosomes was analyzed by PCR as described in Fig. 3D. With or without rapamycin, histone H3-K4 di-methylation was detected at *URA3* locus, but not at *YFR057W* locus (Lanes 2, 4, 6, and 8).
(TIF)

Methods S1 **Methods of preparations of yeast chromatin and mononucleosome, primer sequences, plasmids and yeast strains.**
(DOCX)

## References

[1] Wolffe AP (1999). Chromatin: Structure and Function, 3rd Ed.

[2] Kouzarides T (2007). Chromatin modifications and their function.. Cell.

[3] Rusche LN, Kirchmaier AL, Rine J (2003). The establishment, inheritance, and function of silenced chromatin in Saccharomyces cerevisiae.. Annu Rev Biochem.

[4] Annunziato AT (2005). Split decision: what happens to nucleosomes during DNA replication?. J Biol Chem.

[5] Tagami H, Ray-Gallet D, Almouzni G, Nakatani Y (2004). Histone H3.1 and H3.3 complexes mediate nucleosome assembly pathways dependent or independent of DNA synthesis.. Cell.

[6] Henikoff S, Furuyama T, Ahmad K (2004). Histone variants, nucleosome assembly and epigenetic inheritance.. Trends Genet.

[7] Groth A, Rocha W, Verreault A, Almouzni G (2007). Chromatin challenges during DNA replication and repair.. Cell.

[8] Cheung P, Lau P (2005). Epigenetic regulation by histone methylation and histone variants.. Mol Endocrinol.

[9] Jackson V, Chalkley R (1985). Histone segregation on replicating chromatin.. Biochemistry.

[10] Sogo JM, Stahl H, Koller T, Knippers R (1986). Structure of replicating simian virus 40 minichromosomes. The replication fork, core histone segregation and terminal structures.. J Mol Biol.

[11] Sugasawa K, Ishimi Y, Eki T, Hurwitz J, Kikuchi A (1992). Nonconservative segregation of parental nucleosomes during simian virus 40 chromosome replication in vitro.. Proc Natl Acad Sci U S A.

[12] Xu M, Long C, Chen X, Huang C, Chen S (2010). Partitioning of histone H3-H4 tetramers during DNA replication-dependent chromatin assembly.. Science.

[13] Katan-Khaykovich Y, Struhl K (2011). Splitting of H3-H4 tetramers at transcriptionally active genes undergoing dynamic histone exchange.. Proc Natl Acad Sci U S A.

[14] Masumoto H, Hawke D, Kobayashi R, Verreault A (2005). A role for cell-cycle-regulated histone H3 lysine 56 acetylation in the DNA damage response.. Nature.

[15] Celic I, Masumoto H, Griffith WP, Meluh P, Cotter RJ (2006). The sirtuins Hst3p and Hst4p preserve genome integrity by controlling histone H3 lysine 56 deacetylation.. Curr Biol.

[16] Maas NL, Miller KM, DeFazio LG, Toczyski DP (2006). Cell cycle and checkpoint regulation of histone H3 K56 acetylation by Hst3 and Hst4.. Mol Cell.

[17] Nishimura K, Fukagawa T, Takisawa H, Kakimoto T, Kanemaki M (2009). An auxin-based degron system for the rapid depletion of proteins in nonplant cells.. Nat Methods.

[18] Yamashita M, Hori Y, Shinomiya T, Obuse C, Tsurimoto T (1997). The efficiency and timing of initiation of replication of multiple replicons of Saccharomyces cerevisiae chromosome VI.. Genes Cells.

[19] Santos-Rosa H, Schneider R, Bannister AJ, Sherriff J, Bernstein BE (2002). Active genes are tri-methylated at K4 of histone H3.. Nature.

[20] Ng HH, Robert F, Young RA, Struhl K (2003). Targeted recruitment of Set1 histone methylase by elongating Pol II provides a localized mark and memory of recent transcriptional activity.. Mol Cell.

[21] Pokholok DK, Harbison CT, Levine S, Cole M, Hannett NM (2005). Genome-wide map of nucleosome acetylation and methylation in yeast.. Cell.

[22] Haruki H, Nishikawa J, Laemmli UK (2008). The anchor-away technique: rapid, conditional establishment of yeast mutant phenotypes.. Mol Cell.

[23] Strahl-Bolsinger S, Hecht A, Luo K, Grunstein M (1997). SIR2 and SIR4 interactions differ in core and extended telomeric heterochromatin in yeast.. Genes Dev.

[24] Rusche LN, Kirchmaier AL, Rine J (2002). Ordered nucleation and spreading of silenced chromatin in Saccharomyces cerevisiae.. Mol Biol Cell.

[25] Radman-Livaja M, Verzijlbergen KF, Weiner A, van Welsem T, Friedman N (2011). Patterns and mechanisms of ancestral histone protein inheritance in budding yeast.. PLoS Biol.

[26] Kimura A, Umehara T, Horikoshi M (2002). Chromosomal gradient of histone acetylation established by Sas2p and Sir2p functions as a shield against gene silencing.. Nat Genet.

[27] Santos-Rosa H, Bannister AJ, Dehe PM, Geli V, Kouzarides T (2004). Methylation of H3 lysine 4 at euchromatin promotes Sir3p association with heterochromatin.. J Biol Chem.

[28] Verzijlbergen KF, Faber AW, Stulemeijer IJ, van Leeuwen F (2009). Multiple histone modifications in euchromatin promote heterochromatin formation by redundant mechanisms in Saccharomyces cerevisiae.. BMC Mol Biol.

[29] Fischle W, Wang Y, Allis CD (2003). Histone and chromatin cross-talk.. Curr Opin Cell Biol.

[30] Lachner M, O'Sullivan RJ, Jenuwein T (2003). An epigenetic road map for histone lysine methylation.. J Cell Sci.

[31] Nakayama J, Rice JC, Strahl BD, Allis CD, Grewal SI (2001). Role of histone H3 lysine 9 methylation in epigenetic control of heterochromatin assembly.. Science.

[32] Gietz RD, Sugino A (1988). New yeast-Escherichia coli shuttle vectors constructed with in vitro mutagenized yeast genes lacking six-base pair restriction sites.. Gene.

[33] Thomas BJ, Rothstein R (1989). Elevated recombination rates in transcriptionally active DNA.. Cell.

[34] Longtine MS, McKenzie A, Demarini DJ, Shah NG, Wach A (1998). Additional modules for versatile and economical PCR-based gene deletion and modification in Saccharomyces cerevisiae.. Yeast.

[35] Goldstein AL, McCusker JH (1999). Three new dominant drug resistance cassettes for gene disruption in Saccharomyces cerevisiae.. Yeast.

[36] Tanaka T, Knapp D, Nasmyth K (1997). Loading of an Mcm protein onto DNA replication origins is regulated by Cdc6p and CDKs.. Cell.

[37] Lindroos HB, Strom L, Itoh T, Katou Y, Shirahige K (2006). Chromosomal association of the Smc5/6 complex reveals that it functions in differently regulated pathways.. Mol Cell.

[38] Xu F, Zhang Q, Zhang K, Xie W, Grunstein M (2007). Sir2 deacetylates histone H3 lysine 56 to regulate telomeric heterochromatin structure in yeast.. Mol Cell.

