# Supplementary figures and images for: The Inheritance of Histone Modifications Depends upon the Location in the Chromosome in Saccharomyces cerevisiae
Source: PLoS One. 2011 Dec 21;6(12):e28980. doi: 10.1371/journal.pone.0028980 (PMC3244422; doi:10.1371/journal.pone.0028980)

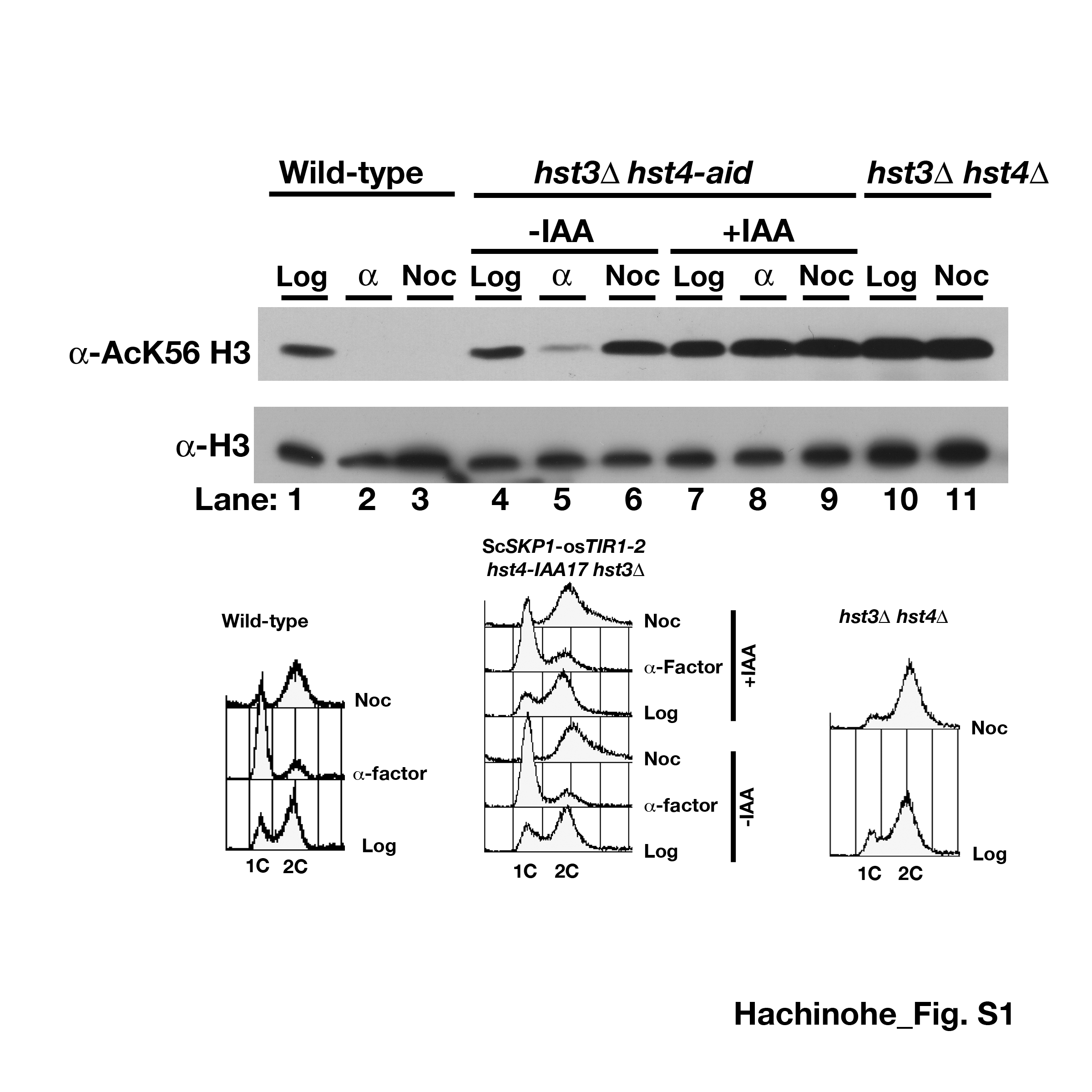

Supplement: Figure S1 — AID system prevents the deacetylation of histone H3-K56 by promoting Hst4-aid degradation in hst3Δ strain. Cell extracts were prepared from each strain (Wild type (W303-1a), hst3Δ hst4-aid (HMY837) and hst3Δ hst4Δ (HMY278)) arrested at each cell cycle stage (α-arrest (G1), Nocodazole-arrest (G2/M), and Log phase). hst3Δ hst4-aid cells were additionally treated with or without IAA, The histone H3-K56 acetylation and the total amount of histone H3 were analyzed by immunoblot using antibody to AcK56 H3 and histone H3, respectively. Cell cycle arrest was monitored by FACS analysis. We confirmed that H3-K56 remained acetylated at G1 phase in hst3Δ hst4-aid cell with treatment of IAA, but H3-K56 had been deacetylated without IAA (Lanes 5 and 8). (TIF) [file pone.0028980.s001.tif]

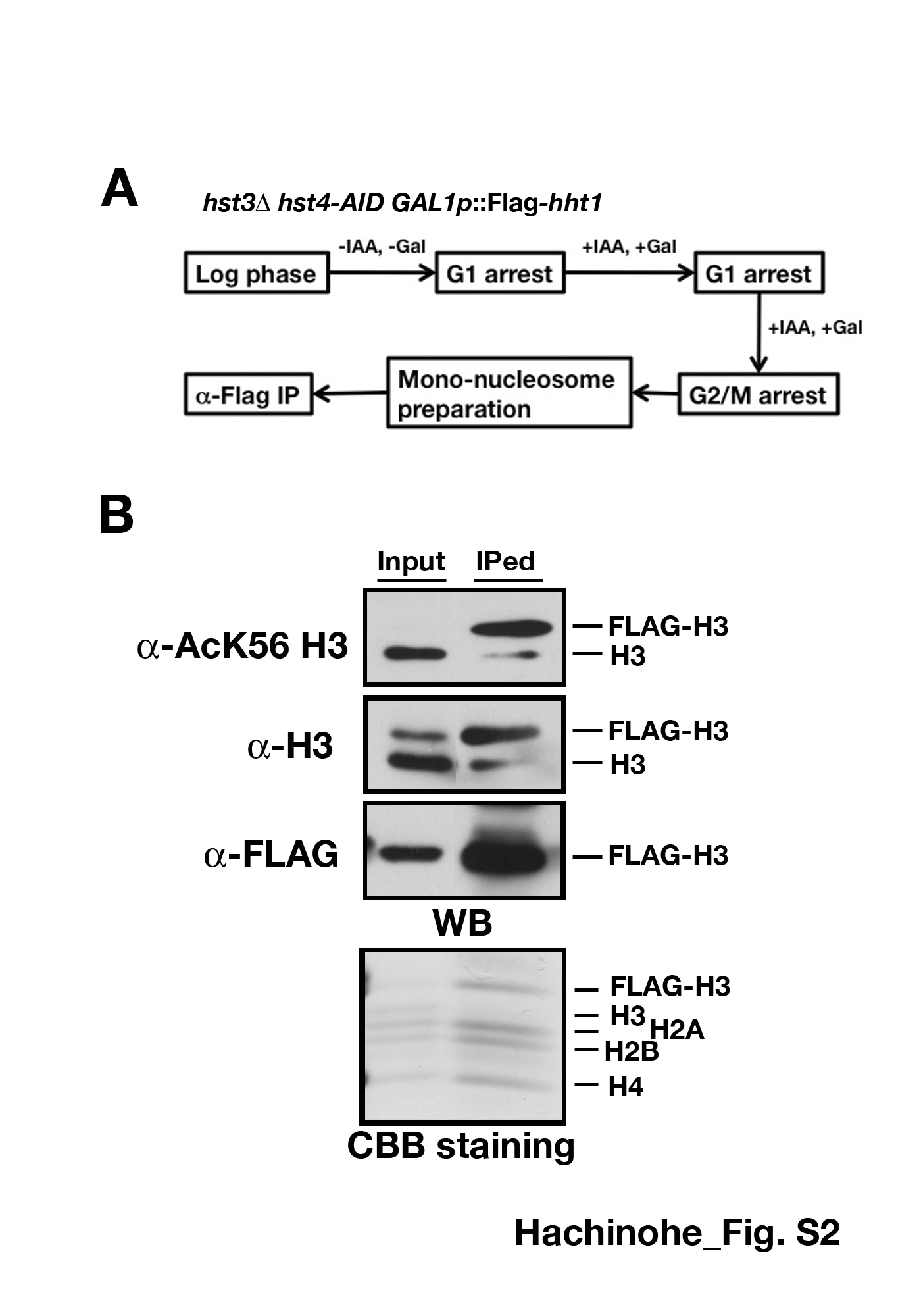

Supplement: Figure S2 — Histone H3-H4 partition in newly deposited nucleosome is composed of newly synthesized nucleosome using N-terminal Flag-tagging histone H3. (A) The experimental procedure of isolation of newly deposited nucleosome using N-terminal Flag-tagging histone H3 (Flag-H3). (B) Immunoprecipitated newly deposited nucleosome containing Flag-H3 was separated by SDS-PAGE, and stained by CBB staining to visualize histone proteins, or transferred to a nitrocellulose membrane. Western blotting analysis with Flag-H3, K56-acetylated H3 and whole histone H3, respectively, is shown. (TIF) [file pone.0028980.s002.tif]

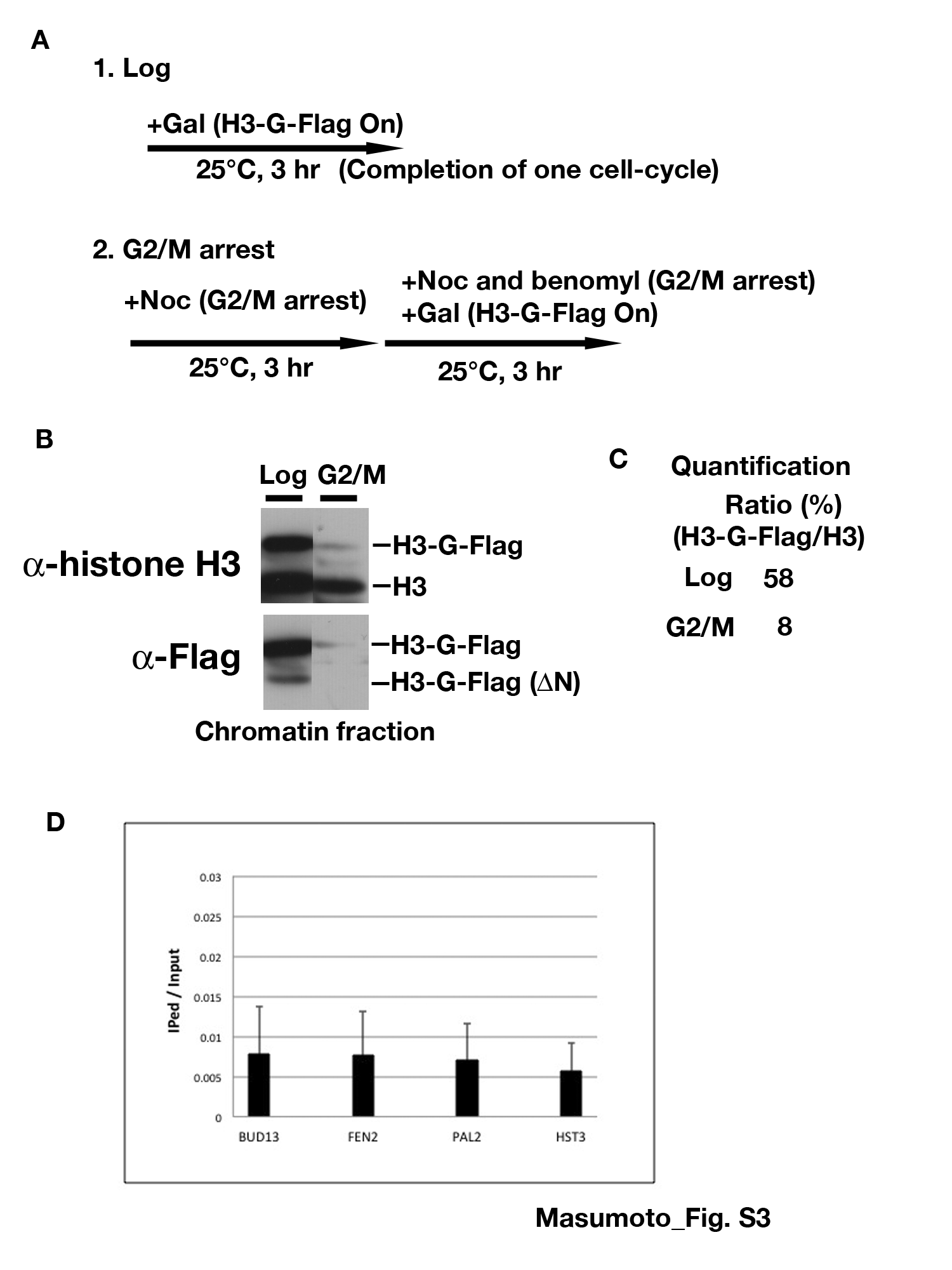

Supplement: Figure S3 — Histone exchange with free histone H3-G-Flag and histone H3 in chromatin at G2/M-arrested cell. (A) A scheme of procedure of induction of histone H3-G-Flag in G2/M arrest cell. HMY616 cells were arrested at G2/M phase in YPR medium containing nocodazole at a final concentration of 10 µg/ml, and then further treated with nocodazole and benomyl at final concentrations of 5 µg/ml and 20 µg/ml, respectively, in the presence of 2% galactose at 25°C for 3 hr [2]. (B) Chromatin was isolated from cells and analyzed by immunoblot using antibody to Flag epitope and histone H3, respectively. (C) Amounts of histone H3-G-Flag and histone H3 in immunoblot using anti-histone H3 antibody (B) were quantified by Image J software (NIH, USA). (D) ChIP-quantitative PCR analysis using anti-Flag antibody for association of free histone H3-G-Flag at different gene loci. Chromatin was prepared from G2/M arrested HMY616 cells. The graphs represent the average and standard deviation of two independent experiments. (TIF) [file pone.0028980.s003.tif]

chr1

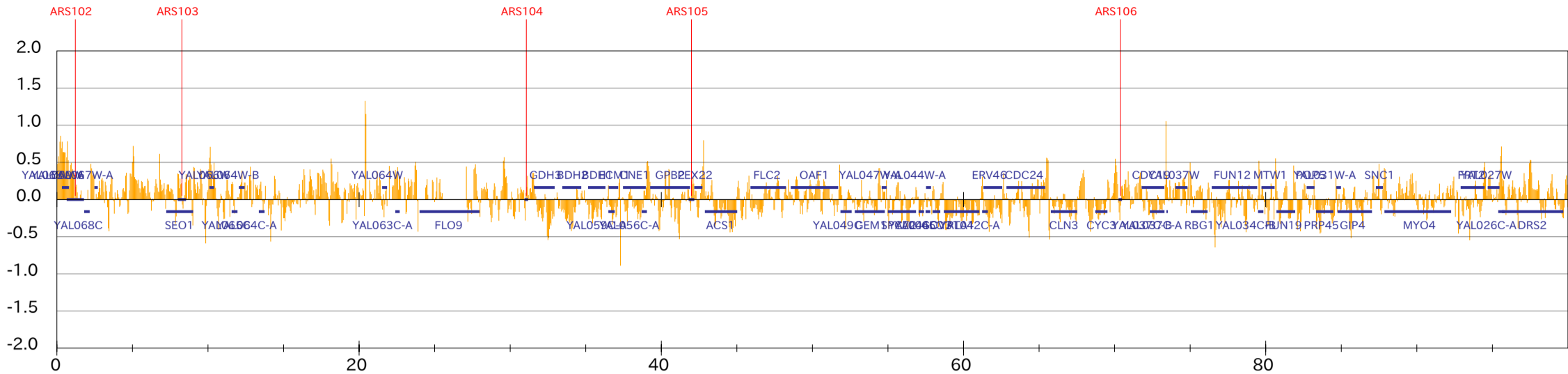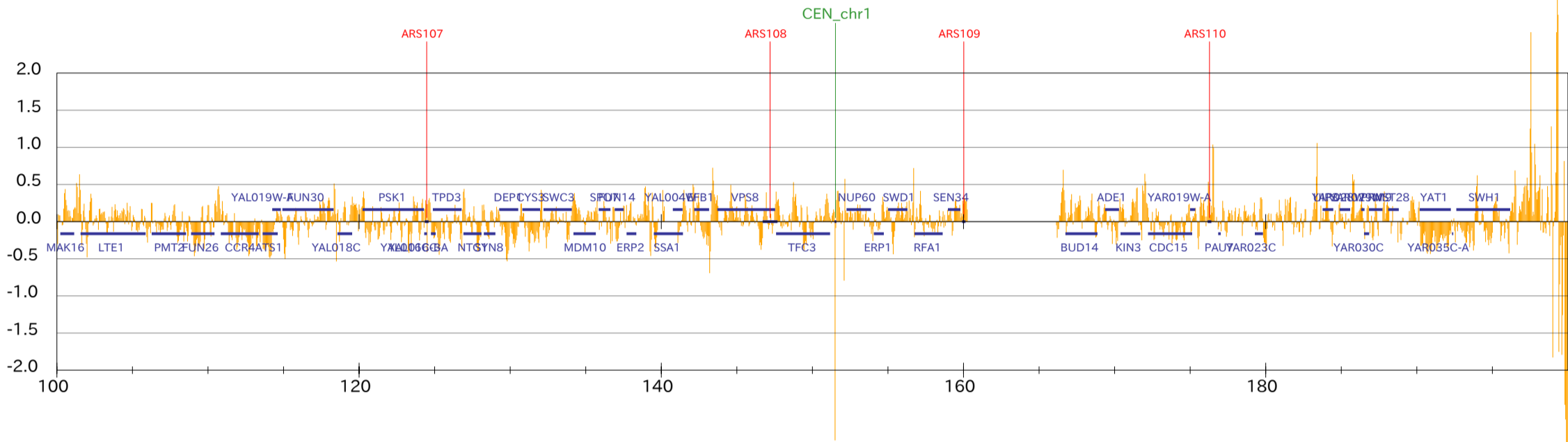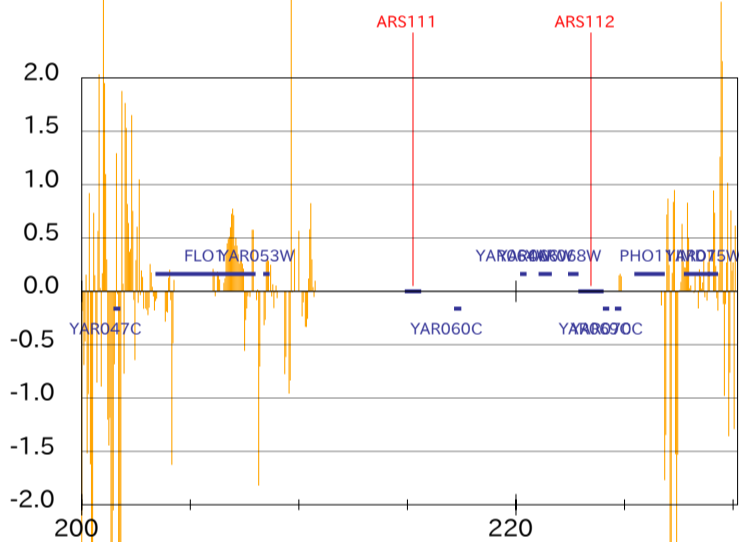

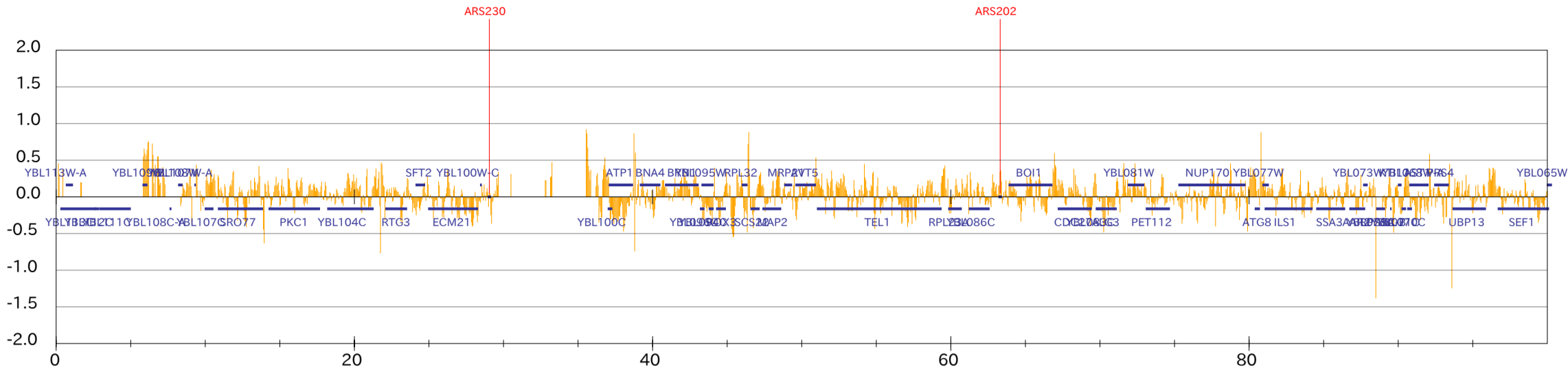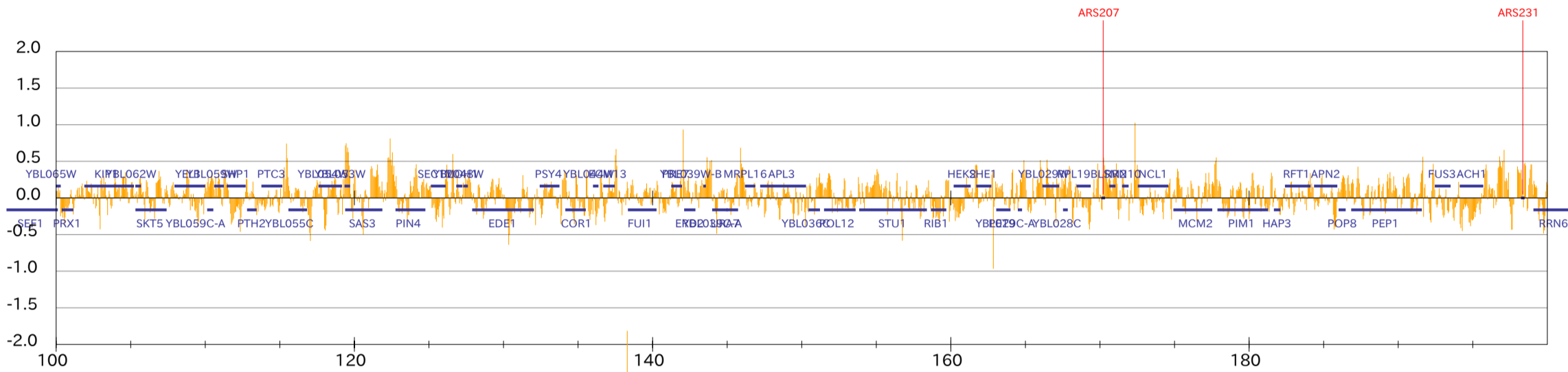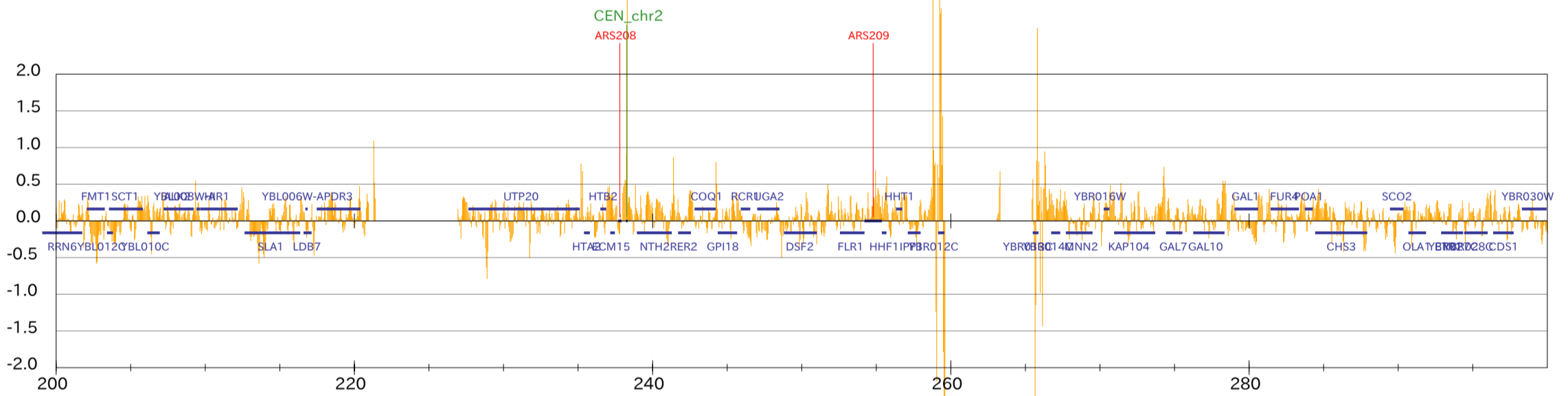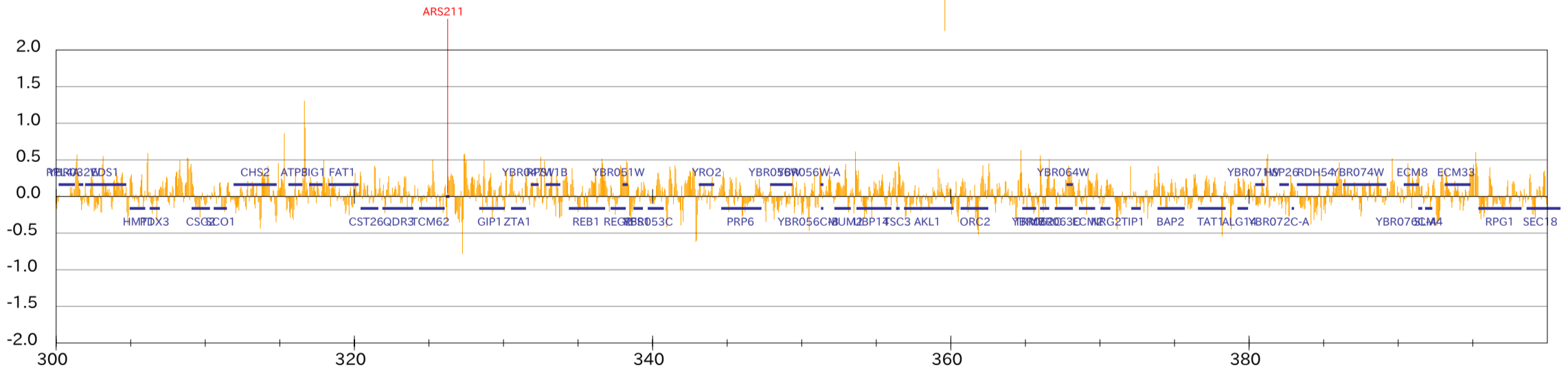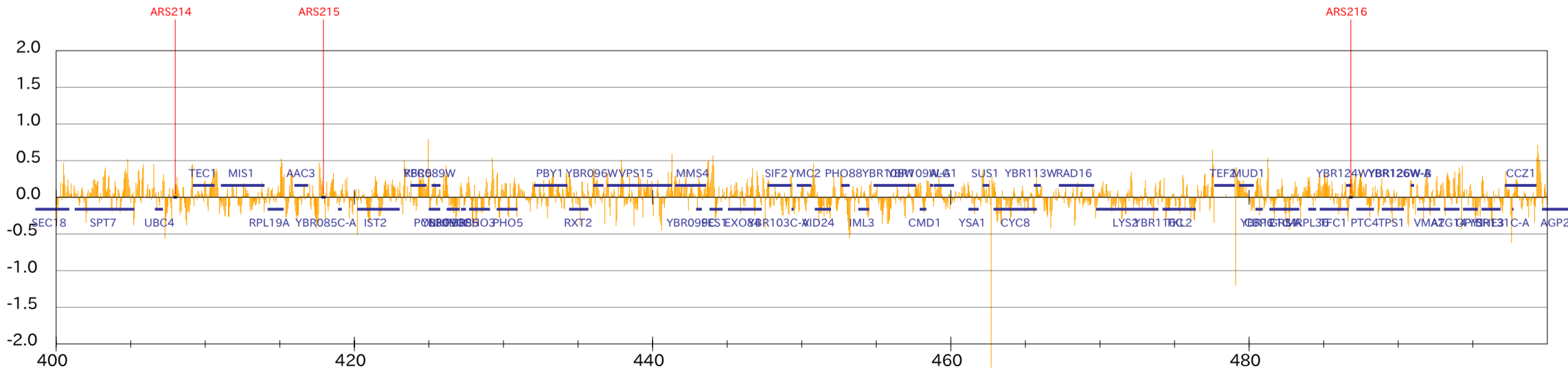

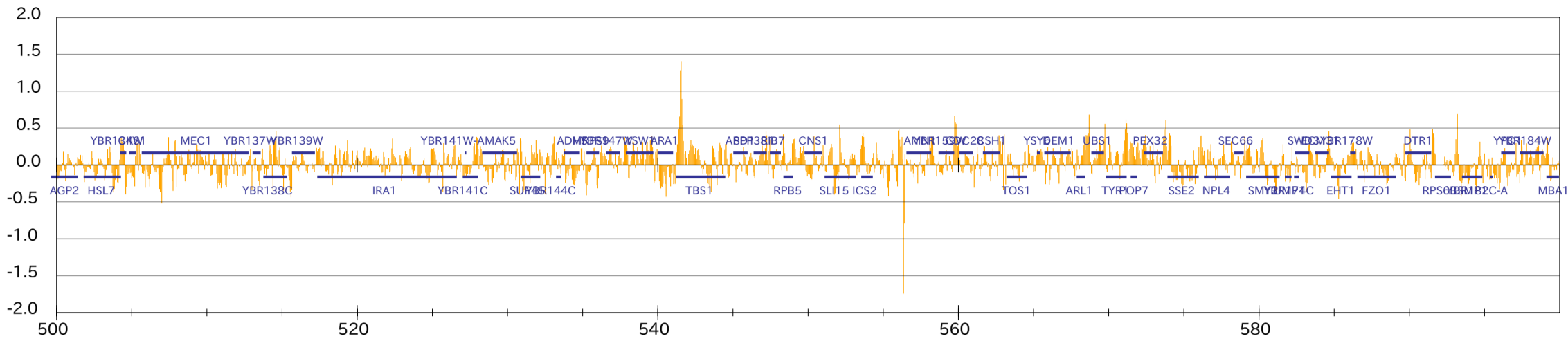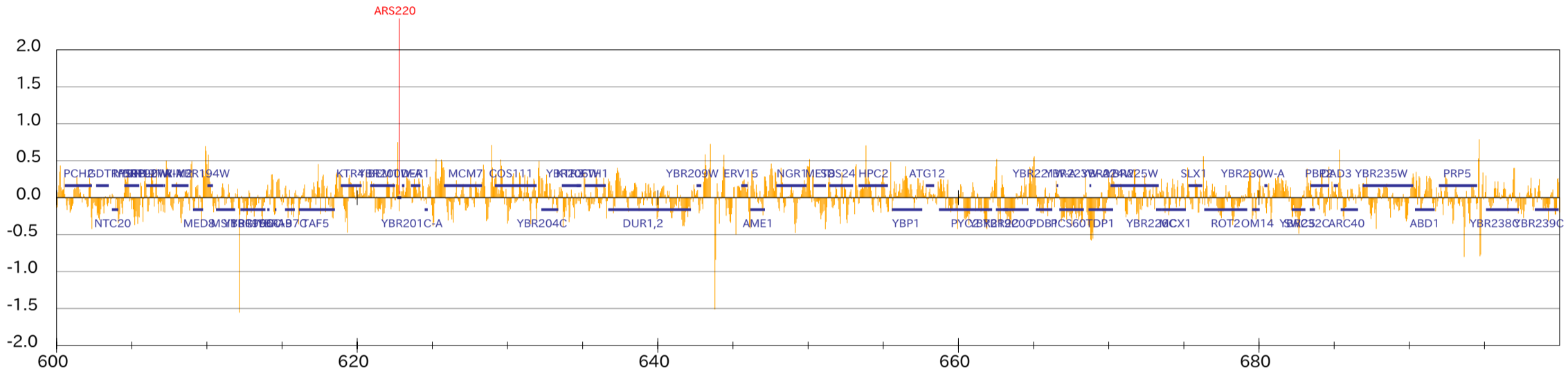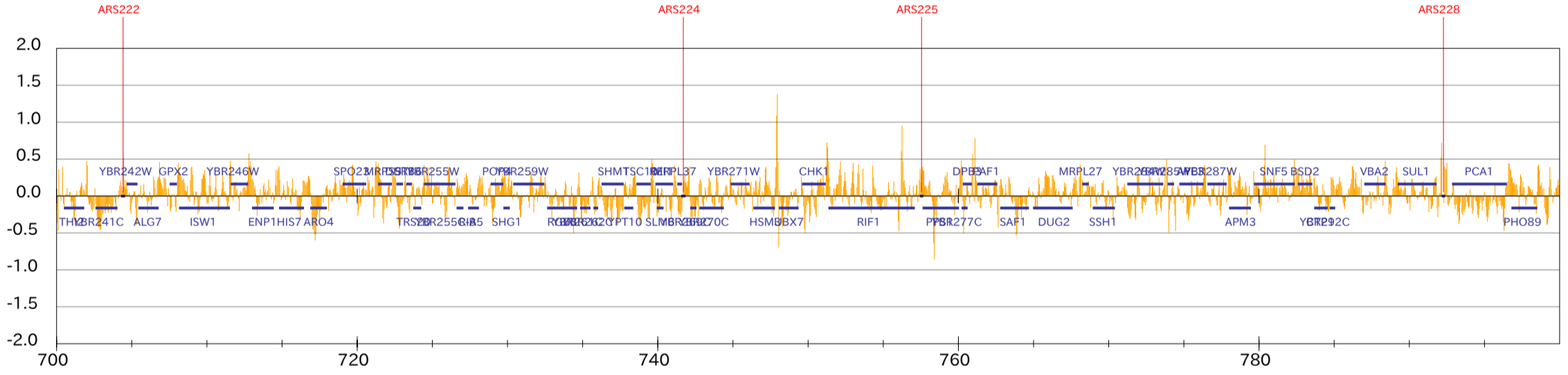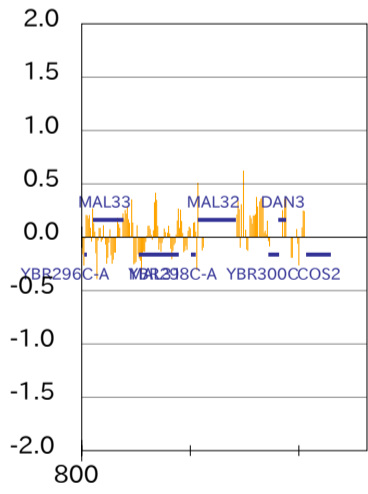



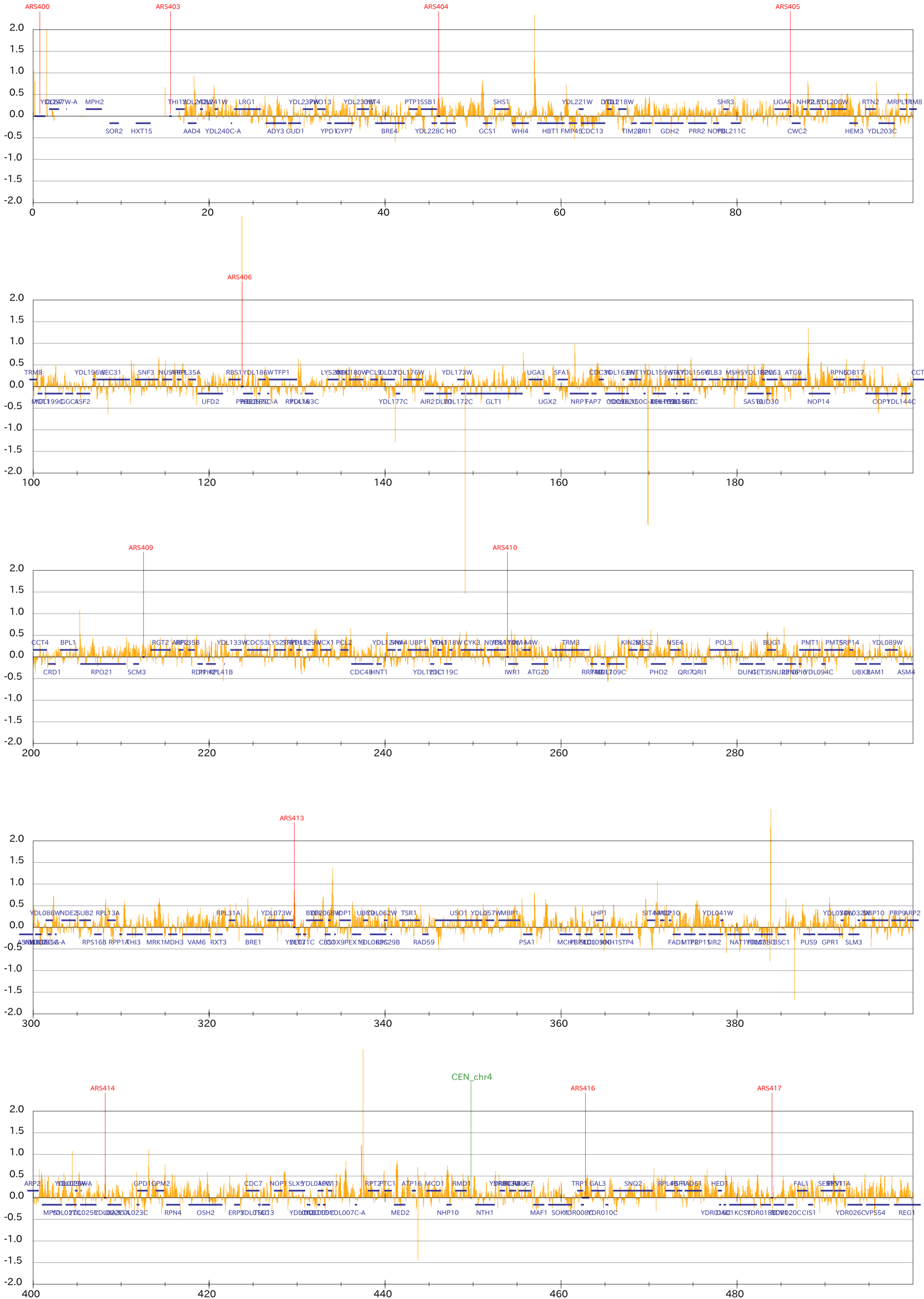





chr4\_4

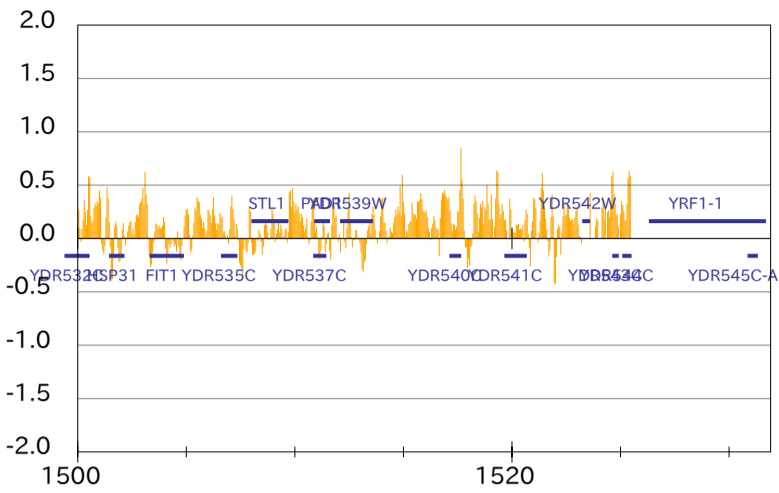



chr5\_2

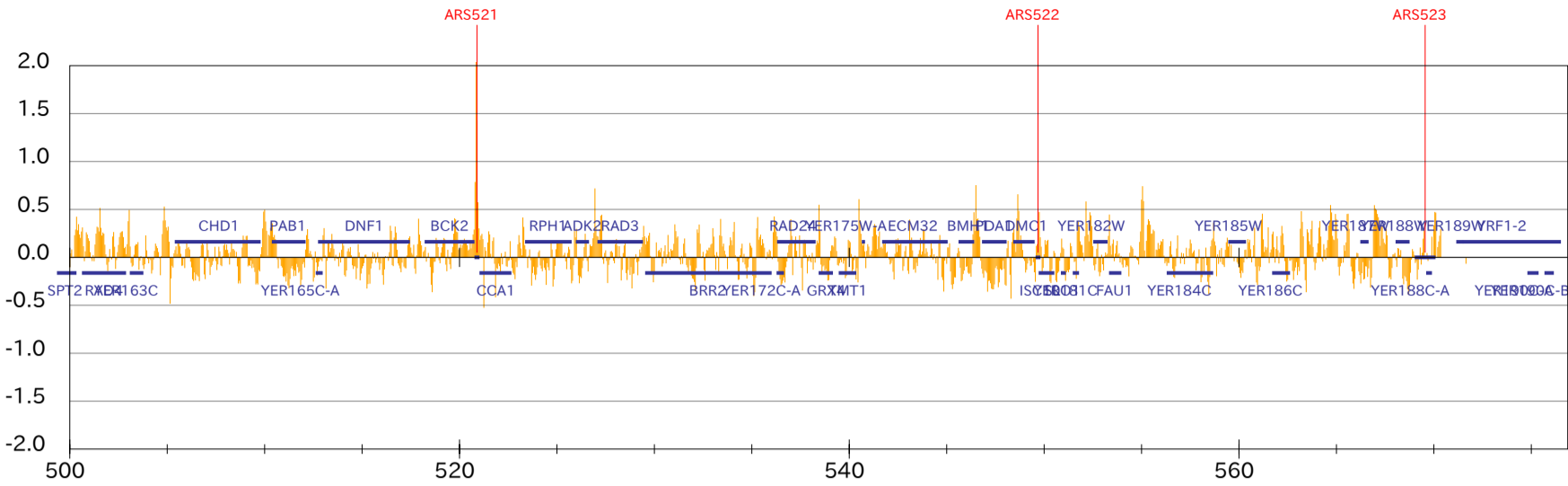

chr6

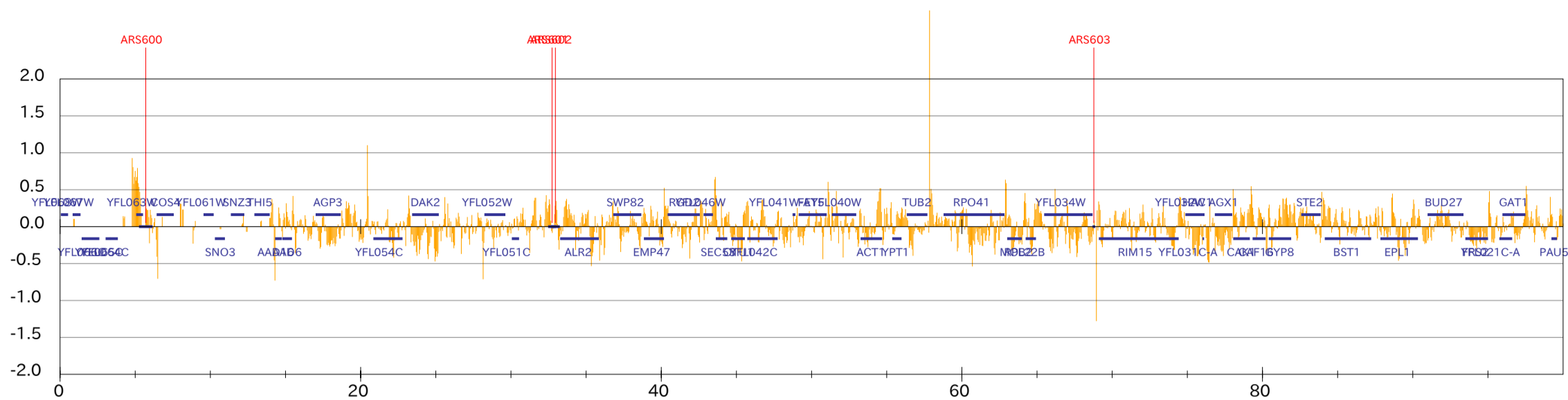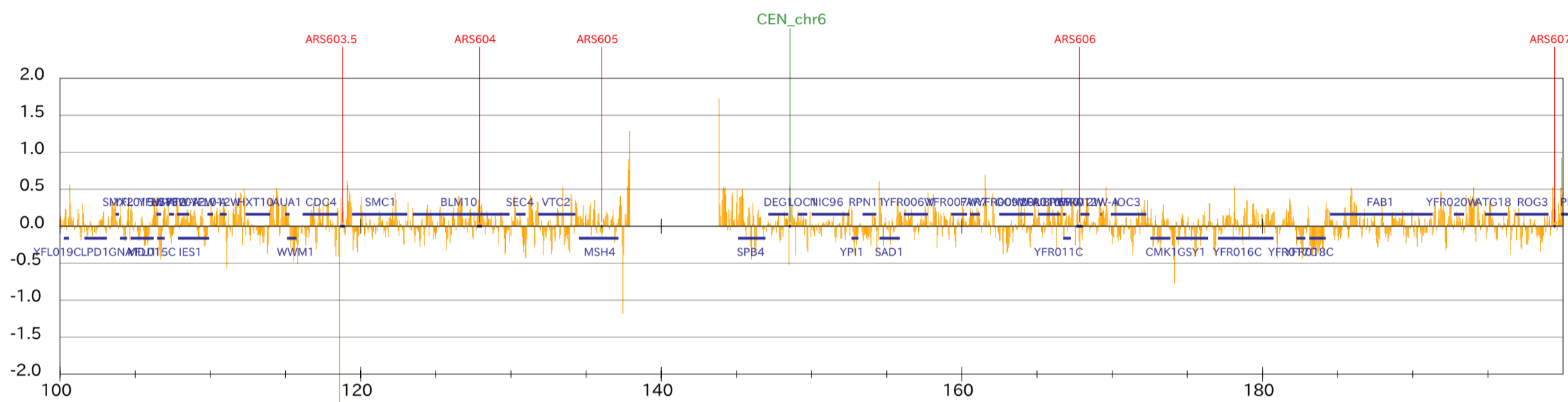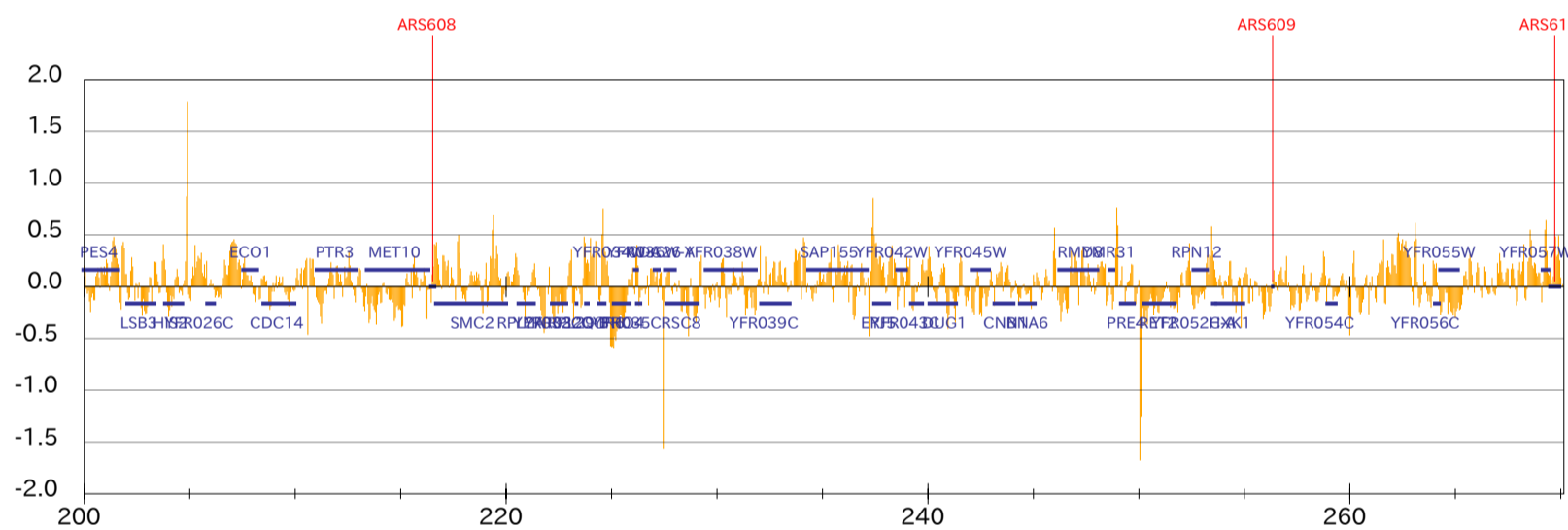





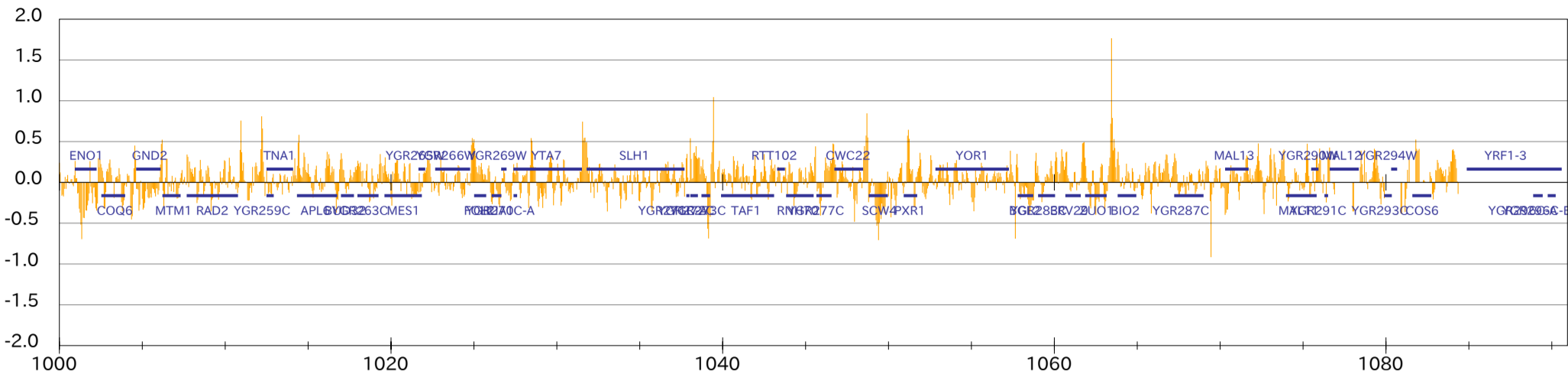

chr8\_1

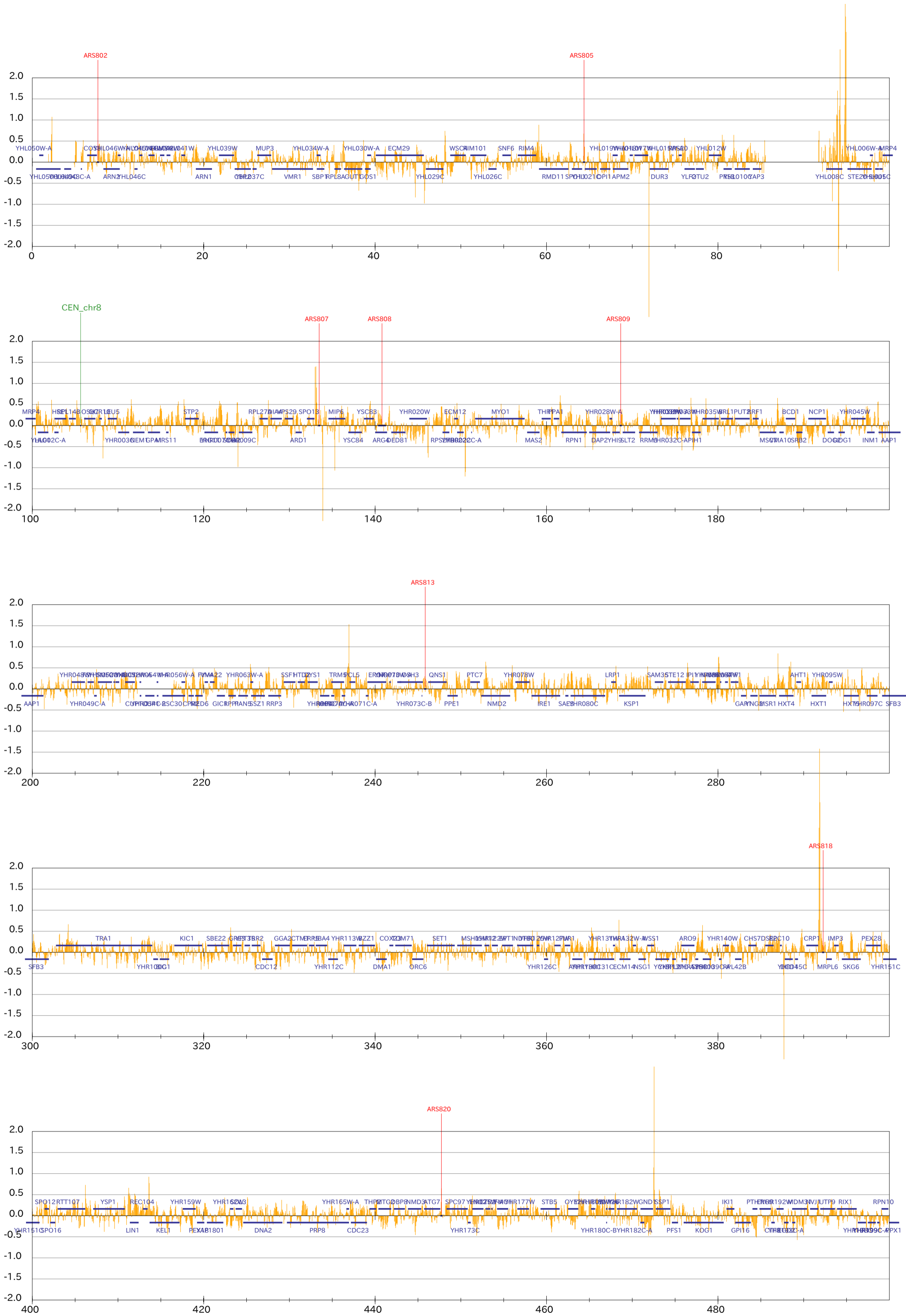

chr8\_2

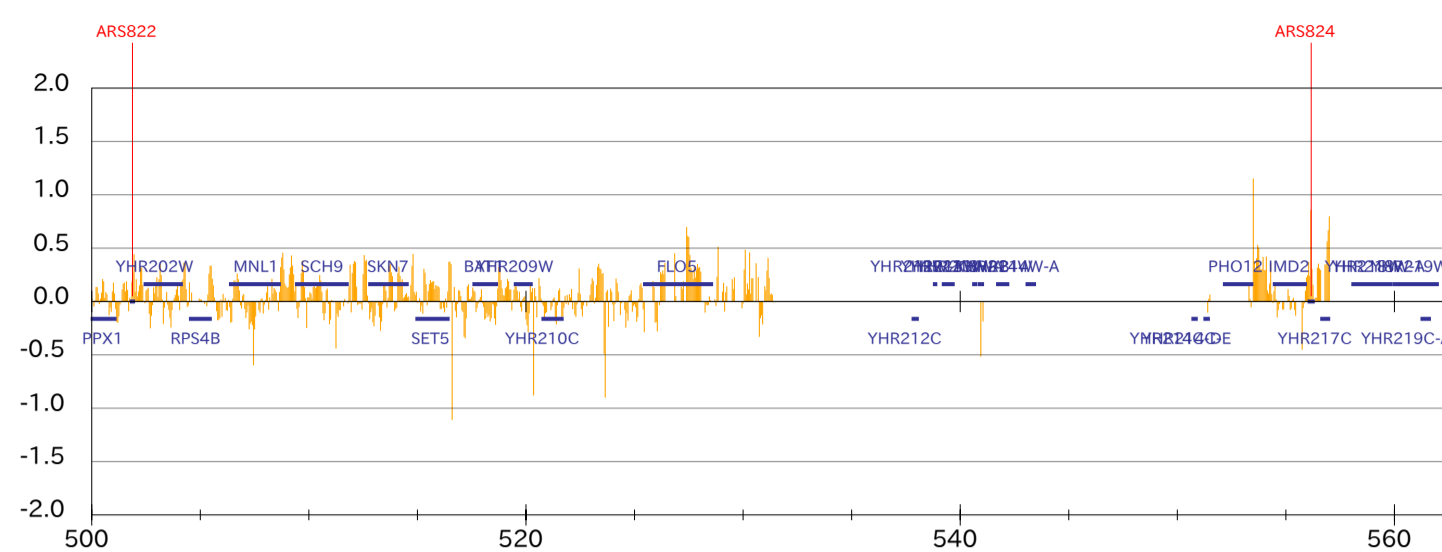





chr10\_2

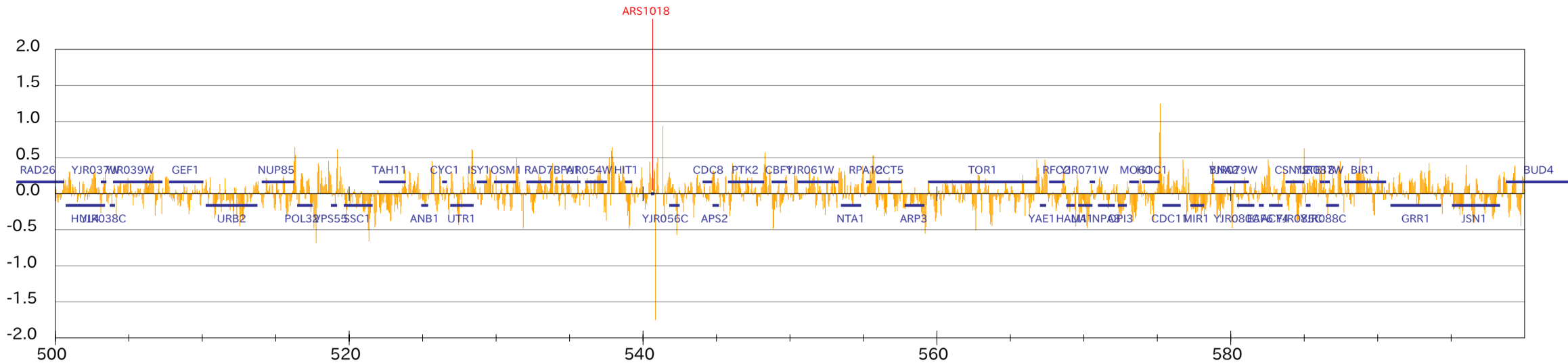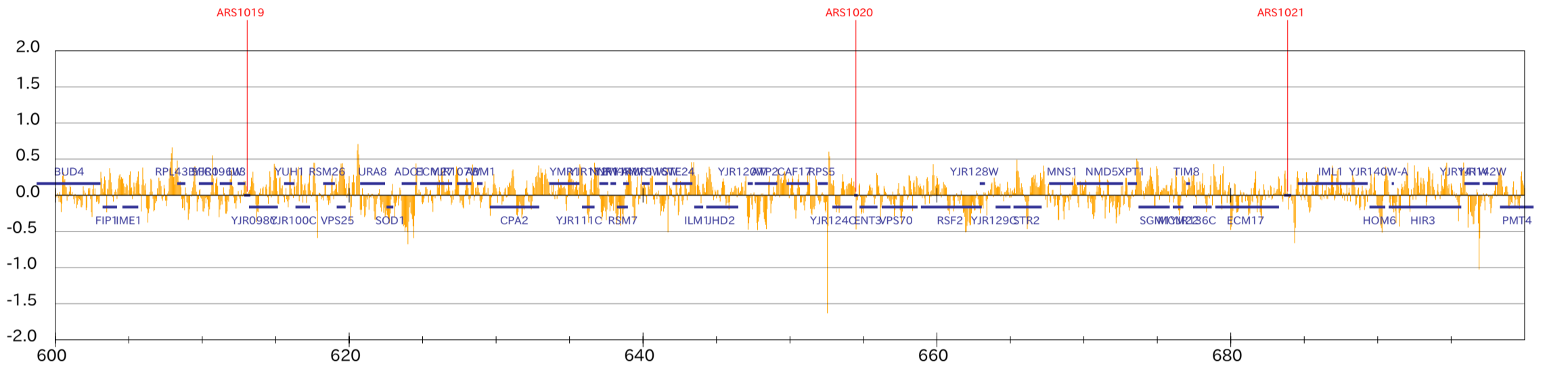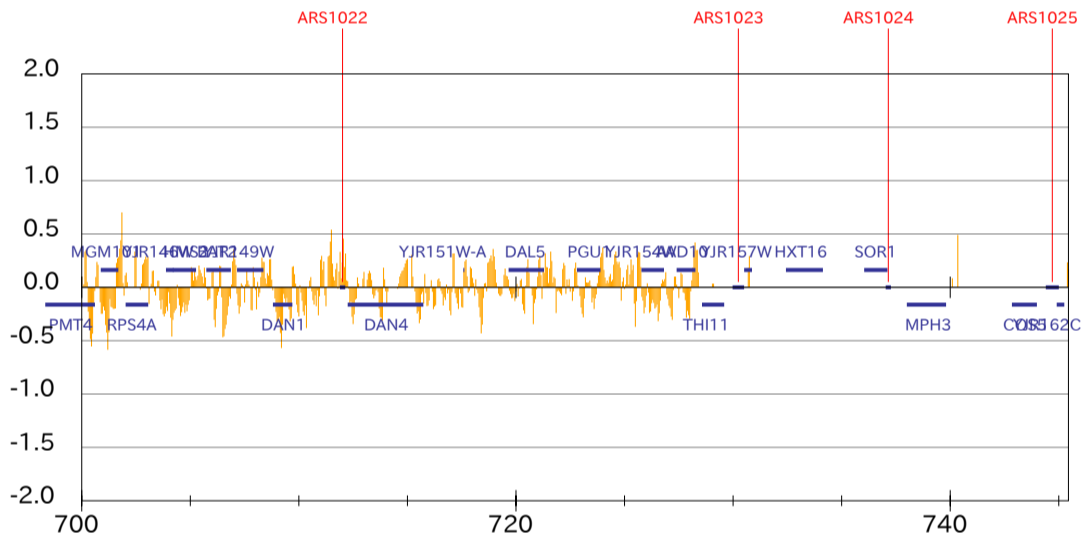

chr11\_1

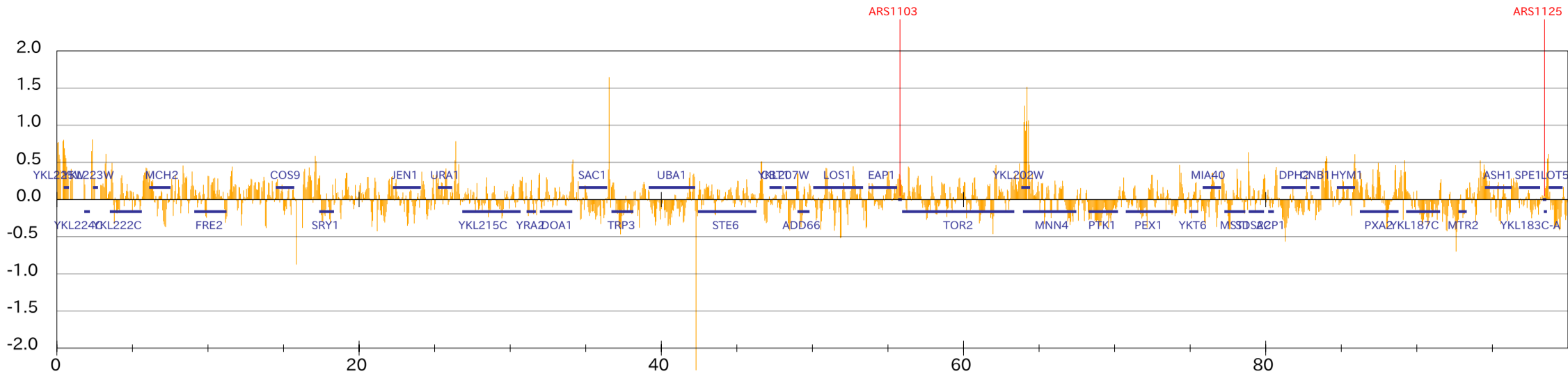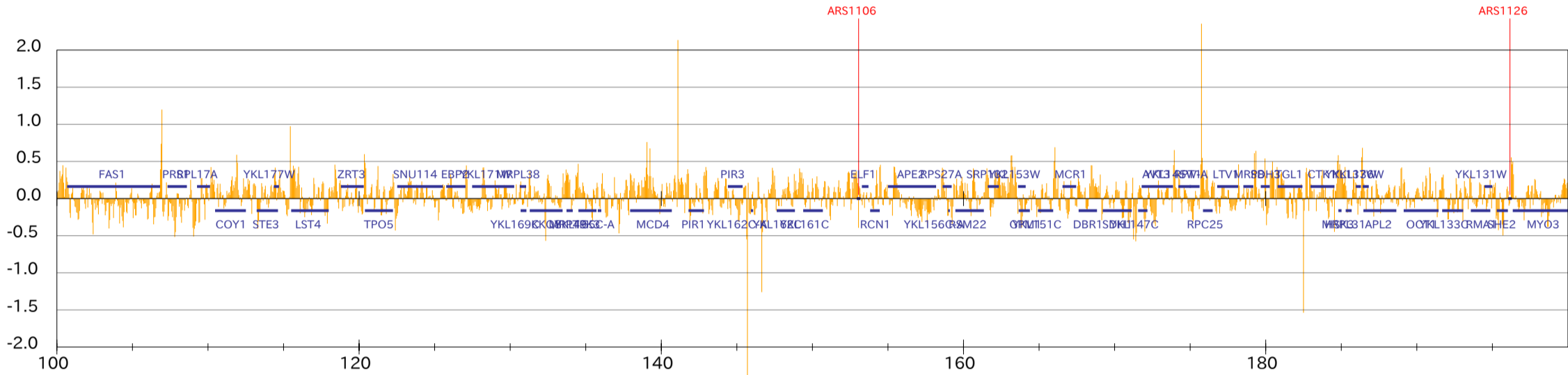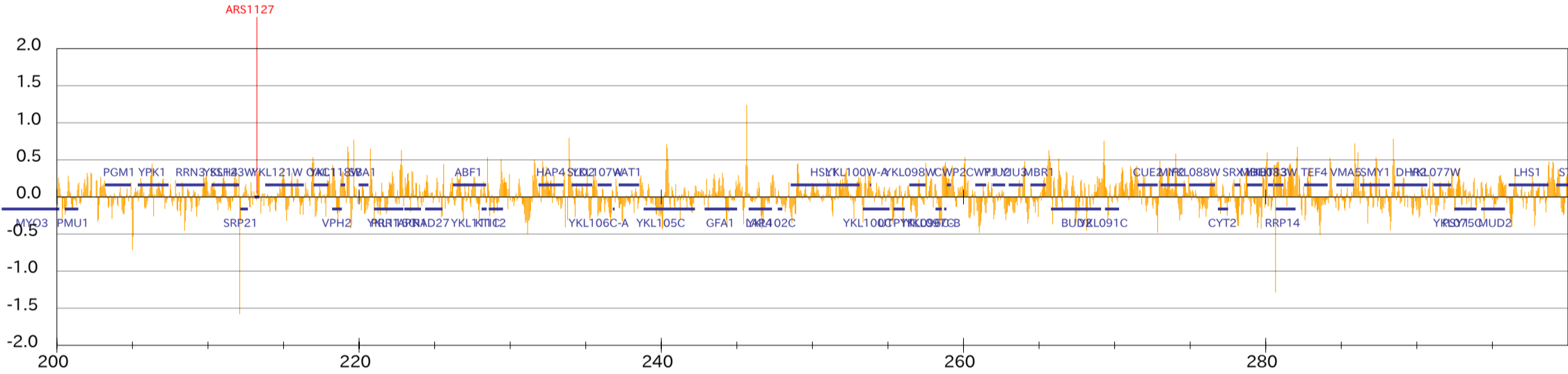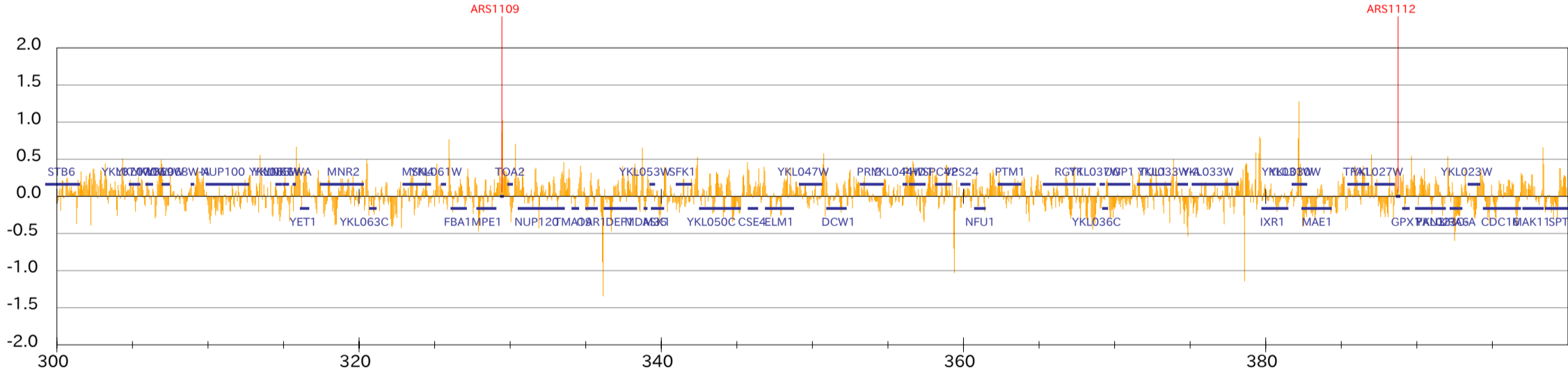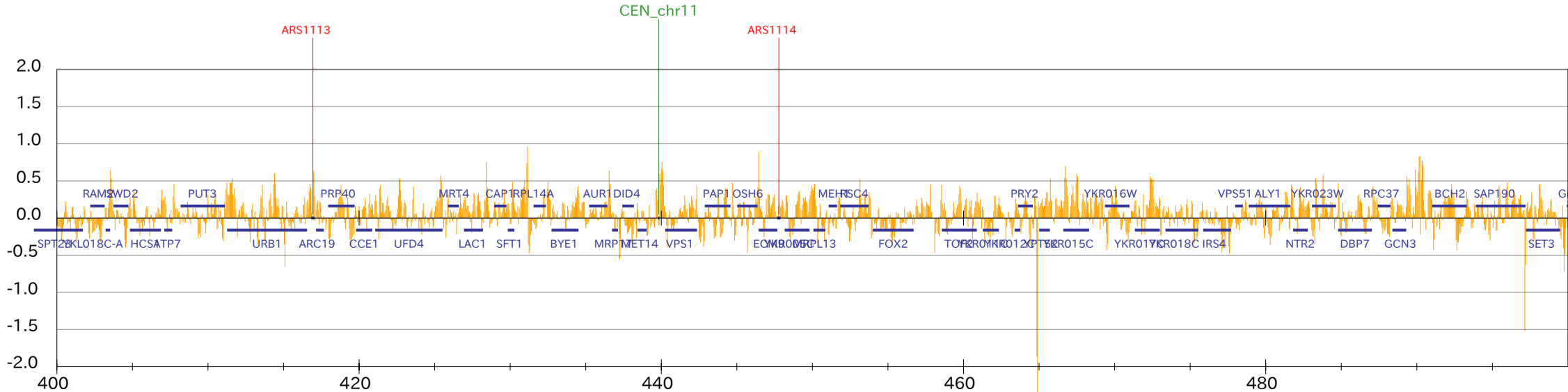

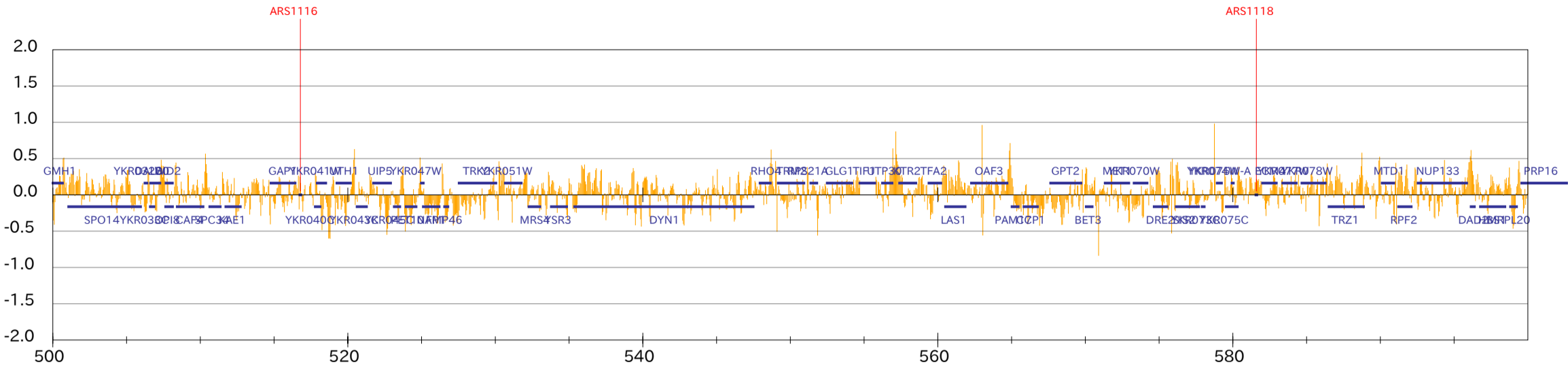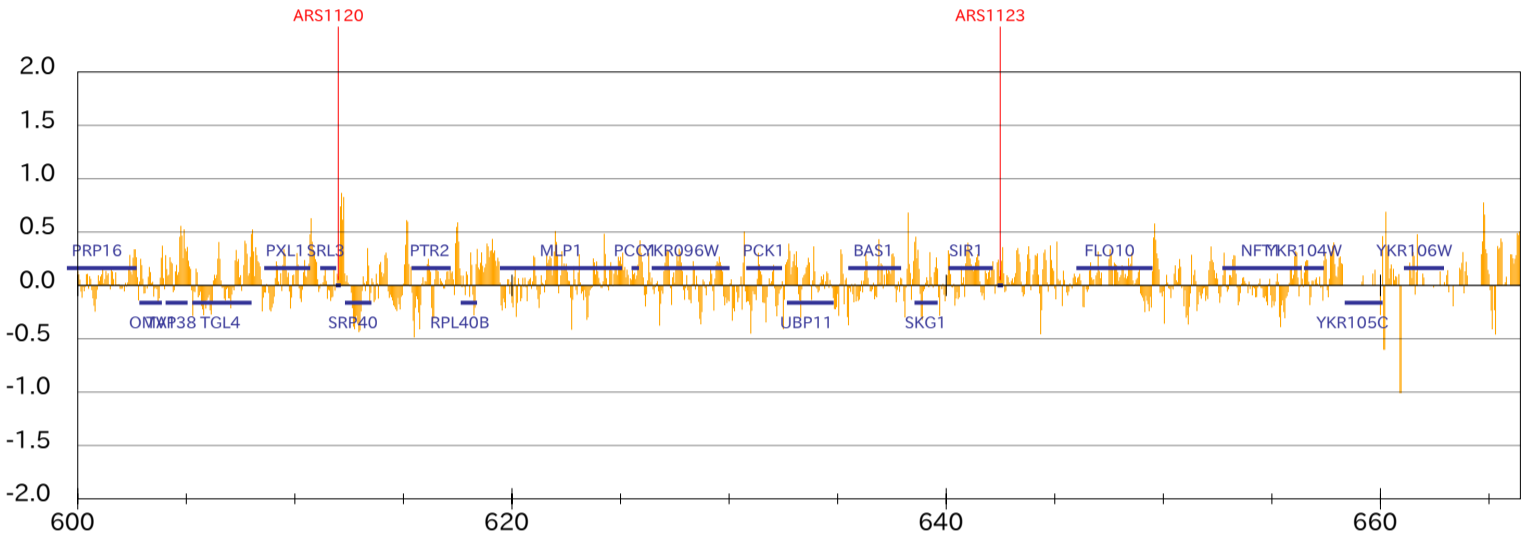







chr13\_1

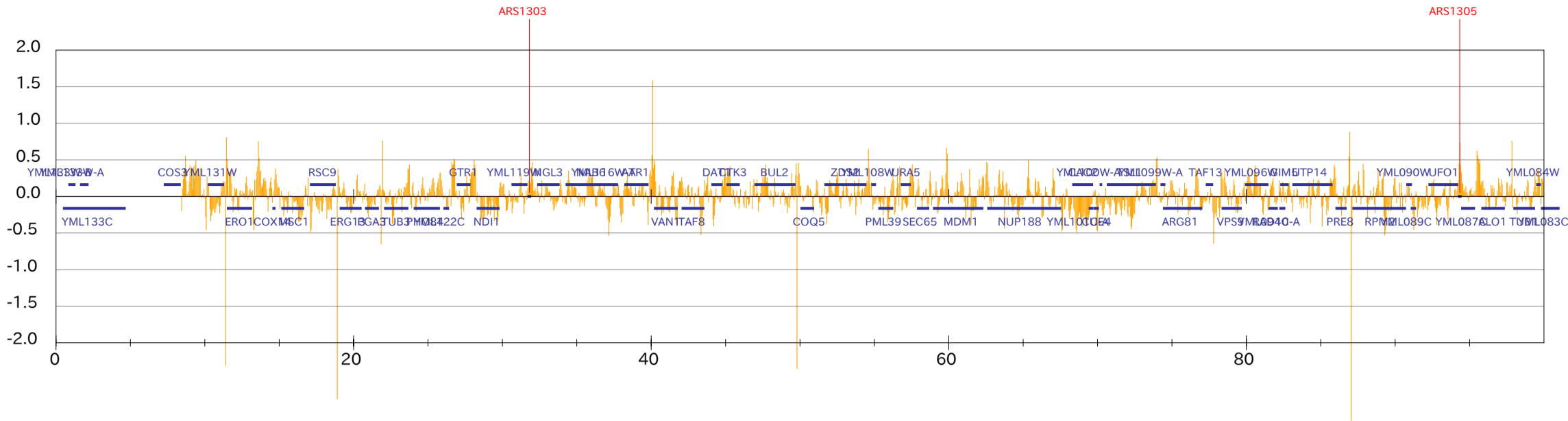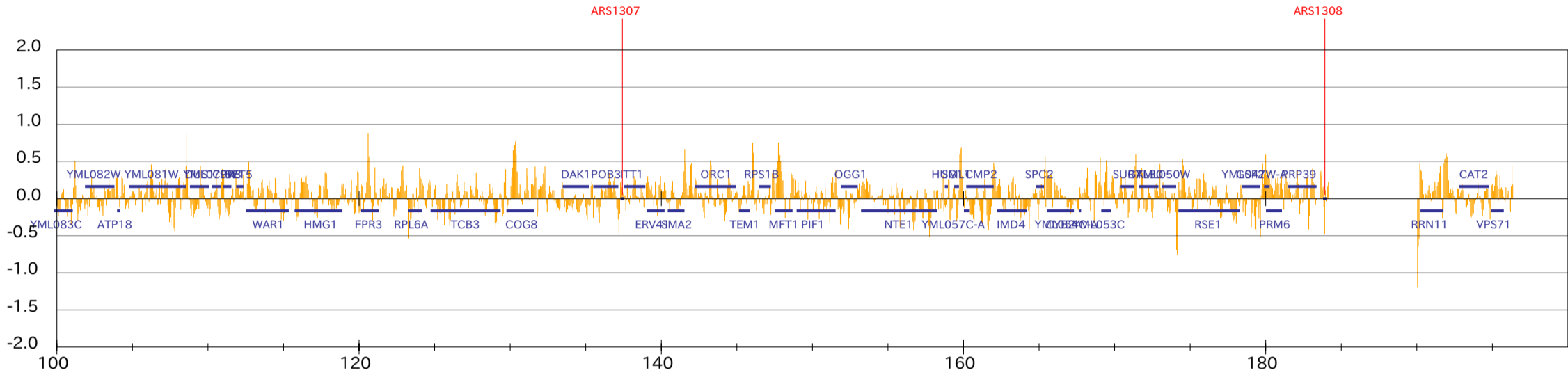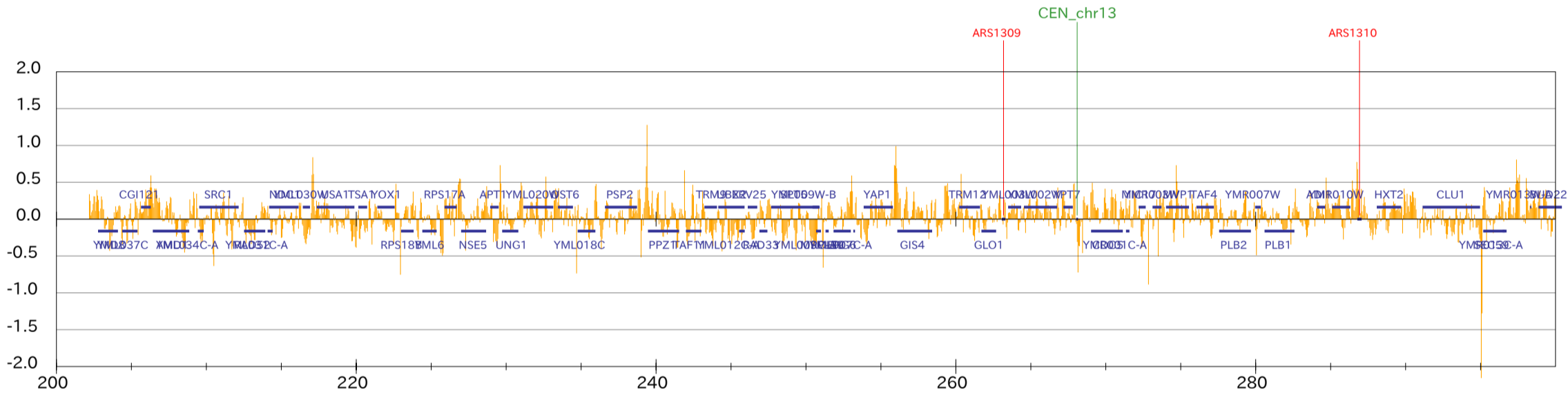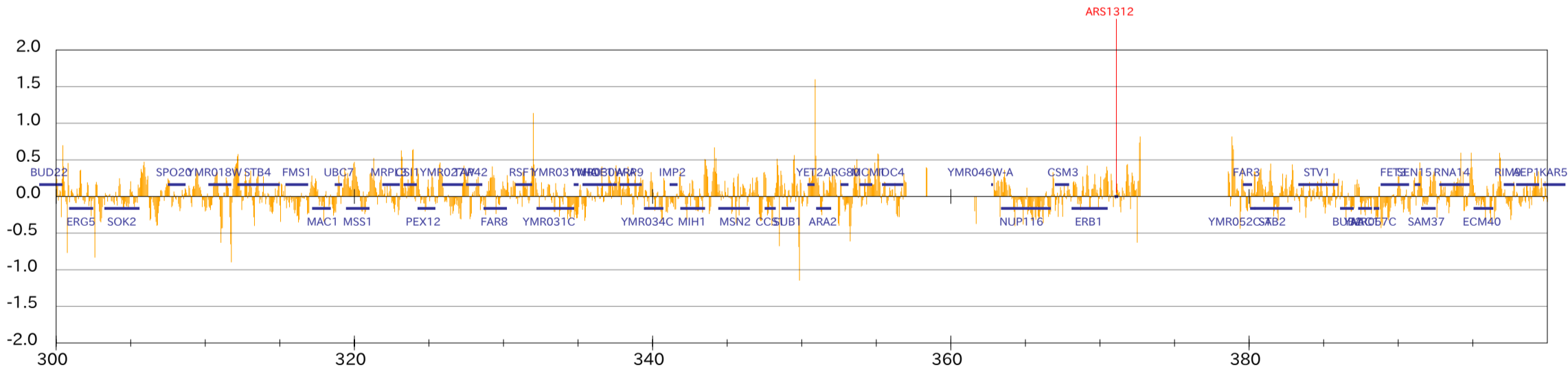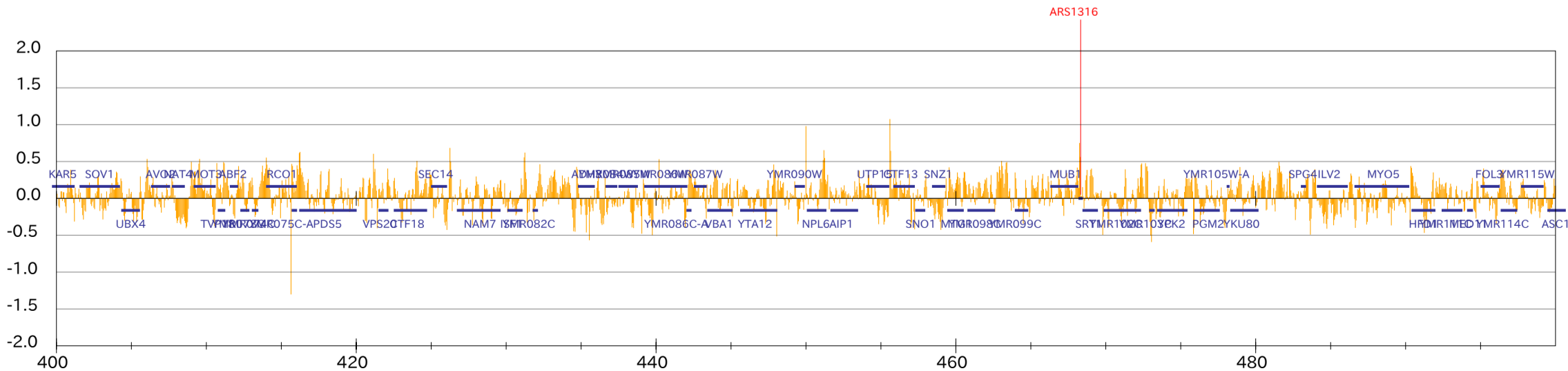



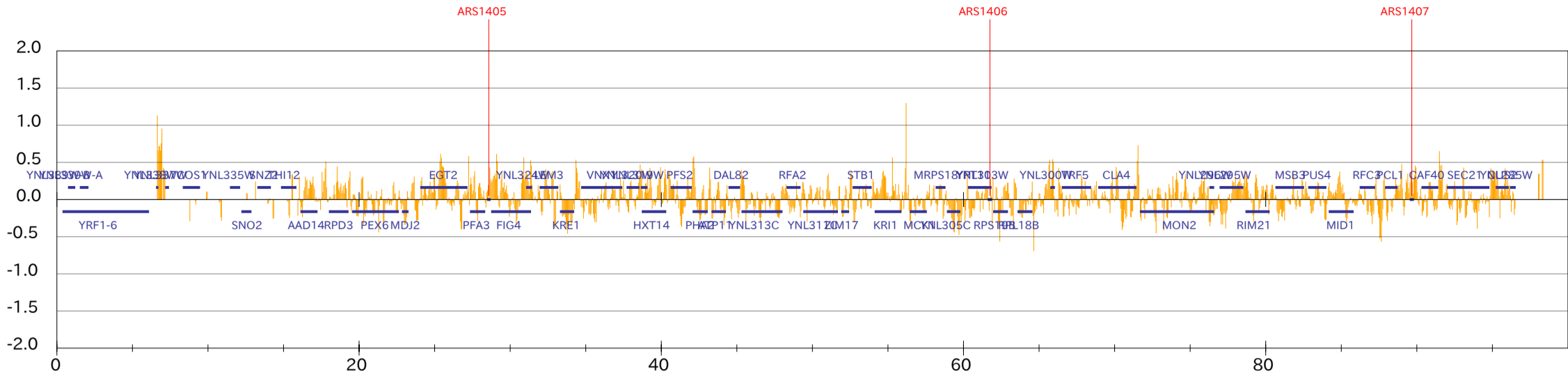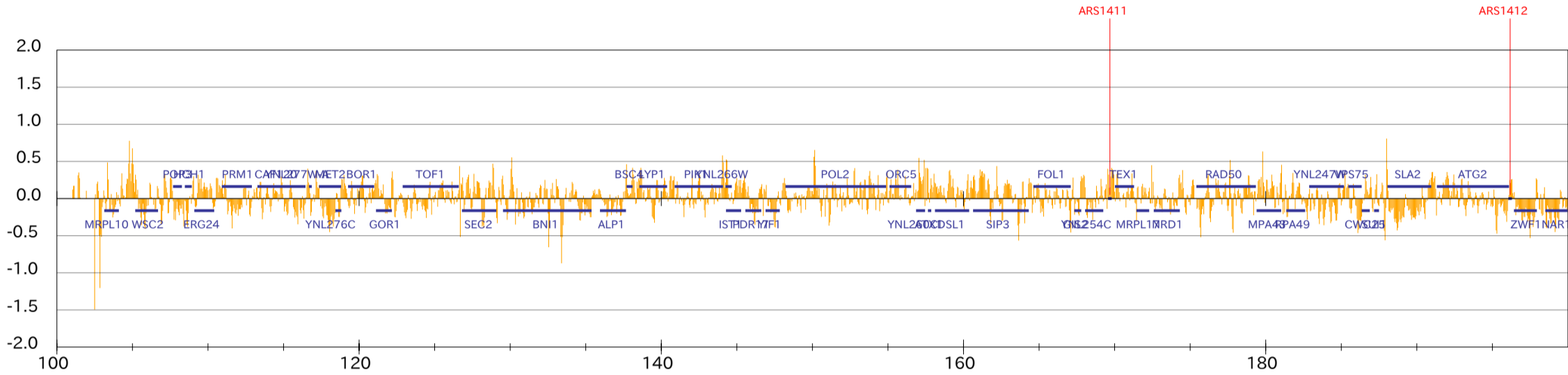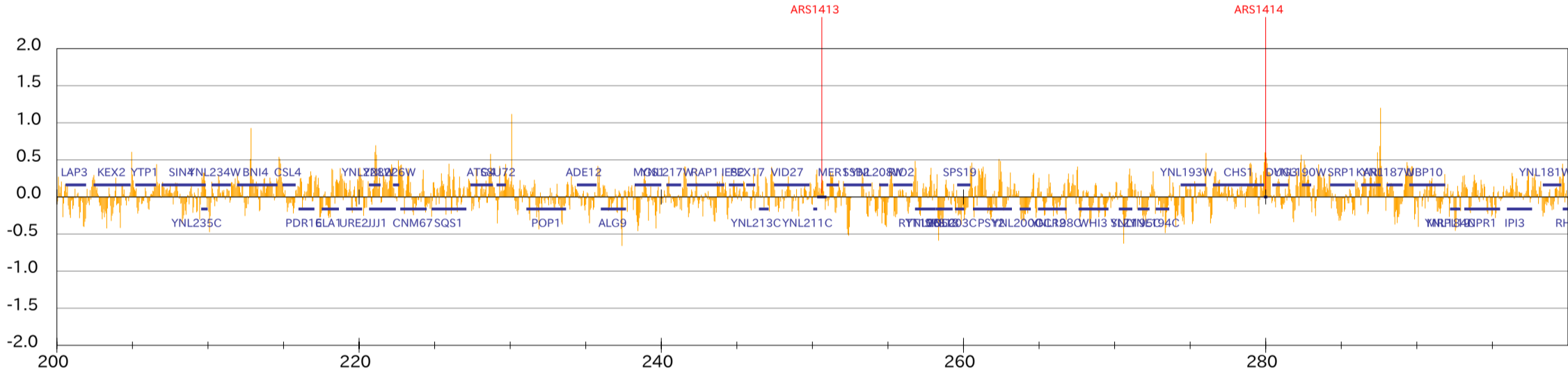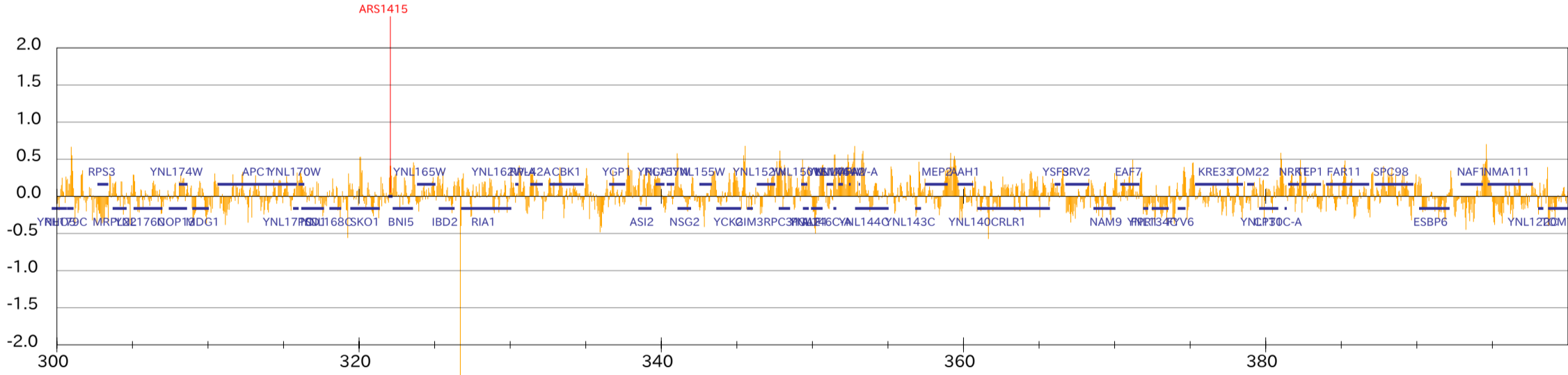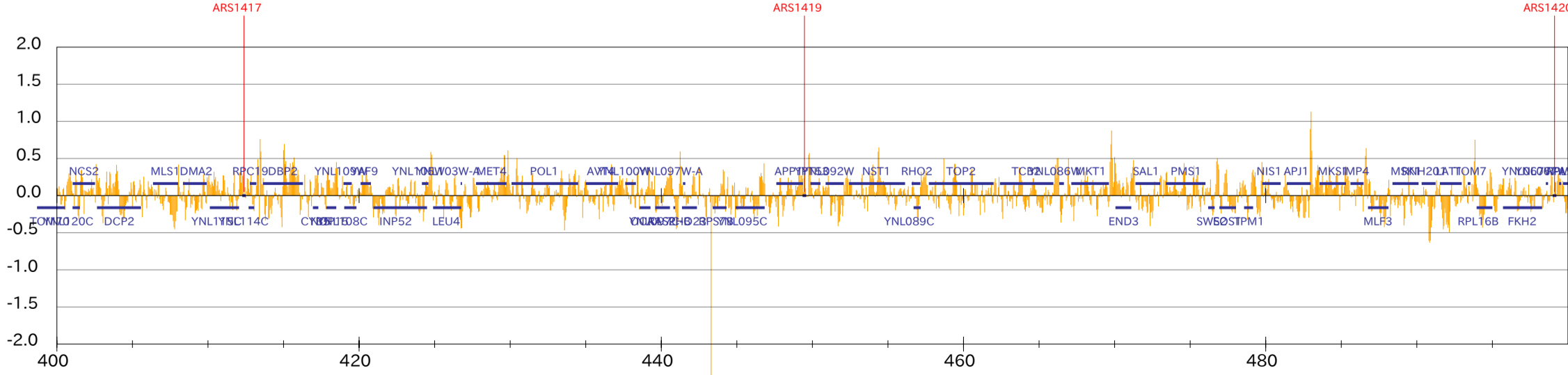



chr15\_1

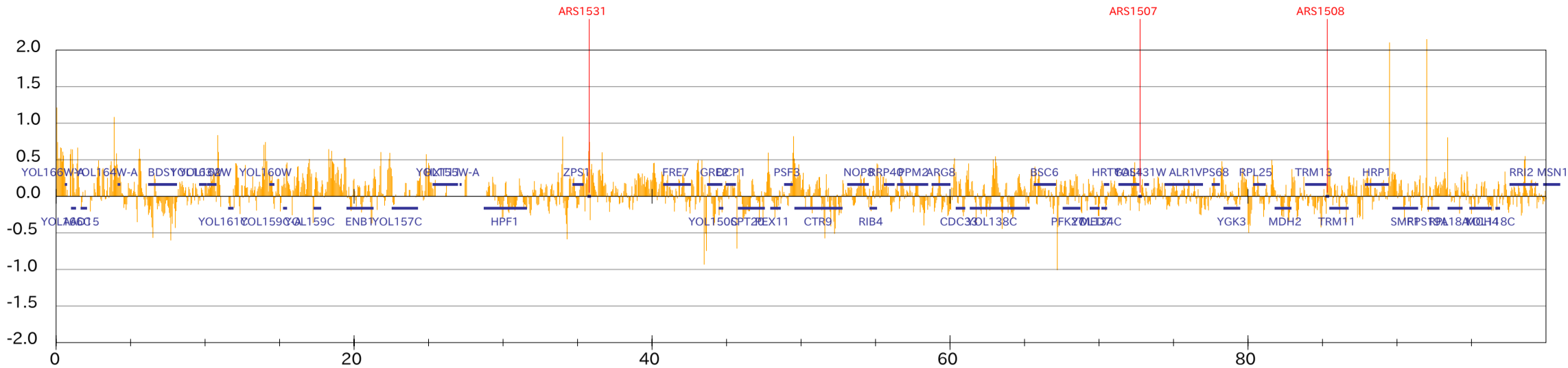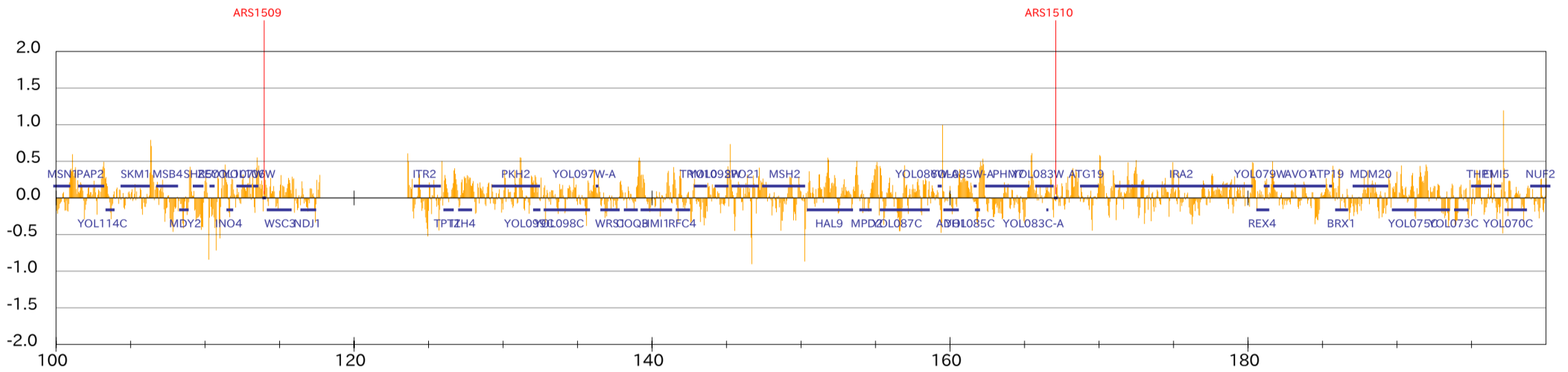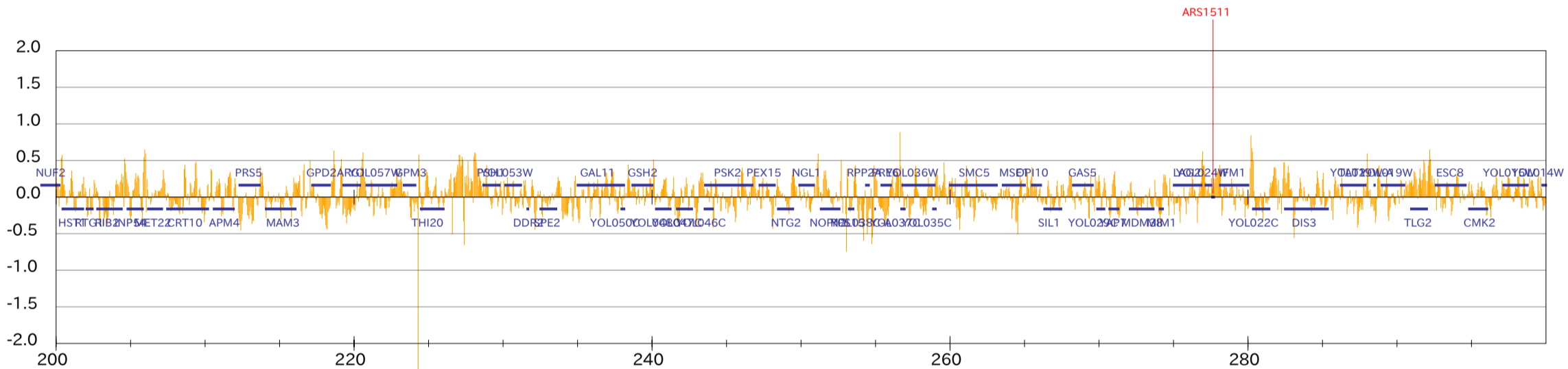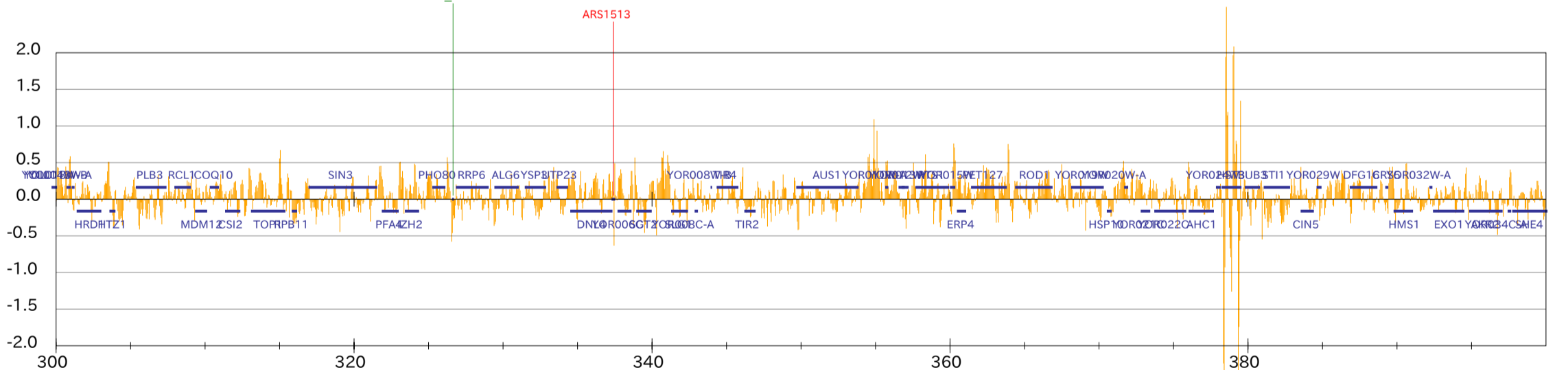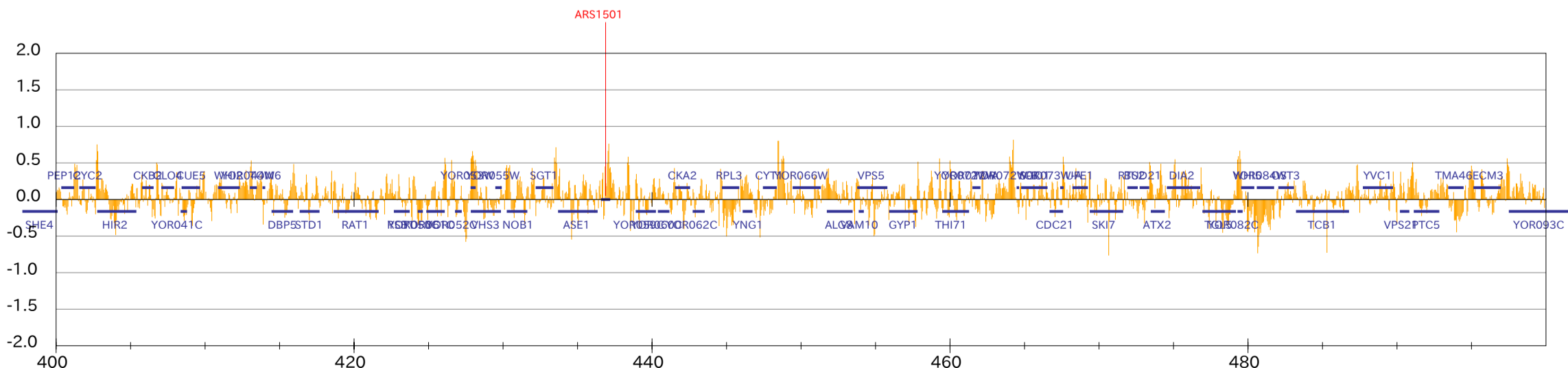

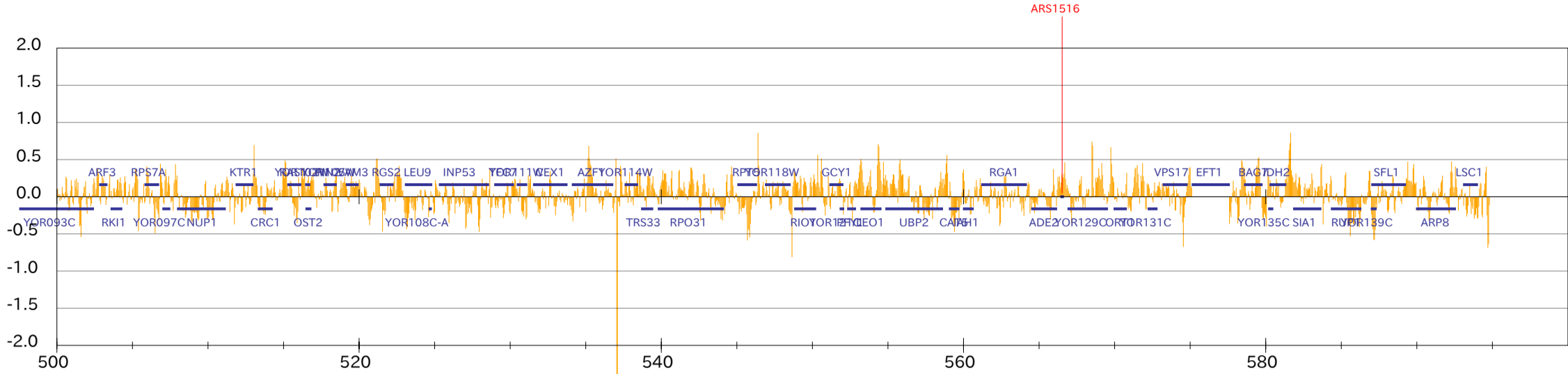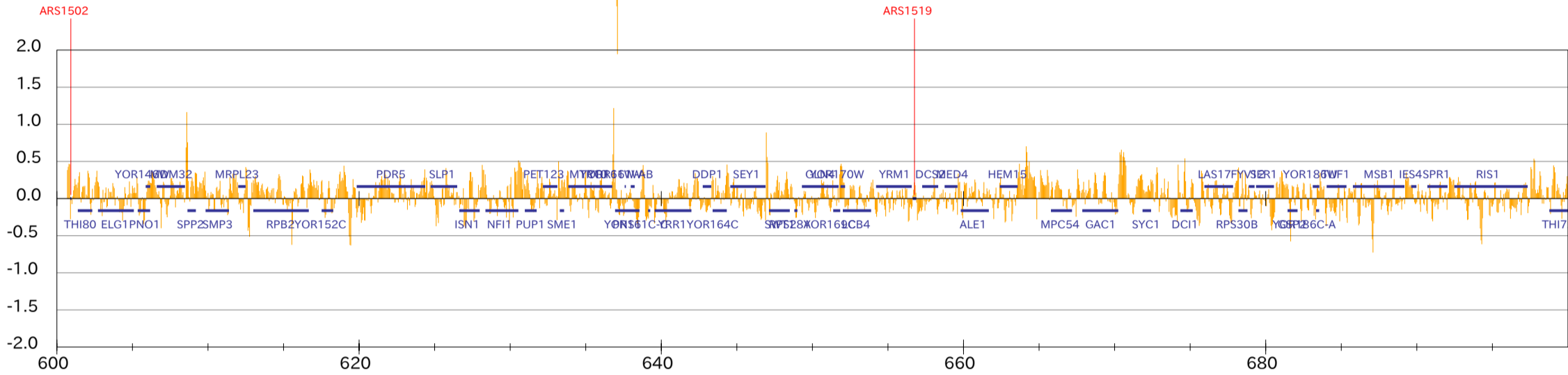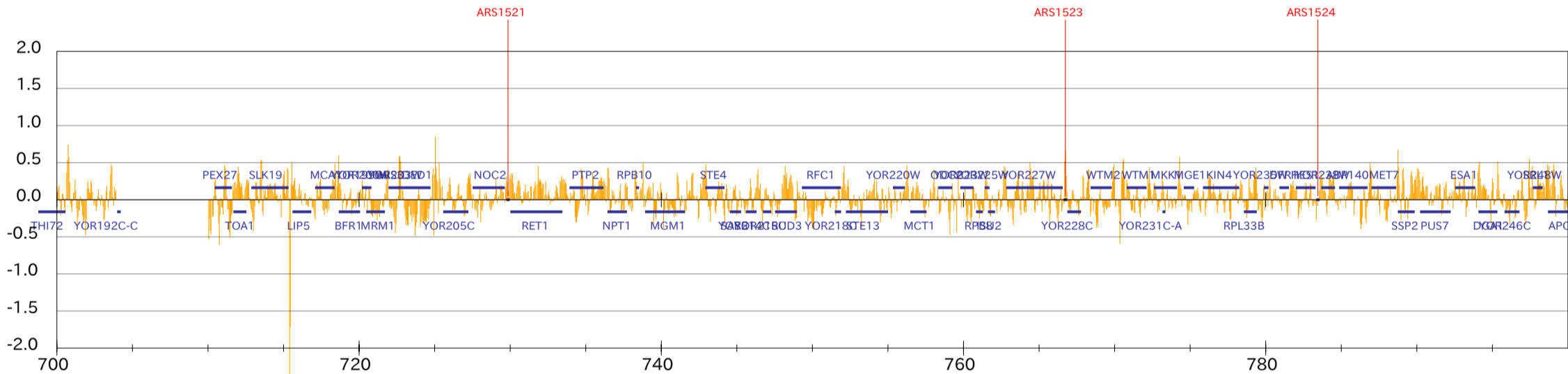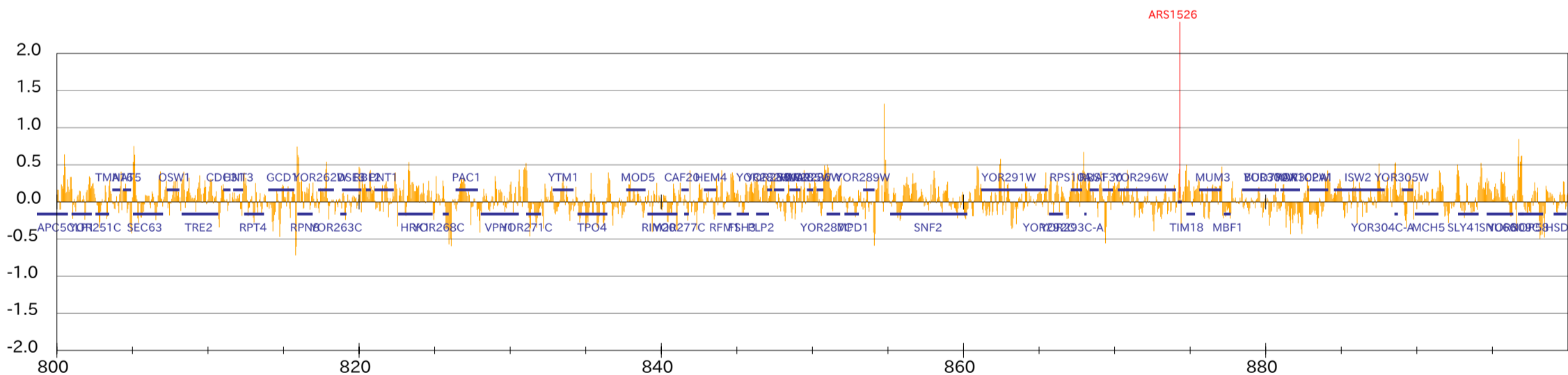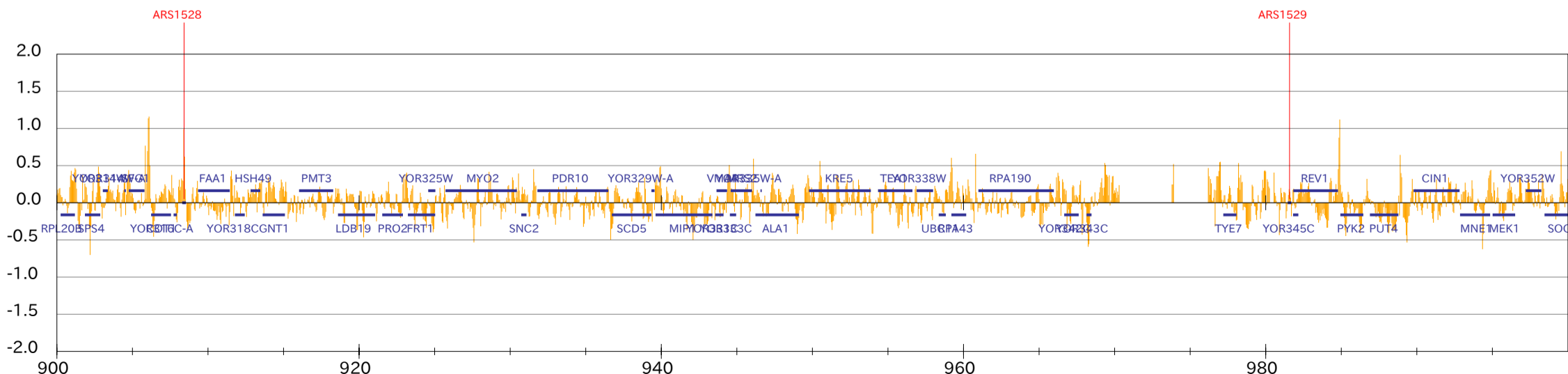

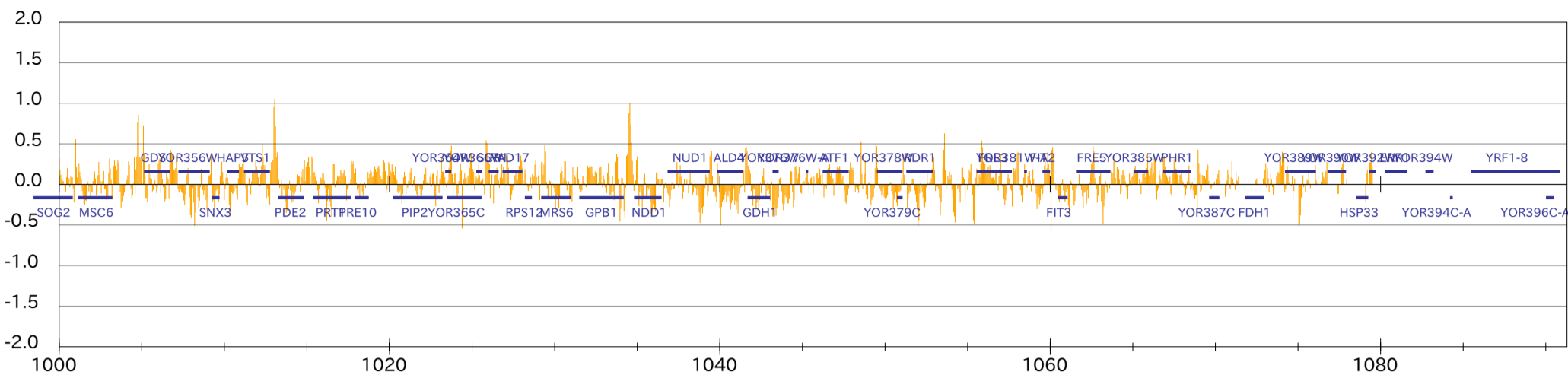

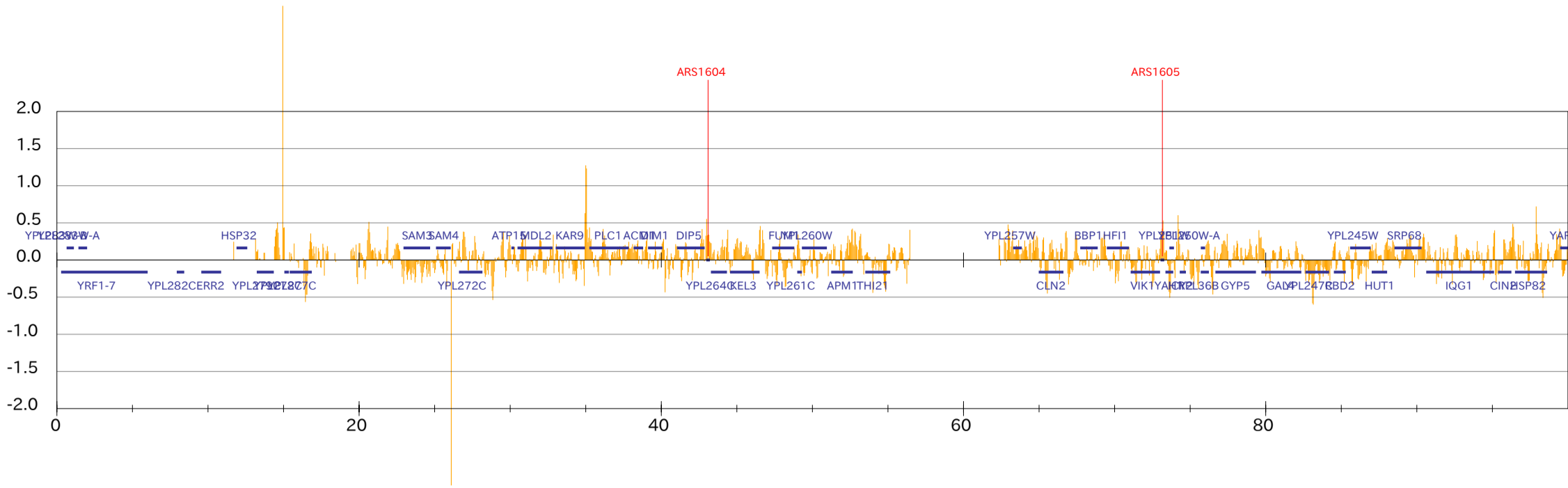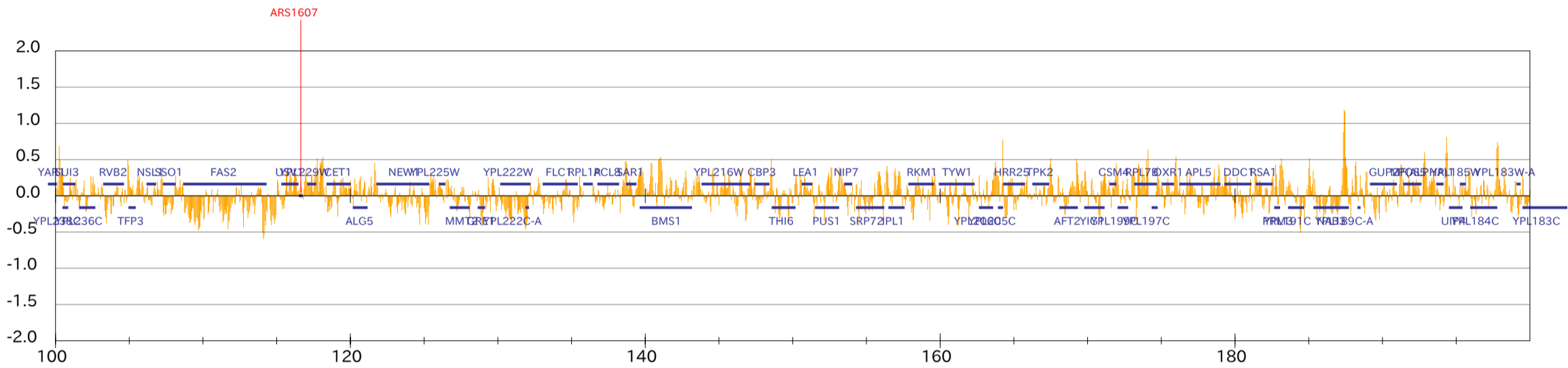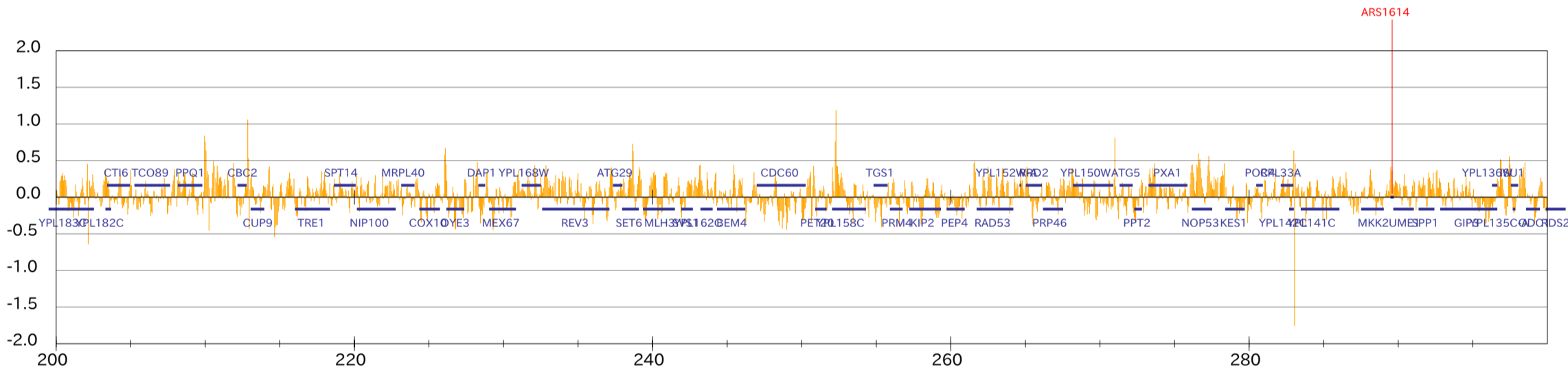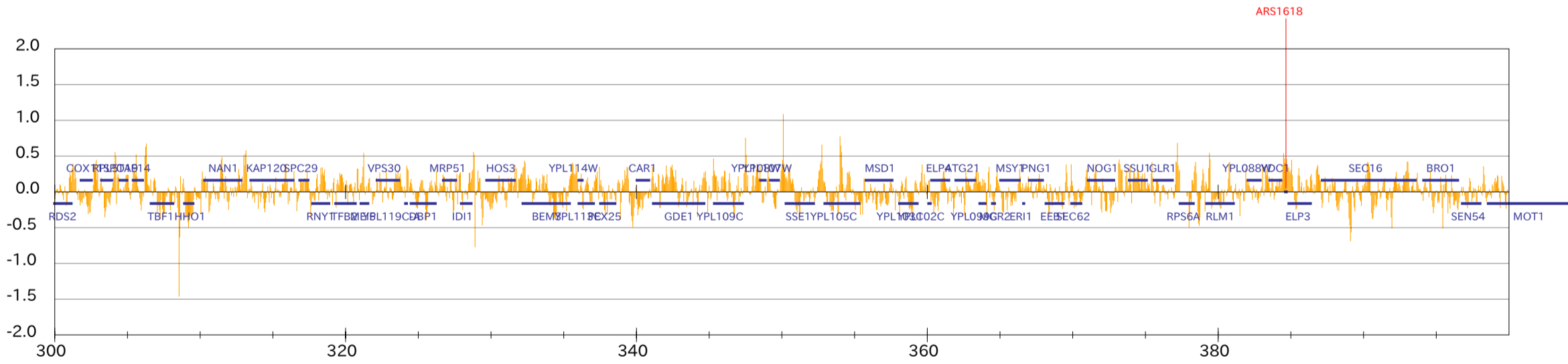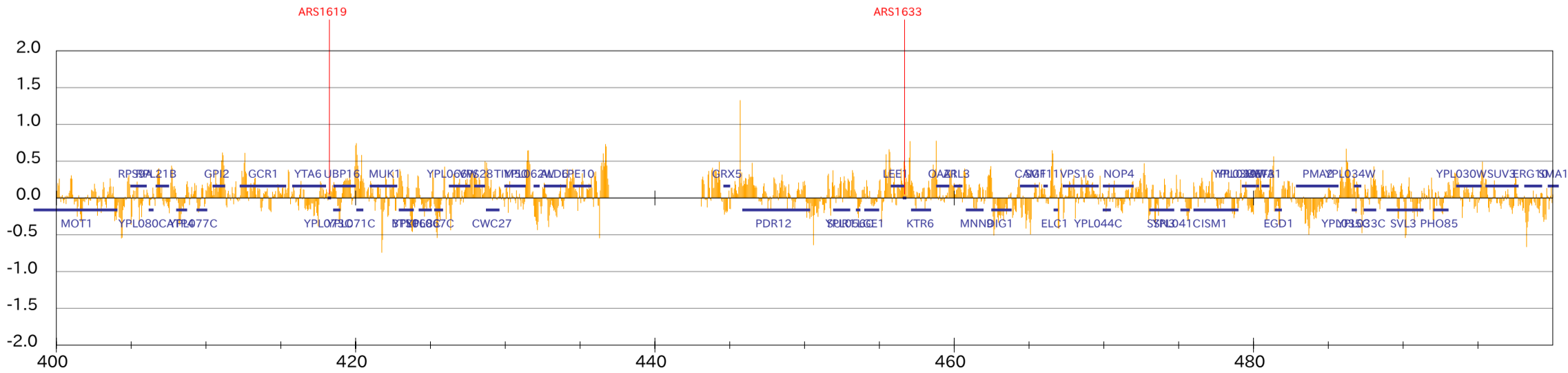

chr16\_2

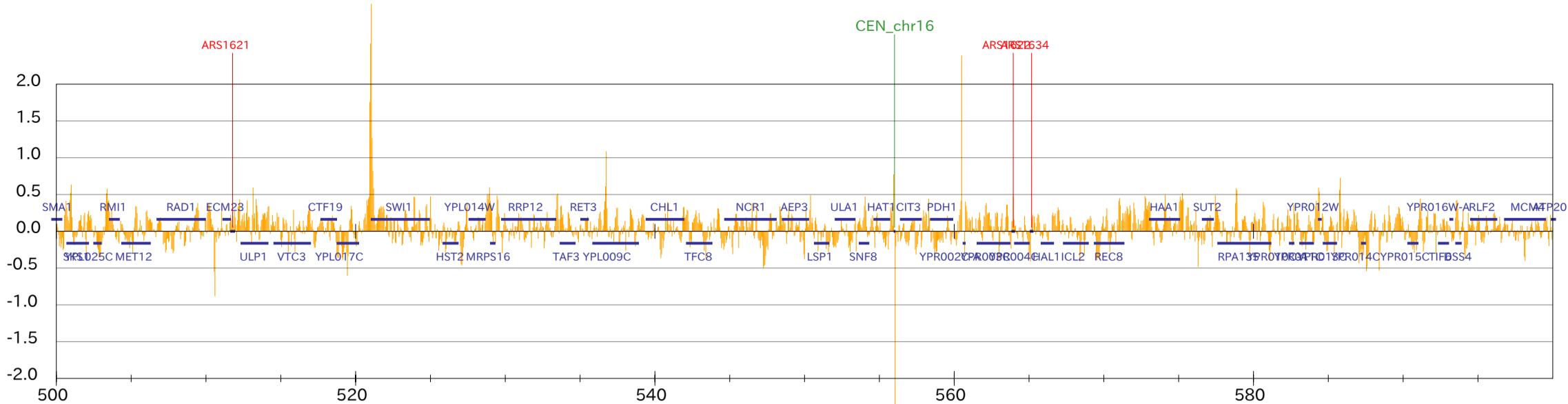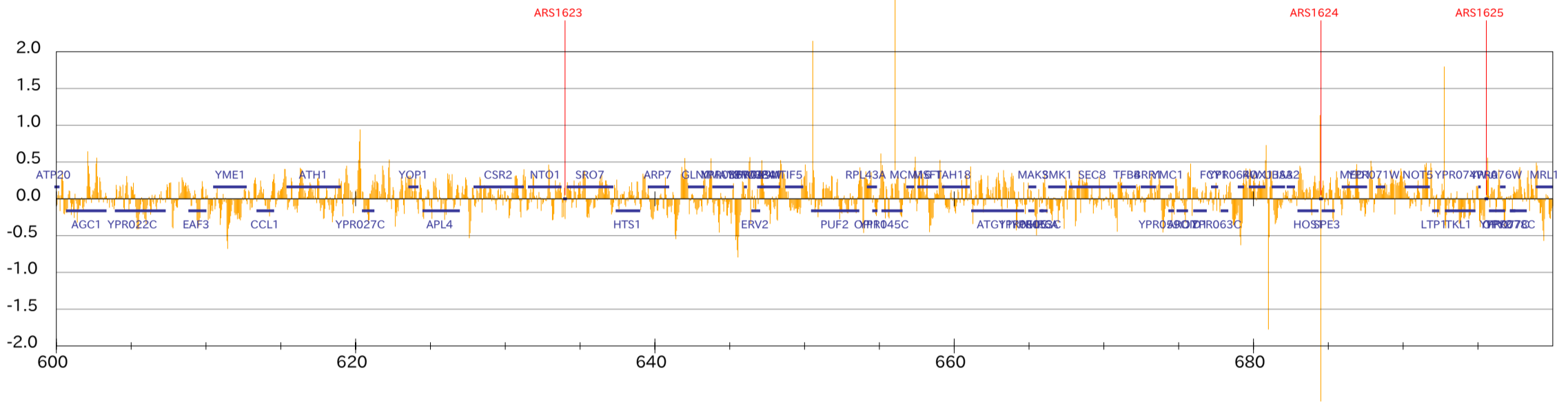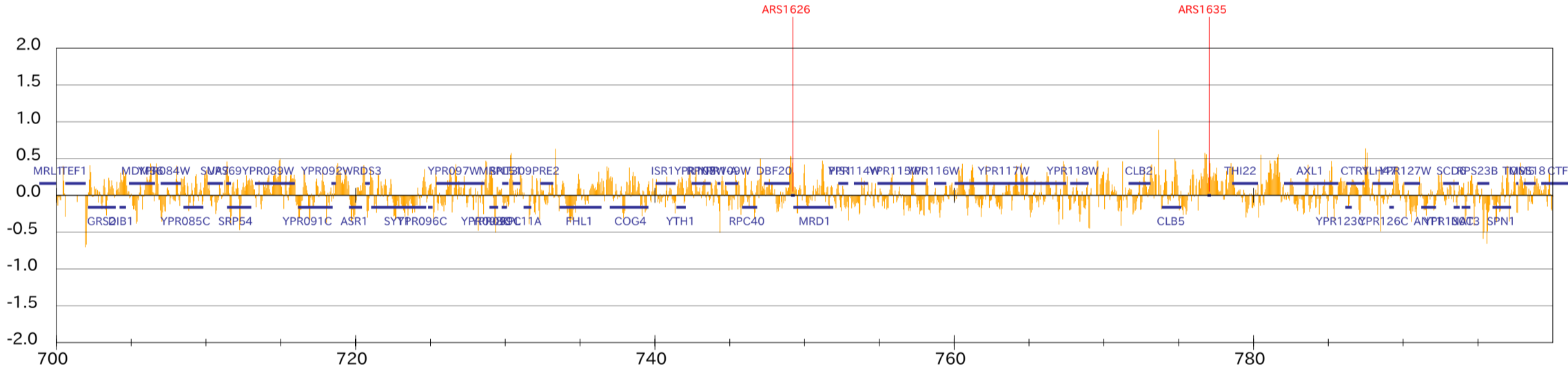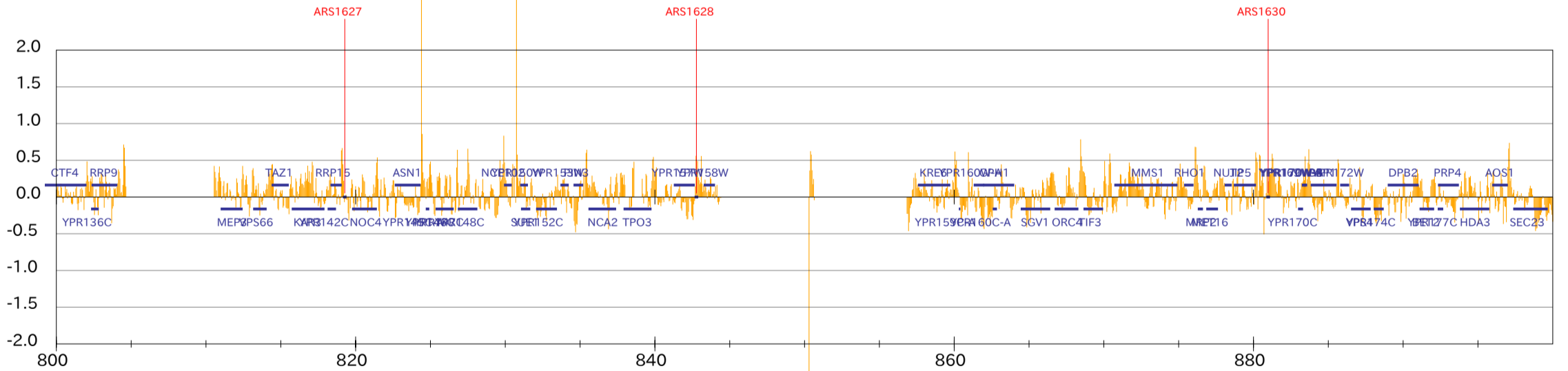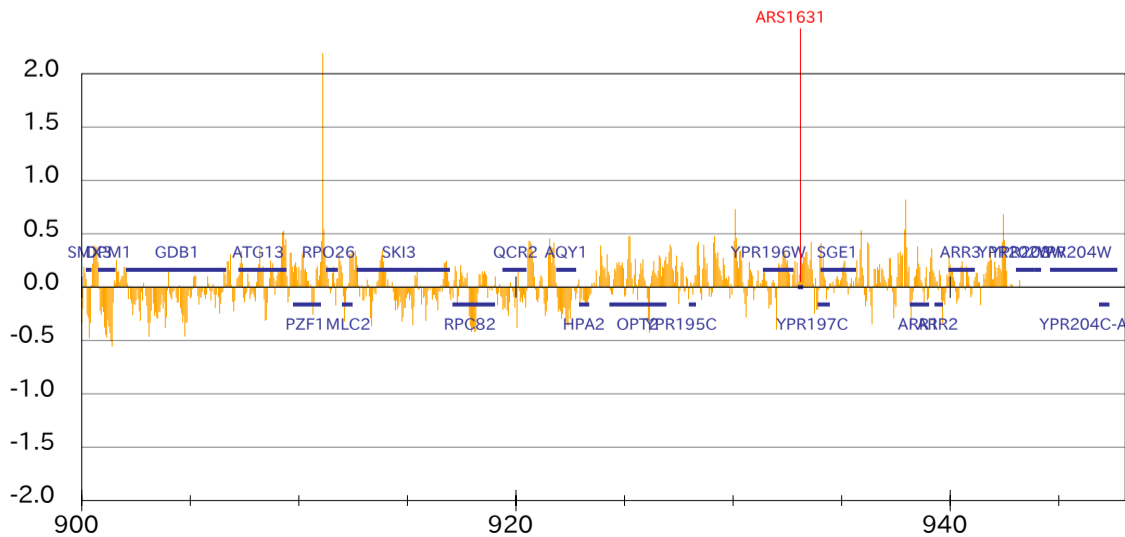

Supplement: Figure S4 — The distribution map of affinity-purified new nucleosomes harboring histone H3-G-Flag analyzed by ChIP-on-chip analysis. Blue horizontal lines indicate the open reading frames, and positive orange peaks indicate the significant binding of the proteins to the chromosome. CEN denotes the position of the centromere, and the red lines and numbers indicate the positions of autonomously replication origins (ARS). The horizontal lines indicate log 1 of the signal strength, and the vertical scale bar indicates the chromosomal coordinates in kb. (PDF) [file pone.0028980.s004.pdf]

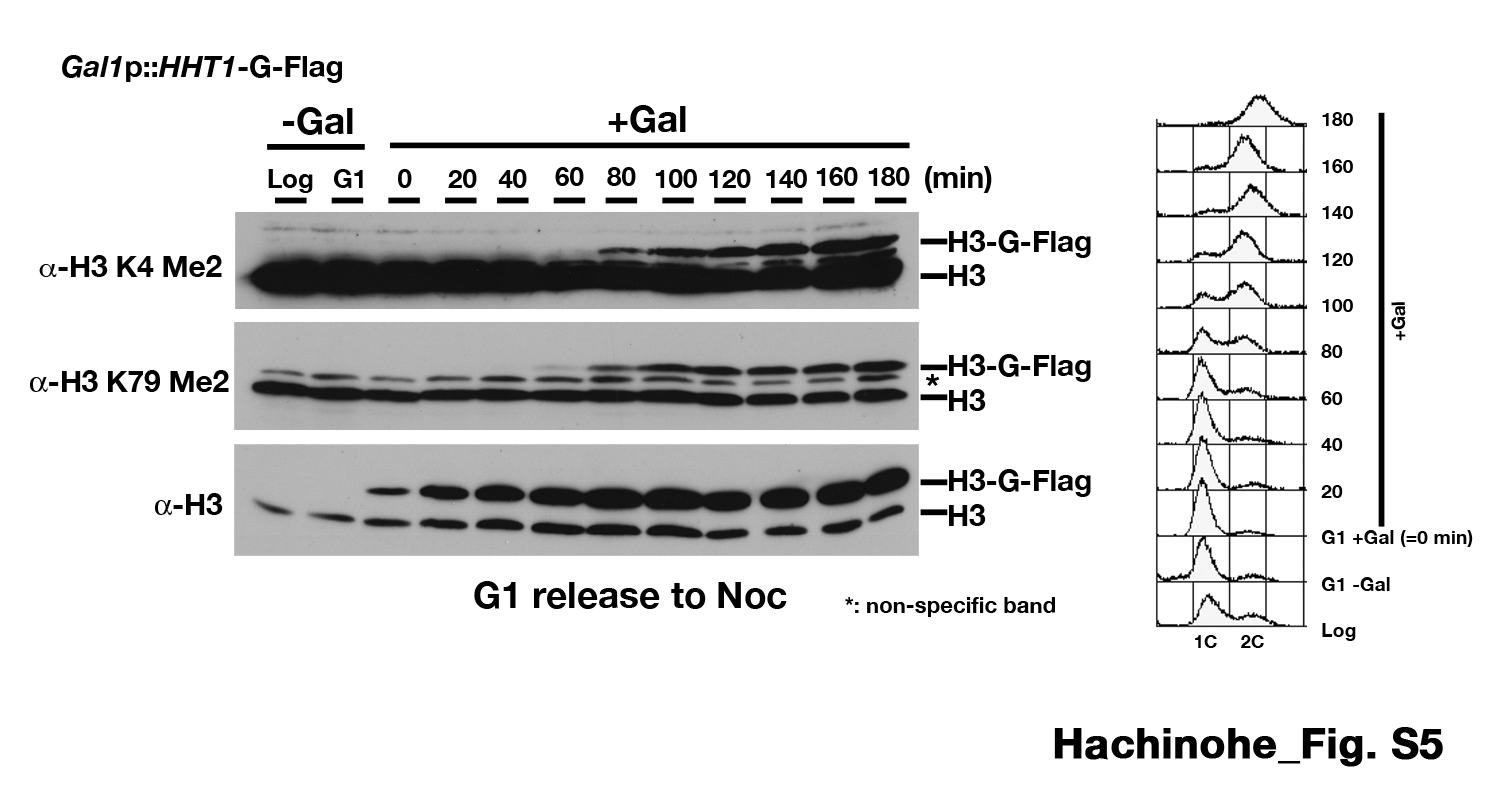

Supplement: Figure S5 — Histone methylations specific for euchromatin are introduced into new nucleosome after mid-S phase. G1-arrested cells expressing histone H3-G-Flag were released into YPR medium containing nocodazole and galactose. Cell-cycle progression was monitored by FACS analysis. Cell extracts prepared from cells at each time were analyzed by SDS-PAGE and gels were transferred to a nitrocellulose membrane. Western blotting analyses with antibodies to histone H3 dimethylated at -K4, -K79, and whole histone H3 are shown. H3-K4 and H3-K79 di-methylation were detected in histone H3-G-Flag after 80 min in time course (during mid-S phase). (TIF) [file pone.0028980.s005.tif]

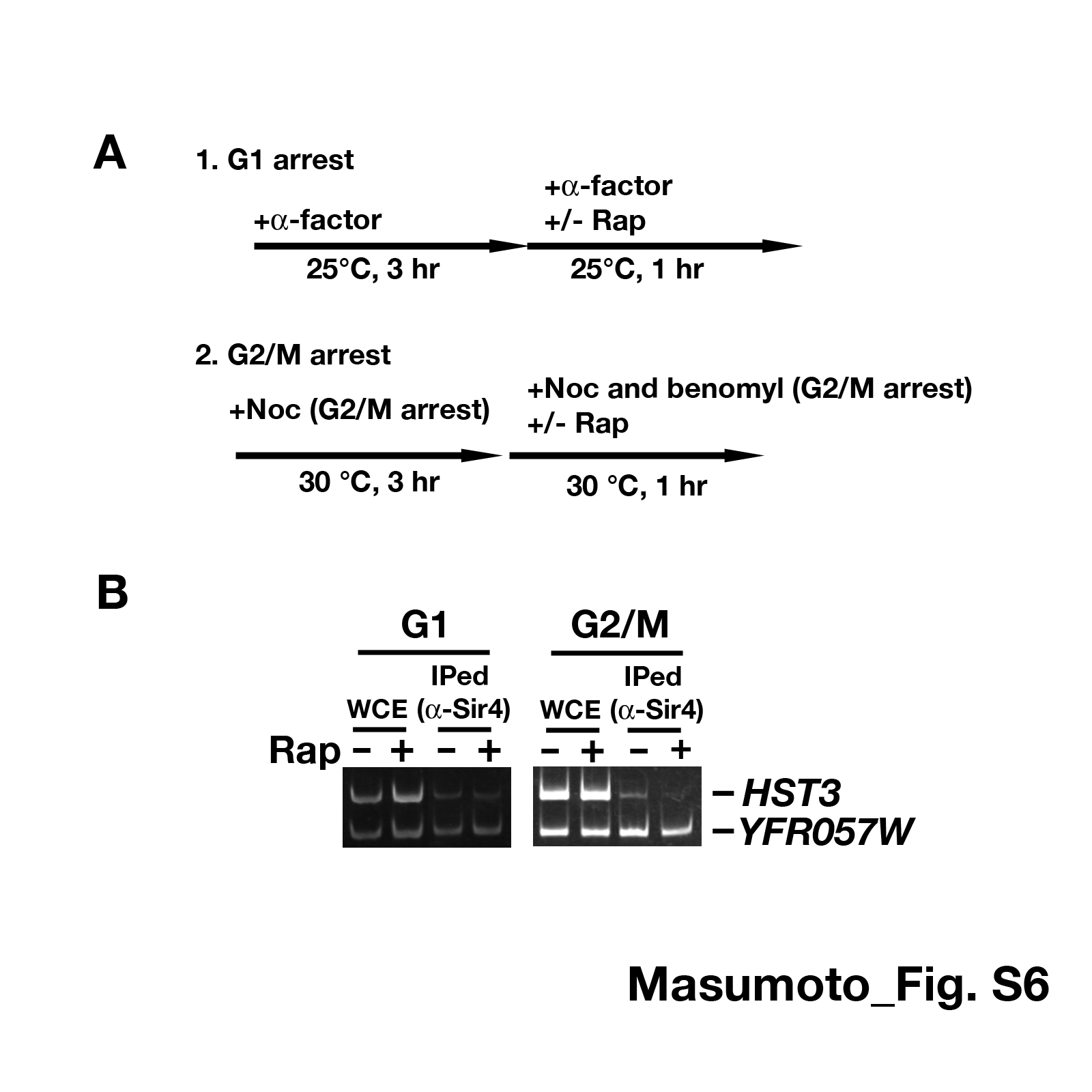

Supplement: Figure S6 — Sir4 remains bound on heterochromatin in both at G1- and G2/M-arrested sir3 -AA cells with rapamycin. (A) A scheme of procedure of treatment of rapamycin both with G1- and G2/M-arrested cells. 1. HMY733 cells were arrested at G1 phase in YPR medium containing α-factor at a final concentration of 10 µg/ml, and then further treated with 10 µg/ml α-factor in the absence or presence of 1 µg/ml rapamycin at 25°C for 1 hr. 2. HMY733 cells were arrested at G2/M phase in YPR medium containing nocodazole (Noc) at a final concentration of 10 µg/ml, and then further treated with 10 µg/ml nocodazole, 20 µg/ml benomyl, in the absence or presence of 1 µg/ml rapamycin at 30°C for 1 hr. (B) Chromatin immunoprecipitation (ChIP) analysis was applied to examine the localization of the Sir4 protein in heterochromatin. DNA isolated from immunoprecipitated chromatin (IPed) or whole-cell extracts (WCE) was quantitatively analyzed using a competitive PCR strategy, in which one set of primers amplified 80- and 155-bp products from the YFR057W locus and the HST3 locus, respectively. (TIF) [file pone.0028980.s006.tif]

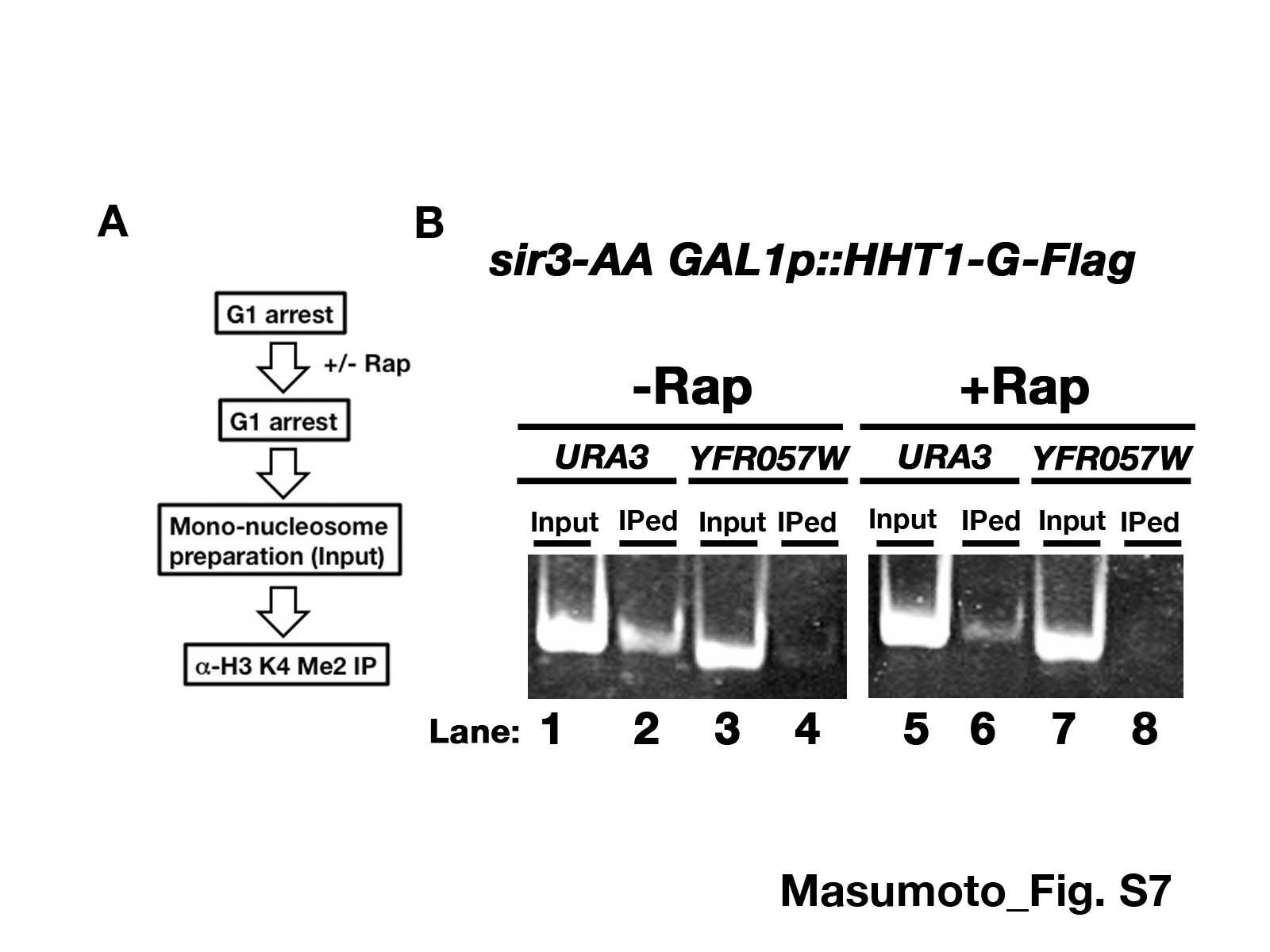

Supplement: Figure S7 — Rapamycin treatment to G1-arrested sir3 -AA cells does not induce histone H3-K4 di-methylation on heterochromatin. (A) The experimental procedure for the isolation of mononucleosomes containing Flag-tagged dimethylated histone H3-K4. (B) The localization of DNA isolated from affinity-purified mononucleosomes was analyzed by PCR as described in Fig. 3D. With or without rapamycin, histone H3-K4 di-methylation was detected at URA3 locus, but not at YFR057W locus (Lanes 2, 4, 6, and 8). (TIF) [file pone.0028980.s007.tif]
