# Supplementary material for: The Inheritance of Histone Modifications Depends upon the Location in the Chromosome in Saccharomyces cerevisiae
Source: PLoS One. 2011 Dec 21;6(12):e28980. doi: 10.1371/journal.pone.0028980 (PMC3244422; doi:10.1371/journal.pone.0028980)
Supplement: Methods S1 — Methods of preparations of yeast chromatin and mononucleosome, primer sequences, plasmids and yeast strains. (DOCX) [file pone.0028980.s008.docx]

**Supporting Method S1**

**Preparation of yeast chromatin**.

Yeast chromatin was prepared as described previously [1]. Briefly, mid-log phase cells (~1.0×10^10^ cells) were harvested, washed with water, and resuspended in 5 ml of Y1 buffer (1 M sorbitol, 100 mM EDTA, pH 8.0, 14.3 mM ß-mercaptoethanol (BME)) with 50 µl of 10 mg/ml Zymolase-100T (Seikagaku). The cell suspension was incubated at 30°C for 45 min, and cells were collected by centrifugation at 5,000 rpm for 5 min at 4°C. Cells were washed twice in 1 M sorbitol and resuspended in 10 ml of lysis buffer (18% Ficoll 400, 20 mM KPO_4_, pH 7.5, 1 mM MgCl_2_, 0.25 mM EDTA, 0.25 mM EGTA, and 1× Complete Protease Inhibitor Cocktail (Roche)). The cell suspension was incubated for 5 min on ice and the cells were collected by centrifugation at 14,000 rpm for 5 min at 4°C using a JA-20 rotor (Beckman-Coulter). The cells were resuspended in 10 ml of NP-40 buffer (10 mM Tris-Cl, pH 8.0, 75 mM NaCl, 0.5% NP-40, 1 mM phenylmethylsulfonyl fluoride (PMSF), and 1× Complete Protease Inhibitor Cocktail) and centrifuged at 14,000 rpm for 5 min at 4°C using a JA-20 rotor. Resuspension and centrifugation were repeated and then the cells were suspended in 10 ml of NP-40 buffer containing 500 mM NaCl, and centrifuged at 14,000 rpm for 5 min at 4°C (JA-20 rotor). The chromatin pellet was resuspended in 2 ml of Buffer B (100 mM Tris-Cl, pH 8.0, 50 mM NaCl, 10 mM MgCl_2_, 3 mM DTT, 2 mM EDTA, 20% glycerol, 1 mM PMSF, 2 mg/ml pepstatin A, and 2 mg/ml leupeptin) and frozen at -80°C.

**Isolation of yeast mononucleosomes**.

Chromatin was prepared as described above (5 mg) and suspended in 1 ml of micrococcal nuclease (MNase) buffer (20 mM Tris-Cl, pH 7.5, 5 mM CaCl_2_, 150 mM NaCl, 2 mg/ml pepstatin A, and 2 mg/ml leupeptin). One hundred and forty units of MNase (Takara Bio) was added, the solution was incubated at 37°C for 5 min, and the reaction was stopped by the addition of 30 µl 0.5 M EDTA (pH 8.0). After centrifugation at 12,000 rpm for 5 min at 4°C, the supernatant that was recovered, which contained mononucleosomes and oligonucleosomes, was fractionated using a 10−30% sucrose gradient (total volume: 5 ml) and centrifugation at 50,000 rpm for 6 hr at 4°C using a SW55.Ti rotor (Beckman-Coulter). Fractions (300 µl each) were collected from top to bottom by pipetting. To identify the fractions that contained mononucleosomes, 50 µl of each fraction was removed and DNA was purified using Wizard DNA clean-up (Promega). Purified DNA samples were then subjected to gel electrophoresis in a 1.5% agarose gel in 1× Tris-boric acid-EDTA (TBE) buffer. Since the characteristic size for DNA obtained from mononucleosomes is 160 bp, chromatin fractions containing 160 bp DNA fragments were collected and stored at -80°C.

**Primer sequences used in this study**

*URA3* locus:

5'−CTACTCATCCTAGTCCTGTTGCTGC -3’ 5'−GGTACGAACATCCAATGAAGCACAC -3'

*YFR057W* locus:

5'−TATAGTAAGTGCTCGGCCAAGTCAAG−3' 5'−AACTTTGATCCTTACTCGTGATATT−3'

*HST3* locus:

5’-GTTCTCCGGCGCGTAACCAAGCAGTTGAGC-3’ 5’-GTTCTCCGGCGCGTAACCAAGCAGTTGAGC-3’

**Supplementary Table I.**

Plasmids

Name Genotype Reference

PHM286 *HST3*-13myc and *ADE3* with each own promoter in YCp33 This study

PHM371 P_GAL1_::*HHT1*-glycine linker-FLAG in YIp204 This study

PHM493 *HHF1, HHT1,* both with their own native promoter in pRS413 This study

PHM502 *HHF1, hht1* K4R, both with their own native promoter in pRS413 This study

pMK43 *IAA17* domain-tagged to the C-terminus of the target gene (*kan*MX4) [3]

pFA6a-FRB-KanMX6 *FRB* tagged to the C-terminus of the target gene [4]

**Supplementary Table II.**

Yeast strains

Strain Genotype Reference

W303-1a *MATa ade2-1 can1-100 his3-11,15 leu2-3,112 trp1-1 ura3-1* [5]

DY5733 *MATa (hht2-hhf2)*::*kan*MX3 *(hht1-hhf1)*::*LEU2*

+ [YCp50-*HHT2-HHF2 (URA3)]* [6]

HHY168 *MATa* *tor1-1 fpr1::NAT RPL13A-2xFKBP12::TRP1* [4]

HMY210 *MATa hst3∆::his5+* This study

HMY278 *MATa hst3∆::his5+hst4∆::kanMX3 ade3::hisG +*[PHM286] This study

YNK55 *MATa ura3::ADH1promoter-ScSKP1-osTIR1-2-9myc (URA3)* [3]

HMY616 *MATa ura3::PGAL1-HHT1-G linker-*FLAG *(URA3)* This study

HMY733 *MATa tor1-1 fpr1∆::NAT RPL13A-2xFKBP12::TRP1 ura3::GAL1p-HHT1-G linker FLAG-Thht1 (URA3) sir3::SIR3-FRB (kanMX3)* This study

HMY837 *MATa ura3::ADH1promoter-ScSKP1-osTIR1-2-9myc (URA3) hst4-IAA17 (kanMX4) hst3∆::his5+* *trp1::PGAL1-HHT1-G linker-*FLAG *(TRP1)* This study

HMY963 *MATa (hht2-hhf2)*::*kan*MX3 *(hht1-hhf1)*::*LEU2* *trp1::PGAL1- HHT1-G linker-*FLAG *(TRP1)* + [PHM502] This study

HMY964 *MATa (hht2-hhf2)*::*kan*MX3 *(hht1-hhf1)*::*LEU2*  *trp1::PGAL1- HHT1-G linker-*FLAG *(TRP1)* + [PHM493] This study

**Reference**

1. Wechser MA, Kladde MP, Alfieri JA, Peterson CL (1997) Effects of Sin- versions of histone H4 on yeast chromatin structure and function. EMBO J 16: 2086-2095.

2. Trelles-Sticken E, Adelfalk C, Loidl J, Scherthan H (2005) Meiotic telomere clustering requires actin for its formation and cohesin for its resolution. J Cell Biol 170: 213-223.

3. Nishimura K, Fukagawa T, Takisawa H, Kakimoto T, Kanemaki M (2009) An auxin-based degron system for the rapid depletion of proteins in nonplant cells. Nat Methods 6: 917-922.

4. Haruki H, Nishikawa J, Laemmli UK (2008) The anchor-away technique: rapid, conditional establishment of yeast mutant phenotypes. Mol Cell 31: 925-932.

5. Thomas BJ, Rothstein R (1989) Elevated recombination rates in transcriptionally active DNA. Cell 56: 619-630.

6. Wittschieben BO, Fellows J, Du W, Stillman DJ, Svejstrup JQ (2000) Overlapping roles for the histone acetyltransferase activities of SAGA and elongator in vivo. Embo J 19: 3060-3068.
